# MAPLE: interpretable deep learning identifies selective antimicrobial peptides using joint evolutionary–physicochemical analysis

**DOI:** 10.1093/bib/bbag318

**Published:** 2026-06-17

**Authors:** Hao Liu, Yi Shi, Feiyu Guo, Jinyi Wang, Jiaqian Li, Guangji Wang, De-Chuan Zhan, Haiping Hao, Guo Yu

**Affiliations:** State Key Laboratory of Natural Medicines, Key Laboratory of Drug Metabolism, China Pharmaceutical University, No. 24 Tongjiaxiang, Gulou District, Nanjing 210009, China; School of Basic Medicine and Clinical Pharmacy, China Pharmaceutical University, No. 639 Longmian Avenue, Jiangning District, Nanjing 211198, China; State Key Laboratory of Natural Medicines, Key Laboratory of Drug Metabolism, China Pharmaceutical University, No. 24 Tongjiaxiang, Gulou District, Nanjing 210009, China; Institute of Innovative Drug, China Pharmaceutical University, No. 639 Longmian Avenue, Jiangning District, Nanjing 211198, China; National Key Laboratory for Novel Software Technology, Nanjing University, No. 163 Xianlin Avenue, Qixia District, Nanjing 210023, China; School of Artificial Intelligence, Nanjing University, No. 163 Xianlin Avenue, Qixia District, Nanjing 210023, China; School of Basic Medicine and Clinical Pharmacy, China Pharmaceutical University, No. 639 Longmian Avenue, Jiangning District, Nanjing 211198, China; School of Basic Medicine and Clinical Pharmacy, China Pharmaceutical University, No. 639 Longmian Avenue, Jiangning District, Nanjing 211198, China; School of Basic Medicine and Clinical Pharmacy, China Pharmaceutical University, No. 639 Longmian Avenue, Jiangning District, Nanjing 211198, China; State Key Laboratory of Natural Medicines, Key Laboratory of Drug Metabolism, China Pharmaceutical University, No. 24 Tongjiaxiang, Gulou District, Nanjing 210009, China; National Key Laboratory for Novel Software Technology, Nanjing University, No. 163 Xianlin Avenue, Qixia District, Nanjing 210023, China; School of Artificial Intelligence, Nanjing University, No. 163 Xianlin Avenue, Qixia District, Nanjing 210023, China; State Key Laboratory of Natural Medicines, Key Laboratory of Drug Metabolism, China Pharmaceutical University, No. 24 Tongjiaxiang, Gulou District, Nanjing 210009, China; Institute of Innovative Drug, China Pharmaceutical University, No. 639 Longmian Avenue, Jiangning District, Nanjing 211198, China; State Key Laboratory of Natural Medicines, Key Laboratory of Drug Metabolism, China Pharmaceutical University, No. 24 Tongjiaxiang, Gulou District, Nanjing 210009, China; School of Basic Medicine and Clinical Pharmacy, China Pharmaceutical University, No. 639 Longmian Avenue, Jiangning District, Nanjing 211198, China

**Keywords:** antimicrobial peptides, therapeutic selectivity, hemolysis prediction, protein language models, functional profiling, motif interpretability

## Abstract

Antimicrobial peptides (AMPs) are promising alternatives to conventional antibiotics, yet early translation is often hindered by the perceived coupling between antibacterial potency and mammalian toxicity. This assumption complicates prioritization: highly active candidates are frequently suspected to be hemolytic, while existing multi-task predictors rarely reveal where selectivity resides in sequence space. Here, we present Multifunctional AMP Learning Engine (MAPLE), an interpretable dual-stream framework for AMP identification and systematic category-specific functional profiling across 14 activity categories directly from peptide sequences. MAPLE combines protein language model embeddings with explicit physicochemical descriptors, enabling robust task-specific prediction under severe label imbalance. Across the benchmark dataset and a sequence-non-overlapping independent validation set, MAPLE achieves consistently well-balanced performance, including on low-prevalence but clinically relevant endpoints. Building on this predictive basis, we conduct systematic k-mer enrichment to map motif-level selectivity and show that potency–hemolysis coupling is motif-regime-dependent rather than universal. Motifs most strongly enriched for antibacterial activity exhibit reduced hemolytic overlap and occupy a physicochemical regime characterized by moderate cationicity, lower hydrophobicity, and higher amphipathicity. We further provide a proof-of-concept prioritization workflow leveraging antibacterial-selective motifs, with structural modeling yielding conformations consistent with amphipathic α-helices. Despite limitations of predominantly binary annotations and incomplete structural integration, MAPLE offers reproducible sequence-level hypotheses and prioritization principles to support the engineering of potent and safer AMPs.

## Introduction

Antimicrobial resistance (AMR) poses a growing threat to global health. In 2019, bacterial AMR was estimated to be directly responsible for 1.27 million deaths and associated with 4.95 million deaths worldwide, with the burden falling disproportionately on resource-limited settings [1, 2]. Compounding this challenge, few new antibiotic classes have been introduced over the past few decades [2]. Against this backdrop, antimicrobial peptides (AMPs) have emerged as promising alternatives to conventional antibiotics. These short, typically cationic peptides can kill bacteria by perturbing membrane integrity through physicochemical interactions and are generally considered less prone to inducing resistance [3–6]. The growing experimental validation of AMP libraries further supports their therapeutic potential [7–10]. However, many AMP candidates are limited by hemolysis and cytotoxicity, which narrows the therapeutic window and makes therapeutic selectivity a central bottleneck in AMP development [11–14].

Before peptide candidates can be prioritized for synthesis and experimental testing, computational models are often used to support large-scale virtual screening. Early AMP screening methods mainly relied on feature-engineered machine learning models, such as support vector machines and random forests, which remain widely used as practical baselines [15–18]. However, as AMP datasets have expanded in size and heterogeneity, these approaches have become increasingly constrained by shallow representations that are often insufficient to capture the nonlinear and context-dependent sequence–function relationships underlying antibacterial activity and therapeutic selectivity [19]. Recent advances in deep learning have substantially broadened the computational toolkit for AMP discovery, enabling models to learn sequence representations directly from primary structures and improving the efficiency of activity prediction and candidate prioritization [20–24]. Generative modeling has further made it possible to explore vast peptide sequence spaces more systematically and to propose candidates with desired antibacterial properties [25–29]. In parallel, progress in structure prediction, particularly with AlphaFold, has made structure-guided hypothesis generation more accessible, complementing sequence-based analyses with information on peptide conformations and potential membrane-related interactions [30–32]. At the same time, convolutional and recurrent neural architectures have been widely applied to AMP prediction to capture local motifs and longer-range sequence dependencies [33–35].

Protein language models (PLMs) represent an important advance in biological sequence modeling. Trained on large-scale protein corpora, these models learn representations that capture broad evolutionary regularities and other biologically meaningful constraints embedded in sequence space. ESM-2, for example, was trained on 250 million protein sequences and has shown strong performance across downstream tasks such as structure prediction and functional annotation [36]. Building on these advantages, PLM-derived embeddings have been increasingly adopted in AMP prediction and have delivered competitive results across multiple benchmark datasets [37–41]. Some recent studies have further combined PLM embeddings with task-specific prediction heads or attention modules to improve residue-level interpretability and to highlight sequence regions potentially associated with function [37–39].

Despite these advances, many AMP predictors remain centered on binary activity classification or treat efficacy- and toxicity-related endpoints as parallel outputs without explicitly modeling their relationship within a unified framework [42, 43]. In practical screening, however, candidate peptides must be prioritized not only for antibacterial potency but also for compatibility with host safety constraints. This selectivity is often summarized by the selectivity index (SI), typically defined as the ratio of a host cytotoxic concentration to the minimum inhibitory concentration (MIC), with higher values indicating a wider therapeutic window [11, 12, 14, 44]. At the sequence level, cationicity, amphipathicity, and hydrophobicity jointly influence membrane targeting and disruption [45–47], yet how these properties differentially contribute to bacterial killing versus mammalian membrane damage remains incompletely resolved. Consequently, although antibacterial potency and hemolysis are often assumed to be coupled, this relationship has seldom been quantified in a way that yields operational, sequence-level guidance [48, 49]. The observed coupling may reflect genuine biophysical constraints, but it may also be amplified by biased sampling of particular motif classes and limited exploration of broader selective sequence space [50, 51].

To extend functional coverage beyond single-endpoint prediction, recent AMP predictors have increasingly adopted multi-label learning and shared representation training [52–55]. Representative examples include iAMPCN, which applies deep convolutional architectures to multifunctional annotation [54], and TransImbAMP, which combines transformer-based sequence modeling with asymmetric loss to improve sensitivity for rare functions under severe class imbalance [52]. Other studies have explored alternative sequence encodings, including natural language processing-derived amino-acid embeddings [55], or aimed to develop transferable architectures across multiple peptide bioactivity prediction tasks [53]. These efforts have broadened the scope of AMP prediction from binary identification toward more comprehensive functional profiling. At the same time, attention-based interpretation has begun to provide preliminary residue-level clues to sequence–function relationships [41, 56], while related work in bioinformatics suggests that more structured inductive biases may further improve model interpretability [57]. Nevertheless, most of these frameworks remain oriented toward endpoint expansion rather than explicit characterization of therapeutic selectivity across efficacy and toxicity dimensions.

Despite these advances, Artificial Intelligence (AI)-driven AMP modeling still faces several obstacles that limit clinically actionable discovery. Severe class imbalance across functional endpoints weakens generalization for rare but clinically relevant activities, such as anti-methicillin-resistant Staphylococcus aureus (MRSA), antiparasitic, and antibiofilm functions [58, 59]. In addition, most multi-label predictors treat efficacy- and toxicity-related endpoints as parallel outputs rather than as a selectivity problem, limiting their ability to prioritize peptides with favorable efficacy–toxicity profiles [22, 42, 43]. Interpretability also remains incomplete: attention- or saliency-based analyses may highlight important residues, but they rarely yield discrete, functionally distinct motifs that can be directly translated into peptide engineering rules [22, 58, 59]. It remains insufficiently characterized whether antibacterial motif enrichment correlates monotonically with hemolytic enrichment or whether strongly antibacterial motifs can exhibit reduced toxicity relative to moderately enriched motifs [60, 61].

To address these challenges, we present the Multifunctional AMP Learning Engine (MAPLE), a dual-stream framework that profiles AMP identity and 14 functional activities directly from sequence. MAPLE integrates ESM-2 evolutionary embeddings with knowledge-enhanced physicochemical features and refines predictions through parallel modules: CARE (Conservative Adaptive Residual Encoder) captures conserved local motifs and ProBiMamba captures distributed sequence-level dependencies. The two streams are fused through cross-modal attention to form a peptide-level representation for label-specific prediction. Across the benchmark dataset and a sequence-non-overlapping independent validation set, we show that MAPLE maintains balanced performance despite severe label imbalance, and we demonstrate consistent improvements over representative baselines for both AMP identification and category-specific functional prediction. Building on this predictive foundation, our systematic k-mer enrichment analysis reveals a motif-regime-dependent efficacy–toxicity coupling pattern: motifs most strongly enriched for antibacterial activity exhibit reduced hemolytic overlap compared with moderately enriched motifs, yielding mechanistic hypotheses and actionable sequence-level design rules. Finally, we demonstrate a proof-of-concept generation and screening workflow that prioritizes candidates enriched for selective motifs, illustrating MAPLE’s practical utility for rational AMP design.

## Materials and methods

### Dataset construction

We considered two prediction settings for AMPs: binary AMP identification and functional activity prediction across 14 AMP-related categories, including anti-mammalian cells, antibacterial, antibiofilm, anticancer, antifungal, antigram-negative, antigram-positive, anti-human immunodeficiency virus (HIV), anti-MRSA, antioxidant, antiparasitic, antiviral, cytotoxic, and hemolytic activities. For functional profiling, each category was treated as an independent binary prediction task. The same MAPLE architectural design was applied across all labels, while each label-specific classifier was trained independently. The overall dataset construction workflow is shown in Supplementary Fig. S1, and detailed curation procedures are provided in the Supplementary Methods.

The benchmark dataset was developed by refining the original iAMPCN [54] resource through integration of functional annotations from nine curated AMP-related databases published or updated between 2021 and 2024 [7–10, 62–66], as summarized in Supplementary Table S1. Functional labels were harmonized into binary annotations, where “1” denotes experimentally supported activity and “0” denotes absence of harmonized positive evidence under the dataset-specific annotation rules. The final benchmark dataset comprised 25 507 AMPs and 72 606 non-AMPs (Supplementary Table S2).

For independent evaluation, we constructed an independent validation dataset designed to be nonredundant with the benchmark set. Positive samples were collected from the same curated databases, and sequences with 100% identity to the benchmark data were removed using CD-HIT [67–69]. To further reduce internal redundancy, CD-HIT was reapplied at a 40% identity threshold within the validation set. Negative samples were obtained from UniProt after excluding entries annotated with terms related to the 14 functional categories or broader keywords such as “toxic,” “membrane,” or “antibiotic” [70–73]. Additional quality filters retained sequences of 5–200 amino acids, removed ambiguous residues (B, J, O, U, X, and Z), and controlled sequence redundancy below 40% identity. The final independent validation dataset comprised 24 582 AMPs and 36 653 non-AMPs (Table 1). Label prevalence, co-annotation patterns, sequence length distributions, and amino acid composition profiles are summarized in Supplementary Figs S2–S5.

**Table 1:** Functional label distribution in the independent validation dataset.

Category		Positive samples
Stage-1	AMP	24,582
	Non-AMP	36,653
Stage-2	Anti-mammalian cells	895
	Antibacterial	14,543
	Antibiofilm	113
	Anticancer	2,559
	Antifungal	5,514
	Antigram-negative	2,478
	Antigram-positive	2,422
	Anti-HIV	468
	Anti-MRSA	329
	Antioxidant	69
	Antiparasitic	107
	Antiviral	2,142
	Cytotoxic	722
	Hemolytic	775

Number of AMP sequences annotated with functional activity in the independent validation dataset. Stage-1 denotes AMP identification, and Stage-2 shows the distribution of experimentally confirmed functional labels across 14 categories. The dataset was constructed to be nonredundant with the benchmark set and was derived from recent AMP databases.

### Overview of the MAPLE architecture

**Figure 1:**
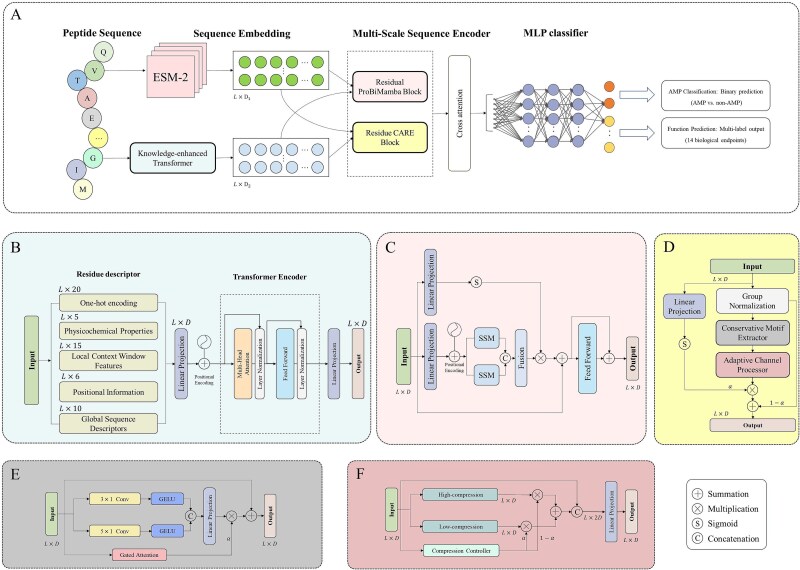
Overview of the MAPLE framework. (A) End-to-end framework of MAPLE. (B) Knowledge-enhanced transformer. (C) Residual ProBiMamba block. (D) Residue CARE block. (E) Conservative motif extractor. (F) Adaptive Channel processor.

MAPLE is an interpretable dual-stream framework designed to integrate evolutionary sequence representations with explicit physicochemical knowledge for AMP-related prediction tasks (Fig. 1). For each peptide, one stream uses ESM-2 to generate contextual residue-level embeddings that capture sequence-dependent evolutionary information [36], whereas the other stream encodes a 56-dimensional knowledge-based feature matrix composed of residue identity, physicochemical attributes, sliding-window statistics, positional indices, and global sequence descriptors (Supplementary Table S3). To introduce context awareness into the knowledge branch, these handcrafted features are processed by a dedicated four-layer transformer encoder [74].

To improve sensitivity to short functional motifs while limiting distortion of pretrained sequence semantics, we designed the CARE, inspired by structured local feature refinement strategies and normalization-based residual preservation [75–77]. CARE adaptively balances transformed local signals and the original input representation as follows:


$${\mathrm{X}}_{\mathrm{CARE}}=\mathrm{g}\odot{\mathrm{H}}_{\mathrm{CARE}}\left(\mathrm{X}\right)+\left(1-\mathrm{g}\right)\odot \mathrm{X},$$

where ${\mathrm{H}}_{\mathrm{CARE}}\left(\cdotp \right)$ denotes the composite local feature transformation and $\mathrm{g}$ is a learnable gate defined as $\mathrm{g}=\mathrm{\sigma} \left({\mathrm{W}}_{\mathrm{g}}\mathrm{X}+{\mathrm{b}}_{\mathrm{g}}\right)$.

In parallel, distributed sequence-level dependencies are modeled by ProBiMamba, a lightweight bidirectional Mamba-inspired sequence-mixing module motivated by recent developments in structured state-space sequence modeling [78–80]. The resulting local and global representations from both streams are integrated by cross-modal attention, followed by peptide-level prediction. Full mathematical formulations of the individual modules are provided in the Supplementary Methods.

### Model training, optimization, and model selection

AMP identification was treated as a binary classification task. Functional profiling was implemented as 14 label-specific binary classification tasks. Each classifier was trained separately using the same MAPLE architectural design and training protocol. To address class imbalance, we used a focal-loss-based objective for each binary prediction task [81]. For task t, let y ∈ {0,1} denote the ground-truth label and ${\mathrm{p}}_{\mathrm{t}}$ denote the predicted probability of the positive class. The probability assigned to the true class was defined as:


$${\mathrm{p}}_{\mathrm{y},\mathrm{t}}=\mathrm{y}\ {\mathrm{p}}_{\mathrm{t}}+\left(1-\mathrm{y}\right)\left(1-{\mathrm{p}}_{\mathrm{t}}\right)$$

The focal loss for task $\mathrm{t}$ was then computed as:


$$\mathrm{F}{\mathrm{L}}_{\mathrm{t}}=-{\mathrm{\alpha}}_{\mathrm{y},\mathrm{t}}{\left(1-{\mathrm{p}}_{\mathrm{y},\mathrm{t}}\right)}^{\mathrm{\gamma}}\log \left({\mathrm{p}}_{\mathrm{y},\mathrm{t}}\right)$$

where $\mathrm{\gamma}$ is the focusing parameter and ${\mathrm{\alpha}}_{\mathrm{y},\mathrm{t}}$ is the class-conditional balancing factor. In our implementation, $\mathrm{\gamma}$ was fixed at 2.0. The positive-class balancing factor was estimated from the class distribution of the benchmark training data as:


$${\mathrm{\alpha}}_{1,\mathrm{t}}=\mathrm{clip}\left(\frac{{\mathrm{N}}_{\mathrm{t}}^{-}}{{\mathrm{N}}_{\mathrm{t}}^{+}+{\mathrm{N}}_{\mathrm{t}}^{-}},\mathrm{0.05,0.95}\right)$$

and the negative-class balancing factor was defined as:


$${\mathrm{\alpha}}_{0,\mathrm{t}}=1-{\mathrm{\alpha}}_{1,\mathrm{t}}$$

Here, ${\mathrm{N}}_{\mathrm{t}}^{+}$ and ${\mathrm{N}}_{\mathrm{t}}^{-}$ denote the numbers of positive and negative samples, respectively, for task $\mathrm{t}$ in the benchmark training data. This formulation adaptively balances the relative contribution of positive and negative samples according to the task-specific class distribution.

All experiments were conducted on a Linux server equipped with dual Intel Xeon Silver 4210R processors, 376 GB RAM, and 4 NVIDIA GeForce RTX 3090 GPUs with 24 GB memory each. The software environment was based on Python 3.10.12, PyTorch 2.0.1, and CUDA 11.8. ESM-2 (esm2_t12_35M_UR50D) was used as the default sequence encoder [36]. Models were trained with AdamW using an initial learning rate of $1\times{10}^{-4}$ and a weight decay of $1\times{10}^{-5}$. Training was run for a maximum of 30 epochs.

Because the independent validation dataset was reserved for final evaluation, checkpoint monitoring was performed only within the benchmark model-development procedure. Checkpoints were saved after each epoch, and predicted probabilities were binarized at a fixed threshold of 0.5 during monitoring. The selected checkpoint was then carried forward for threshold estimation and final independent evaluation.

For functional activity prediction, task-specific decision thresholds were subsequently estimated on the benchmark dataset by maximizing F1:


$${\mathrm{\tau}}_{\mathrm{t}}=\arg \underset{\mathrm{\tau} \in \left(\mathrm{0.05,0.06},\dots, 0.95\right)}{\max}\mathrm{F}{1}_{\mathrm{t}}\left(\mathrm{\tau} \right),$$

and binary predictions were obtained as:


$${\hat{\mathrm{y}}}_{\mathrm{t}}=\mathbb{I}\left({\mathrm{p}}_{\mathrm{t}}\ge{\mathrm{\tau}}_{\mathrm{t}}\right)$$

To avoid evaluation leakage, all task-specific decision thresholds were determined before independent validation and were not reestimated using the independent validation dataset. The independent validation dataset was used solely for final evaluation. Overall functional performance was then summarized across the 14 independently trained classifiers using macro-averaged metrics.

### Performance evaluation metrics

We evaluated AMP identification using Accuracy, Precision, Sensitivity, Specificity, F1 score, Matthews correlation coefficient (MCC), area under the receiver operating characteristic curve (AUROC), and area under the precision–recall curve (AUPRC). For functional activity prediction, performance was assessed separately for each category-specific classifier and then summarized across the 14 independently trained classifiers using macro-averaged Precision, Sensitivity, F1 score, and AUROC. All metrics were reported for both the benchmark and independent validation datasets. Ninety-five percent confidence intervals were estimated using bootstrap resampling with 1000 iterations. Detailed metric definitions and calculation formulas are provided in the Supplementary Methods.

### K-mer enrichment and volcano plot analysis

To identify short sequence patterns associated with specific AMP functions, we performed one-versus-rest k-mer enrichment analysis on the benchmark dataset [82, 83]. For each target category, peptides annotated as positive for that activity were defined as the foreground set, whereas the remaining peptides were treated as the background set. All overlapping k-mers were extracted using a sliding window with step size 1. Motif lengths from 3 to 7 were initially compared, and 7-mers were selected for downstream analyses because they provided the best balance between motif resolution, enrichment strength, and cross-category specificity. To reduce noise from extremely sparse motifs, 7-mers occurring in fewer than five sequences across the combined foreground and background sets were excluded from downstream analysis. For each retained 7-mer k in task t, enrichment was quantified as follows:


$$\mathrm{Enrichment}\left(\mathrm{k},\mathrm{t}\right)={\log}_2\left(\frac{\left({\mathrm{a}}_{\mathrm{k},\mathrm{t}}+1\left)/\right({\mathrm{N}}_{\mathrm{t}}^{+}+2\right)}{\left({\mathrm{b}}_{\mathrm{k},\mathrm{t}}+1\left)/\right({\mathrm{N}}_{\mathrm{t}}^{-}+2\right)}\right)$$

where ${\mathrm{a}}_{\mathrm{k},\mathrm{t}}$ and ${\mathrm{b}}_{\mathrm{k},\mathrm{t}}$ denote the numbers of foreground and background sequences containing motif $\mathrm{k}$, respectively, and ${\mathrm{N}}_{\mathrm{t}}^{+}$ and ${\mathrm{N}}_{\mathrm{t}}^{-}$ denote the total numbers of sequences in the corresponding sets. A pseudocount of 1 was added to both motif counts to avoid undefined ratios when a motif was absent from either set. Statistical significance was assessed using Fisher’s exact test followed by false discovery rate correction [84, 85]. Volcano plots were used to visualize the results, with the *x*-axis indicating ${-\log}_{2}$ enrichment and the *y*-axis indicating ${-\log}_{10}$ (P value).

### Peptide design and virtual screening

To demonstrate a proof-of-concept design workflow, we first extracted antibacterial-selective sequence rules from the 7-mer enrichment analysis and the accompanying physicochemical profiling. Guided by these sequence and physicochemical constraints, we generated 100 000 virtual peptide sequences of 15–40 residues by biased sampling favoring cationic composition and controlled hydrophobicity. Novelty was enforced with respect to both the benchmark and independent validation datasets by excluding exact matches and near-duplicate sequences with Levenshtein distance ≤2.

The resulting virtual peptides were first screened using the MAPLE AMP-identification model. High-probability AMP candidates were then prioritized by jointly considering predictions from the antibacterial and hemolysis task-specific models, thereby enriching for candidates with favorable predicted selectivity. The final candidates were further characterized using external tools, including DBAASP [63], CamSol [86], AGGRESCAN [87], HemoPI-2 [88], and ToxinPred [89, 90], for independent cross-evaluation of physicochemical, toxicity, and developability-related properties. Detailed generation settings are provided in the Supplementary Methods.

## Results

### Comparative performance of AMP identification models

The performance of MAPLE for AMP identification was benchmarked against 11 representative state-of-the-art models, which were grouped into 3 methodological categories (Supplementary Table S4). The first category comprises traditional feature-engineered approaches, exemplified by AI4AMP [91], which rely on handcrafted physicochemical descriptors and classical machine learning classifiers. The second category includes end-to-end deep learning architectures that operate directly on raw sequences without pretraining, such as iAMP-CA2L [92], iAMPCN [54], AMPpred-MFA [56], BioPeptide-pred [55], and iAMP-Attenpred [39]. The third category encompasses models leveraging PLMs, including ProteinBERT [40], TransImbAMP [52] using TAPE-BERT [93], PepNet [41] built on ProtTrans [94], and UniDL4BioPep [53] and PLAPD [37] derived from ESM-2 [36]. These baselines span multiple backbone families and parameter scales, thereby providing cross-backbone context for interpreting MAPLE’s comparative performance. All baseline models were evaluated using either publicly available implementations or rigorous re-implementations based on the original descriptions to support a consistent comparison.

**Figure 2:**
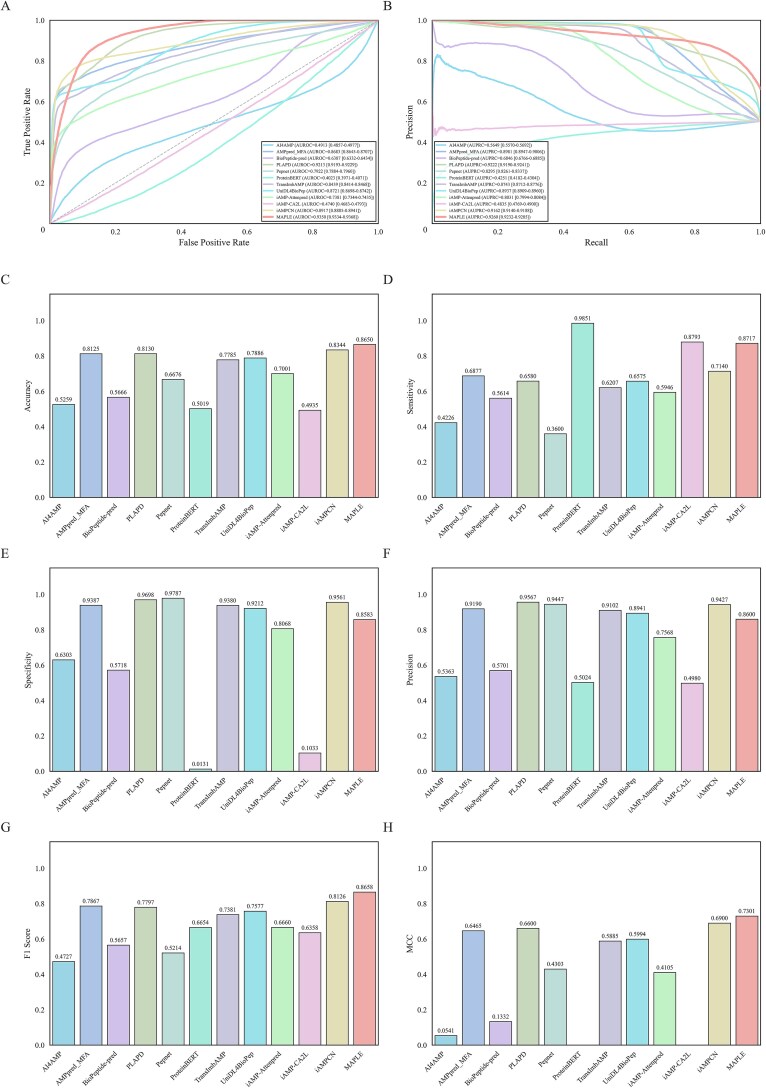
Comprehensive evaluation of AMP identification models on the independent validation dataset. Performance comparisons between MAPLE and state-of-the-art predictors are illustrated through (A) ROC curves and (B) PR curves, alongside a quantitative evaluation based on (C) accuracy, (D) sensitivity, (E) specificity, (F) precision, (G) F1 score, and (H) MCC.

On the benchmark dataset, MAPLE achieved very high discrimination (Supplementary Fig. S6, Supplementary Table S5). However, benchmark performance may overestimate performance on non-overlapping sequence sets owing to overfitting or dataset-specific biases [95, 96]. This overestimation becomes evident in our comparative evaluation: although most competing models achieved strong benchmark performance, their accuracy declined substantially on the independent validation sequences. In contrast, MAPLE maintained robust performance under this stringent setting, achieving the highest AUROC (0.9350) and AUPRC (0.9260) on the independent validation dataset, outperforming leading baselines (Fig. 2A and B; Supplementary Table S5). MAPLE also showed the best threshold-dependent overall performance on the independent validation set, as reflected by accuracy, balanced accuracy, and MCC (Fig. 2C–H; Supplementary Table S6). These findings support MAPLE’s balanced sequence-level generalization to previously unseen, non-overlapping sequences.

### Comparative performance of AMP function prediction models

Accurate prediction of specific biological functions is considerably more challenging than binary AMP identification because of pronounced heterogeneity in label prevalence, high functional diversity, and overlapping mechanisms across categories. We systematically compared MAPLE with six representative AMP predictors covering multiple functional activity endpoints that provide publicly available code, including BioPeptide-pred [55], UniDL4BioPep [53], TransImbAMP [52], iAMPCN [54], iAMP-CA2L [92], and deep-AMPpred [38]. Additionally, because hemolytic activity is a critical safety concern, we included two dedicated hemolysis prediction models: HemoFuse [97] and HemoPI-2 [88] (Supplementary Table S4). On the benchmark dataset, several existing models showed reasonable macro-averaged performance (Supplementary Table S7). However, under the independent validation setting, MAPLE demonstrated more robust generalization despite severe label imbalance, achieving a macro-AUROC of 0.8477 (95% CI: 0.8441–0.8529) and a macro-F1 of 0.5141 (95% CI: 0.5026–0.5233) (Table 2).

**Table 2:** Comparison of macro-averaged performance metrics for different AMP function prediction models on independent validation dataset.

Model	Macro-F1	Macro-precision	Macro-sensitivity	Macro-AUROC
iAMPCN	0.2085 (0.2029, 0.2145)	**0.6006 (0.5499–0.6367)**	0.1487 (0.1447–0.1532)	0.7757 (0.7690–0.7855)
TransImbAMP	0.1826 (0.1761–0.1887)	0.4089 (0.2468–0.5316)	0.2676 (0.2613–0.2757)	0.6370 (0.6280–0.6464)
deep_AMPpred	0.1466 (0.1422–0.1505)	0.2853 (0.2747–0.2953)	0.1669 (0.1632–0.1713)	0.6057 (0.5972–0.6128)
iAMP-CA2L	0.0602 (0.0567–0.0634)	0.1876 (0.1505–0.2269)	0.0534 (0.0514–0.0555)	0.5052 (0.4959–0.5140)
BioPeptide-pred	0.2374 (0.2334–0.2412)	0.1863 (0.1829–0.1900)	0.4744 (0.4631–0.4856)	0.5131 (0.5050–0.5237)
UniDL4BioPep	0.2141 (0.2113–0.2159)	0.1422 (0.1402–0.1435)	**0.6664 (0.6563–0.6798)**	0.5753 (0.5696–0.5805)
MAPLE	**0.5141 (0.5026–0.5233)**	0.4787 (0.4670–0.4913)	0.6113 (0.6012–0.6215)	**0.8477 (0.8441–0.8529)**

Bold values indicate the best performance for each metric.

**Figure 3:**
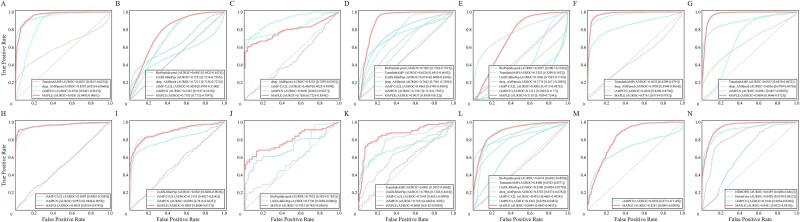
Comparison of ROC curves for different models on the independent validation dataset. Functional categories include (A) anti-mammalian cells, (B) antibacterial, (C) antibiofilm, (D) anticancer, (E) antifungal, (F) antigram-negative, (G) antigram-positive, (H) anti-HIV, (I) anti-MRSA, (J) antioxidant, (K) antiparasitic, (L) antiviral, (M) cytotoxic, and (N) hemolytic.

**Figure 4:**
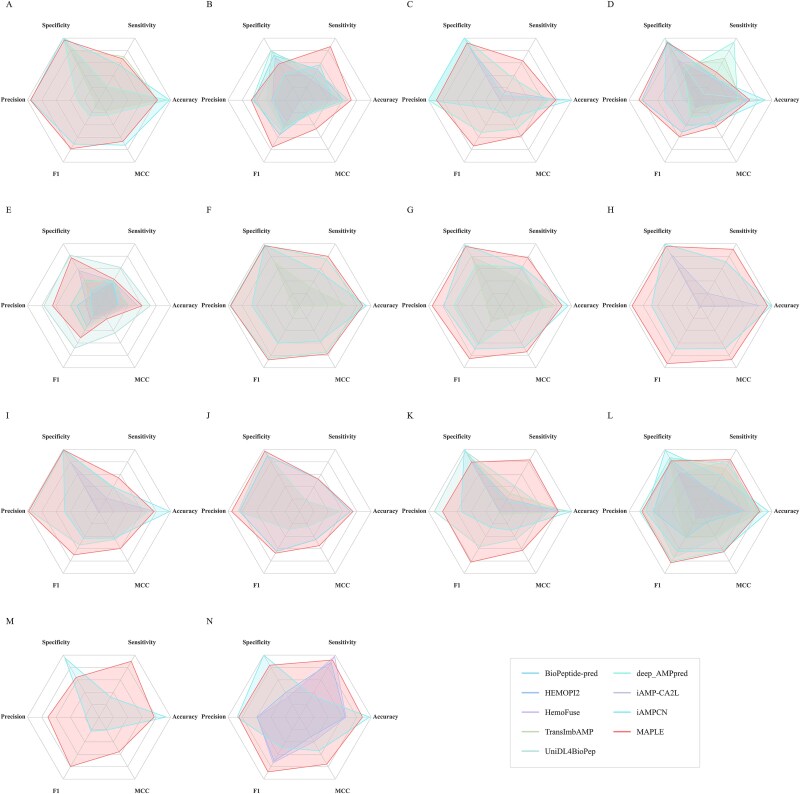
Performance of different models across 14 functional labels on the independent validation dataset. Subfigures correspond to the following prediction tasks: (A) anti-mammalian cells, (B) antibacterial, (C) antibiofilm, (D) anticancer, (E) antifungal, (F) antigram-negative, (G) antigram-positive, (H) anti-HIV, (I) anti-MRSA, (J) antioxidant, (K) antiparasitic, (L) antiviral, (M) cytotoxic, and (N) hemolytic. Metrics evaluated include accuracy, precision, sensitivity, specificity, F1 score, and MCC.

Detailed per-label results on the benchmark dataset are provided in Supplementary Table S8, and independent validation curves are shown in Fig. 3A–N. On the independent validation set, MAPLE retained strong discrimination across major antibacterial endpoints as well as safety-related phenotypes, including hemolytic activity, mammalian cell activity, and general cytotoxicity. These results indicate that MAPLE supports coordinated evaluation of antimicrobial efficacy and mammalian toxicity signals at the sequence level under a shared architectural framework. The radar charts (Fig. 4) summarize threshold-dependent performance across the 14 functional categories. Compared with baseline models, MAPLE showed a more even threshold-dependent performance profile across functional categories, particularly for low-prevalence endpoints. Such balanced threshold-dependent performance is essential for downstream prioritization, and we further demonstrated MAPLE’s practical utility by jointly stratifying hemolysis risk among peptides predicted as antibacterial (Supplementary Fig. S7).

### Ablation and backbone comparison analyses

Ablation analyses were conducted on the benchmark dataset under the same training and evaluation protocol as the corresponding main experiments to dissect the contributions of MAPLE’s architectural and optimization components (Tables 3 and 4; Supplementary Tables S9 and S10). Across both tasks, the complete MAPLE model consistently delivered the strongest and most balanced performance, indicating that the observed gains arise from dual-stream fusion and local–global sequence modeling, rather than from any single modality alone.

**Table 3:** Ablation study of MAPLE model components for the AMP discrimination on benchmark dataset.

ID	Architecture description	AUROC	Accuracy	Precision	Sensitivity	Specificity	F1 score	MCC
ESM-2 stream optimization								
A-1	Baseline (ESM-2 Only)	0.9340 (0.9321–0.9359)	0.9134 (0.9117–0.9152)	0.9724 (0.9700–0.9750)	0.6713 (0.6653–0.6776)	0.9937 (0.9931–0.9942)	0.7943 (0.7902–0.7984)	0.7622 (0.7578–0.7662)
A-2	+ CARE (Local motifs)	0.9634 (0.9620–0.9647)	0.9431 (0.9417–0.9445)	0.9810 (0.9790–0.9829)	0.7869 (0.7820–0.7921)	0.9949 (0.9944–0.9954)	0.8733 (0.8700–0.8764)	0.8457 (0.8418–0.8495)
A-3	+ ProBiMamba (Global dependency)	0.9909 (0.9902–0.9917)	0.9790 (0.9781–0.9800)	0.9729 (0.9708–0.9750)	0.9421 (0.9389–0.9450)	0.9913 (0.9906–0.9920)	0.9573 (0.9555–0.9591)	0.9436 (0.9411–0.9461)
A-4	+ CARE and ProBiMamba (Parallel)	0.9978 (0.9976–0.9980)	0.9829 (0.9820–0.9837)	0.9870 (0.9855–0.9884)	0.9436 (0.9408–0.9465)	0.9959 (0.9954–0.9963)	0.9648 (0.9632–0.9664)	0.9540 (0.9517–0.9560)
Knowledge stream optimization								
B-1	Baseline (Knowledge Only)	0.8806 (0.8769–0.8847)	0.9239 (0.9222–0.9256)	0.9587 (0.9557–0.9615)	0.7257 (0.7204–0.7312)	0.9896 (0.9889–0.9904)	0.8260 (0.8220–0.8296)	0.7908 (0.7868–0.7950)
B-2	+ CARE (Local motifs)	0.9155 (0.9124–0.9187)	0.9478 (0.9465–0.9492)	0.9680 (0.9655–0.9705)	0.8175 (0.8125–0.8225)	0.9910 (0.9903–0.9917)	0.8863 (0.8833–0.8894)	0.8580 (0.8544–0.8616)
B-3	+ ProBiMamba (Global dependency)	0.9004 (0.8971–0.9039)	0.9429 (0.9413–0.9443)	0.9721 (0.9698–0.9742)	0.7932 (0.7875–0.7982)	0.9925 (0.9918–0.9931)	0.8736 (0.8702–0.8769)	0.8444 (0.8408–0.8481)
B-4	+ CARE and ProBiMamba (Parallel)	0.9691 (0.9674–0.9707)	0.9605 (0.9593–0.9617)	0.9721 (0.9700–0.9743)	0.8664 (0.8618–0.8706)	0.9917 (0.9911–0.9924)	0.9162 (0.9136–0.9190)	0.8930 (0.8897–0.8962)
Dual-stream fusion strategy								
C-1	Dual Baseline (No Modules)	0.9963 (0.9961–0.9966)	0.9751 (0.9741–0.9761)	0.9781 (0.9761–0.9800)	0.9206 (0.9169–0.9238)	0.9931 (0.9926–0.9937)	0.9484 (0.9462–0.9506)	0.9328 (0.9302–0.9354)
C-2	Dual + CARE Only	0.9972 (0.9971–0.9974)	0.9783 (0.9774–0.9793)	0.9828 (0.9811–0.9844)	0.9292 (0.9261–0.9323)	0.9946 (0.9941–0.9951)	0.9553 (0.9534–0.9572)	0.9416 (0.9392–0.9440)
C-3	Dual + ProBiMamba Only	0.9952 (0.9948–0.9956)	0.9798 (0.9789–0.9807)	0.9848 (0.9832–0.9864)	0.9335 (0.9305–0.9366)	0.9952 (0.9948–0.9957)	0.9585 (0.9567–0.9603)	0.9458 (0.9436–0.9481)
Proposed framework								
D (MAPLE)	Dual + CARE + ProBiMamba	**0.9989 (0.9988–0.9990)**	**0.9851 (0.9843–0.9858)**	**0.9845 (0.9830–0.9860)**	**0.9577 (0.9552–0.9602)**	**0.9947 (0.9942–0.9952)**	**0.9709 (0.9694–0.9724)**	**0.9611 (0.9590–0.9629)**

Bold values indicate the best performance for each metric.

**Table 4:** Ablation study of MAPLE model components for the AMP multi-label functional prediction on benchmark dataset.

ID	Architecture description	Macro-F1	Macro-precision	Macro-sensitivity	Macro-AUROC
**ESM-2 stream optimization**					
**A-1**	Baseline (ESM-2 Only)	0.4796 (0.4741–0.4844)	0.3655 (0.3605–0.3697)	0.8023 (0.7945–0.8099)	0.8853 (0.8832–0.8875)
**A-2**	+ CARE (Local motifs)	0.5418 (0.5354–0.5477)	0.4267 (0.4209–0.4320)	0.7808 (0.7746–0.7879)	0.9002 (0.8981–0.9026)
**A-3**	+ ProBiMamba (Global dependency)	0.5704 (0.5643–0.5767)	0.4565 (0.4514–0.4624)	0.8681 (0.8639–0.8727)	0.9287 (0.9272–0.9302)
**A-4**	+ CARE and ProBiMamba (Parallel)	0.7286 (0.7210–0.7354)	0.6113 (0.6020–0.6187)	0.9225 (0.9195–0.9259)	0.9642 (0.9632–0.9651)
**Knowledge stream optimization**					
**B-1**	Baseline (Knowledge Only)	0.3567 (0.3526–0.3612)	0.2813 (0.2763–0.2858)	0.5729 (0.5676–0.5774)	0.7390 (0.7343–0.7439)
**B-2**	+ CARE (Local motifs)	0.3847 (0.3790–0.3908)	0.3367 (0.3203–0.3503)	0.5316 (0.5249–0.5380)	0.7475 (0.7426–0.7525)
**B-3**	+ ProBiMamba (Global dependency)	0.3957 (0.3922–0.3996)	0.3118 (0.3085–0.3152)	0.6573 (0.6480–0.6642)	0.7951 (0.7909–0.7991)
**B-4**	+ CARE and ProBiMamba (Parallel)	0.4919 (0.4864–0.4989)	0.3770 (0.3721–0.3836)	0.7435 (0.7362–0.7505)	0.8699 (0.8677–0.8721)
**Dual-stream fusion strategy**					
**C-1**	Dual Baseline (No Modules)	0.6549 (0.6474–0.6616)	0.5452 (0.5361–0.5528)	0.8571 (0.8516–0.8613)	0.9399 (0.9385–0.9411)
**C-2**	Dual + CARE Only	0.6869 (0.6801–0.6933)	0.5698 (0.5621–0.5774)	0.8973 (0.8939–0.9013)	0.9534 (0.9522–0.9543)
**C-3**	Dual + ProBiMamba Only	0.7087 (0.7027–0.7138)	0.5794 (0.5724–0.5856)	0.9365 (0.9329–0.9400)	0.9619 (0.9608–0.9628)
**Proposed framework**					
**D (MAPLE)**	Dual + CARE + ProBiMamba	**0.7644 (0.7580–0.7701)**	**0.6552 (0.6468–0.6622)**	**0.9365 (0.9338–0.9390)**	**0.9721 (0.9714–0.9729)**

Bold values indicate the best performance for each metric.

For AMP discrimination, both single-stream variants showed weaker performance than the full model, supporting complementary contributions from the ESM-2 and knowledge branches. Adding CARE and ProBiMamba further improved the dual-stream backbone, indicating that the performance gains were not solely explained by feature fusion. Loss-function ablations additionally showed that the imbalance-aware objective contributed to more balanced threshold-dependent performance (Table 3; Supplementary Table S9).

Single-stream variants were consistently inferior to the full model, supporting the importance of integrating dual-source information for complex functional annotation. Starting from the dual-stream backbone, adding CARE and ProBiMamba provided further improvements, with the full model achieving the best macro-level performance overall. Loss-function ablations likewise supported the contribution of the chosen objective to improved macro-level and threshold-dependent performance under label imbalance (Table 4; Supplementary Table S10).

Beyond component-level ablations, we further assessed encoder robustness by replacing the default 35M ESM-2 encoder with representative alternatives while keeping the remaining architecture, training strategy, and evaluation protocol unchanged (Table 5). Within the ESM-2 family, performance improved from 8M to 35M, but no additional gains were observed for 150M or 650M, indicating that simply enlarging the pretrained encoder does not further improve MAPLE under the current benchmark setting. ESM-C-300M yielded highly comparable results, whereas ProtT5-XL-UniRef50 and ProteinBERT showed somewhat larger declines, particularly for functional prediction. Overall, these results identify the default 35M ESM-2 encoder as the best-performing backbone among those tested, while also showing that simply increasing pretrained encoder scale does not necessarily improve MAPLE.

**Table 5:** Robustness of MAPLE across representative protein language model backbones on the benchmark dataset.

PLM backbone	Emb. dim.	AMP AUROC	AMP F1	Func. Macro-F1	Func. Macro-AUROC
esm2_t6_8M_UR50D	320	0.9983 (0.9981–0.9985)	0.9674 (0.9655–0.9693)	0.7548 (0.7480–0.7615)	0.9691 (0.9681–0.9701)
esm2_t12_35M_UR50D	480	0.9989 (0.9988–0.9990)	0.9709 (0.9694–0.9724)	0.7644 (0.7580–0.7701)	0.9721 (0.9714–0.9729)
esm2_t30_150M_UR50D	640	0.9988 (0.9987–0.9989)	0.9703 (0.9688–0.9718)	0.7629 (0.7566–0.7690)	0.9717 (0.9709–0.9725)
esm2_t33_650M_UR50D	1280	0.9987 (0.9986–0.9988)	0.9701 (0.9689–0.9717)	0.7618 (0.7552–0.7683)	0.9713 (0.9705–0.9721)
ESM-C-300 M	960	0.9985 (0.9983–0.9987)	0.9706 (0.9688–0.9722)	0.7624 (0.7557–0.7690)	0.9716 (0.9707–0.9725)
ProtT5-XL-UniRef50	1024	0.9979 (0.9976–0.9982)	0.9651 (0.9631–0.9672)	0.7486 (0.7414–0.7559)	0.9664 (0.9653–0.9675)
ProteinBERT (local)	128	0.9964 (0.9960–0.9968)	0.9590 (0.9566–0.9615)	0.7321 (0.7244–0.7398)	0.9617 (0.9605–0.9630)

Note: PLM, protein language model; Emb. dim., embedding dimension; AMP, antimicrobial peptide identification; Func., multi-label functional prediction; AUROC, area under the receiver operating characteristic curve; F1, F1 score; CI, confidence interval. Values are reported as mean (95% CI) estimated by bootstrap resampling with 1000 iterations. For ProteinBERT, the reported embedding dimension corresponds to the local per-residue representation used for fair sequence-embedding substitution.

### Model interpretability

**Figure 5:**
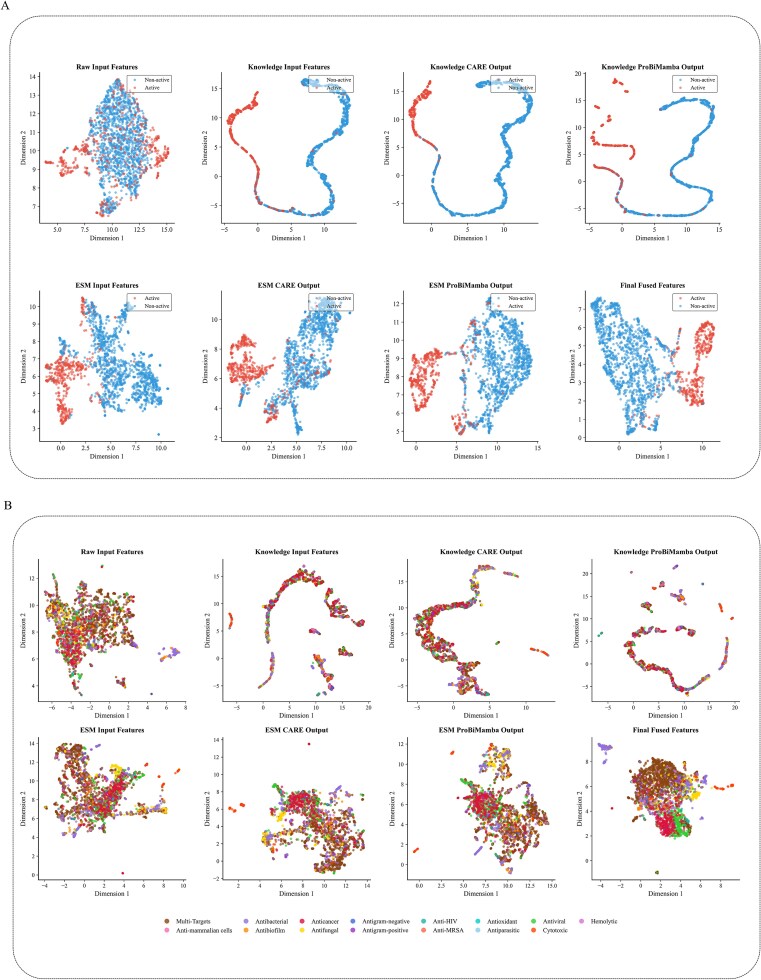
UMAP visualization of intermediate and final representations in MAPLE. (A) AMP identification; (B) functional annotation.

To visualize how peptide representations evolve across the MAPLE pipeline, we applied uniform manifold approximation and projection (UMAP) to sequence-level embeddings extracted from successive representation stages [98]. As shown in Fig. 5A, the embedding distribution evolved from a relatively scattered initial state to increasingly well-separated clusters across representation stages. In the final fused space, AMP and non-AMP samples formed two clearly separated groups, consistent with improved class organization after multimodal integration.

When colored by functional annotations, the embedding space exhibited heterogeneous label organization across the 14 categories (Fig. 5B). Early-stage representations are broadly intermixed, whereas label-dependent enrichment becomes more apparent in the fused space. Peptides carrying multiple activity labels, together with subsets of antiviral, anticancer, antifungal, and cytotoxic sequences, showed localized clustering in the fused space, whereas low-prevalence or mechanistically diverse labels remained diffuse, consistent with co-annotation and limited sample sizes.

Focusing on the antibacterial–hemolytic axis, we quantified the separation in the fused space by inter-centroid distance and kernel density overlap (Supplementary Fig. S8). The centroid distance was 0.4936 (95% CI: 0.3797–0.6470), and a label-permutation test confirmed its significance (*P* < .001). These results motivated subsequent motif-level analyses to determine whether the contrast reflects distinct, engineerable determinants, or distributed differences within a shared-sequence manifold.

### k-mer analysis

**Figure 6:**
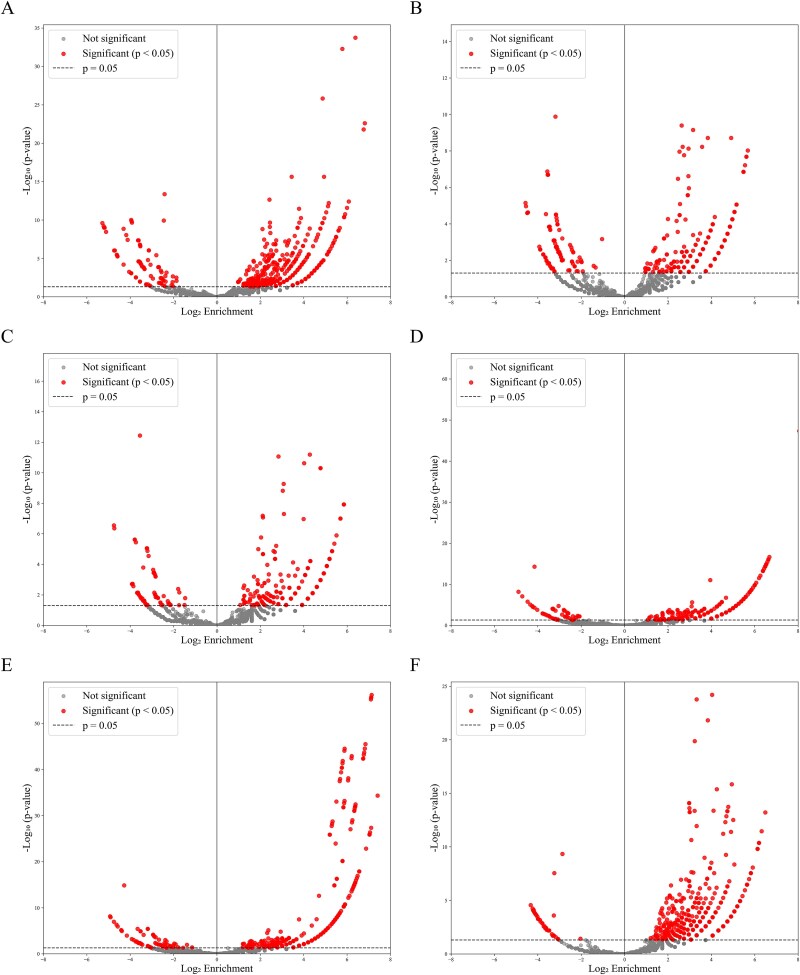
The 7-mer enrichment landscapes across representative functions. Volcano plots summarize one-versus-rest 7-mer enrichment results for each endpoint against the benchmark background. Each point represents one 7-mer. The *x*-axis shows ${\log}_2$ enrichment, and the *y*-axis shows ${-\log}_{10}$(P value). Thus, points in the upper-right region indicate motifs that are significantly enriched in the target function. Vertical and horizontal dashed lines indicate the fold-enrichment and significance thresholds used to define enriched motifs, respectively. Panels show (A) antibacterial, (B) antifungal, (C) anticancer, (D) antiviral, (E) anti-mammalian cells, and (F) hemolytic.

We performed a one-versus-rest k-mer enrichment analysis across 14 functional categories on the benchmark dataset, using volcano plots to visualize both enrichment magnitude and statistical significance. Among motif lengths from 3 to 7, 7-mers were selected for downstream analyses because they provided the best balance between motif resolution, enrichment strength, and cross-category specificity (Supplementary Fig. S9). Volcano plots summarizing representative therapeutic activities (antibacterial, antifungal, anticancer, and antiviral) and toxicity endpoints (anti-mammalian cells and hemolytic) revealed distinct enrichment regimes (Fig. 6). Therapeutic categories differed markedly in enrichment breadth; antibacterial peptides showed a broad distribution of moderately enriched motifs, antifungal activity was driven by fewer sharply enriched motifs, anticancer activity displayed a dispersed landscape with high fold-change outliers, and antiviral activity exhibited a relatively constrained set of enriched motifs. Toxicity landscapes overlapped with therapeutic landscapes, emphasizing the difficulty of sequence-level selectivity. In particular, hemolysis showed substantial overlap with antibacterial enrichment (Supplementary Fig. S10), consistent with the shared requirements for amphipathic membrane disruption. However, the most strongly hemolysis-enriched motifs were often less antibacterial-enriched, suggesting that severe hemolysis may involve additional sequence features beyond those sufficient for bacterial killing. The remaining functional categories are summarized in Supplementary Fig. S11.

**Figure 7:**
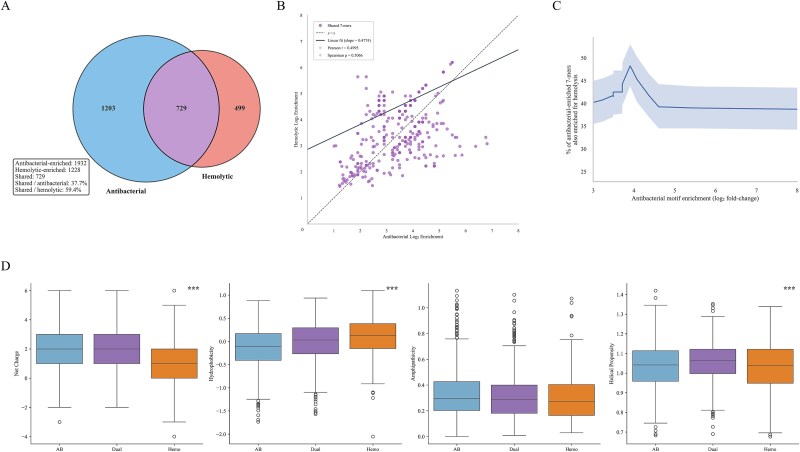
Motif enrichment reveals separable selectivity in efficacy–toxicity space. (A) Asymmetric overlap of significantly enriched 7-mer motifs between antibacterial and hemolytic categories. (B) Enrichment strengths of shared (dual-activity) 7-mers, showing a moderate positive association with dispersion. (C) Antibacterial-enrichment-dependent hemolytic overlap of antibacterial-enriched 7-mers, with uncertainty indicated by the shaded band. (D) Physicochemical comparison of motif groups, including net charge, hydrophobicity, amphipathicity, and α-helical propensity.

Focusing on the antibacterial–hemolytic axis, 7-mer enrichment indicated partially overlapping, yet distinguishable motif repertoires (Fig. 7A). Motif sharing was asymmetric; many hemolysis-enriched motifs overlapped with antibacterial motifs, whereas most antibacterial-enriched motifs were not hemolytic. Among the shared motifs, antibacterial and hemolytic enrichment strengths showed a moderate positive association with substantial dispersion (Fig. 7B). A sliding-window analysis across the antibacterial enrichment axis further revealed a non-monotonic pattern; hemolytic overlap peaked at intermediate antibacterial enrichment and decreased for the most strongly antibacterial-enriched motifs (Fig. 7C). Physicochemical profiling explained this divergence (Fig. 7D); antibacterial-selective motifs occupied a regime of moderate cationicity, lower hydrophobicity, and higher amphipathicity, whereas hemolysis-selective motifs were more hydrophobic and less cationic. Sequence logo analysis supported these trends at the residue level (Supplementary Fig. S12).

### Peptide design and virtual screening

**Figure 8:**
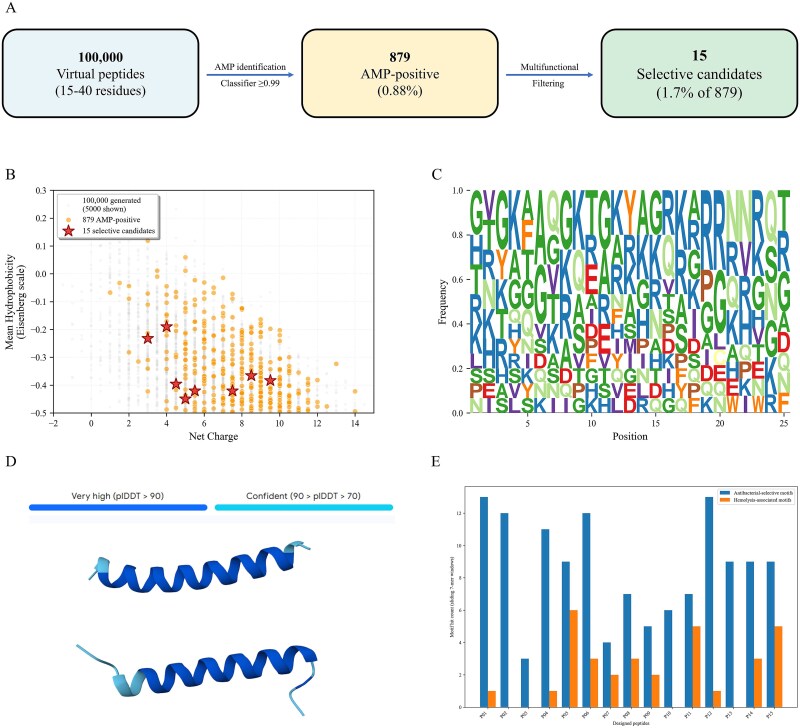
Proof-of-concept peptide design and virtual screening guided by interpretable k-mer analysis. (A) Workflow for motif-guided virtual peptide generation under built-in novelty constraints, followed by MAPLE-based AMP identification, antibacterial–hemolysis prioritization, and final candidate selection. (B) Distribution of generated peptides, AMP-positive peptides, and the 15 prioritized candidates in the net charge–mean hydrophobicity space, showing that the selected candidates occupy a positively charged yet hydrophobicity-controlled region. (C) Sequence logo of the 15 prioritized candidates, highlighting recurrent lysine/arginine enrichment and controlled hydrophobic patterning consistent with antibacterial-selective motifs. (D) Representative AlphaFold2 models are shown as structural plausibility analyses to assess compatibility with AMP-like amphipathic α-helical organization. (E) Motif-matching analysis showing that the prioritized candidates retain more antibacterial-selective 7-mer patterns than hemolysis-associated motifs.

To translate the k-mer-derived selectivity rules into candidate design, we generated a 100 000-sequence virtual library using motif-guided amino acid biases that favored moderate cationicity and reduced hydrophobicity, while imposing built-in novelty constraints relative to the benchmark and independent validation datasets. Screening with MAPLE first identified 879 high-probability AMP candidates, and subsequent antibacterial–hemolysis prioritization further selected 15 peptides with high predicted antibacterial activity and low predicted hemolysis (Fig. 8A; Supplementary Table S11).

In the charge–hydrophobicity space, these prioritized candidates occupied a region characterized by positive net charge together with controlled, generally lower mean hydrophobicity relative to the broader AMP-positive pool (Fig. 8B), matching the physicochemical regime associated with antibacterial-selective motifs. This agreement was also evident at the residue level (Fig. 8C): the candidate peptides showed recurrent enrichment of lysine and arginine across multiple positions, consistent with the cationic signature of antibacterial-selective motifs, whereas hydrophobic residues were distributed in a more controlled and discontinuous manner rather than forming extended uninterrupted stretches more characteristic of hemolysis-associated patterns. AlphaFold2 predictions further suggested that representative candidates remained compatible with AMP-like amphipathic α-helical organization [99] (Fig. 8D), providing a structural plausibility check of the motif-guided design strategy. Motif-matching analysis further reinforced this interpretation, showing that the prioritized candidates consistently retained more antibacterial-selective 7-mer patterns than hemolysis-associated motifs (Fig. 8E). Additional physicochemical descriptors, conformational properties, developability indicators, and external platform-based cross-evaluations are summarized in Supplementary Tables S12–S15.

## Conclusion

The antimicrobial resistance crisis has renewed interest in AMPs, yet translation is often framed by an assumed potency–toxicity trade-off. Here, we present MAPLE, a dual-stream framework for AMP identification and systematic category-specific functional profiling across 14 activity categories. By integrating pretrained evolutionary embeddings with explicit physicochemical features in a dual-stream architecture, MAPLE achieves robust generalization and balanced performance under severe label imbalance. Beyond prediction, systematic 7-mer enrichment provides mechanistically interpretable hypotheses about sequence selectivity: antibacterial and hemolytic activities exhibit partially overlapping yet distinguishable motif repertoires, and hemolytic overlap varies non-monotonically with antibacterial motif enrichment, challenging the assumption that stronger antibacterial motif enrichment necessarily implies greater hemolytic overlap. Physicochemical profiling further indicated that antibacterial-selective motifs occupied a regime of moderate cationicity, lower hydrophobicity, and higher amphipathicity, which is consistent with physicochemical features expected for bacteria-selective membrane interactions. A proof-of-concept design workflow guided by motif-derived compositional biases prioritized 15 candidate peptides with favorable predicted selectivity and a consistent motif composition. Although experimental validation and structural integration remain important, MAPLE offers an interpretable and scalable approach for deriving actionable design principles for selective AMPs.

**Key Points**

- MAPLE is an interpretable dual-stream framework for AMP identification and category-specific functional profiling across 14 AMP-related activity categories, enabling coordinated efficacy and toxicity assessment at the sequence level.
- The model delivers balanced performance under extreme class imbalance, improving robustness for low-prevalence but clinically relevant activities during independent evaluations.
- Motif-level enrichment analyses challenge the assumed efficacy–toxicity trade-off, showing that highly antibacterial-enriched motifs exhibit reduced hemolytic overlap compared to moderately enriched motifs.
- Antibacterial-selective motifs occupy a distinct physicochemical regime—moderate cationicity, lower hydrophobicity, and higher amphipathicity—consistent with bacteria-selective membrane interactions.
- The proof-of-concept motif-guided design prioritized 15 candidate peptides with favorable predicted selectivity and AlphaFold-based structural plausibility suggesting compatibility with amphipathic α-helical conformations.

## Supplementary Material

Supporting_Information_bbag318

## Data Availability

The benchmark dataset was constructed based on the iAMPCN dataset and was further refined through cross-database label harmonization using nine publicly available AMP resources: dbAMP v3.0 (http://csb.cse.yzu.edu.tw/dbAMP/), DRAMP v4.0 (http://dramp.cpu-bioinfor.org/), CAMPR4 (http://www.camp.bicnirrh.res.in/), APD3 (https://aps.unmc.edu/), AntiFP (https://antifungipept.chemoinfolab.com), DBAASP v3.0 (https://dbaasp.org/), AMPdb V1 (https://bblserver.org.in/ampdb/), DCTPep (http://dctpep.cpu-bioinfor.org/), and DRAVP (https://dravp.cpu-bioinfor.org/). The final benchmark dataset comprised 25 507 AMP sequences and 72 606 non-AMP sequences. The independent validation dataset comprised 24 582 AMP sequences and 36 653 non-AMP sequences. AMP sequences in the independent validation dataset were collected from the same nine publicly available resources, whereas non-AMP sequences were obtained from UniProt (https://www.uniprot.org/). Sequence overlap between the benchmark dataset and the independent validation dataset was removed to ensure sequence-level non-redundancy.

## Code availability

The source code for MAPLE is publicly available at https://github.com/Harkool/MAPLE. The repository provides model implementation, preprocessing scripts, feature-construction scripts, inference code, example input files, demo outputs, trained model weights, task-specific thresholds, and a Streamlit-based local prediction interface.
