# Supplementary material for: MAPLE: interpretable deep learning identifies selective antimicrobial peptides using joint evolutionary–physicochemical analysis
Source: Brief Bioinform. 2026 Jun 17;27(3):bbag318. doi: 10.1093/bib/bbag318 (PMC13274993; doi:10.1093/bib/bbag318)
Supplement: Supporting_Information_bbag318 [file supporting_information_bbag318.docx]

**Supporting Information**

**Contents**

[**Supplementary Methods** 3](#_Toc228376093)

[S1. Dataset construction and annotation 3](#_Toc228376094)

[S1.1 Integration of AMP databases 3](#_Toc228376095)

[S1.2 Data quality control and cleaning pipeline 3](#_Toc228376096)

[S1.3 Benchmark dataset construction strategy 4](#_Toc228376097)

[S1.4 Redundancy Reduction Using CD-HIT 4](#_Toc228376098)

[S1.5 Negative Sample Selection Strategy 5](#_Toc228376099)

[S2. Feature representation and preprocessing 5](#_Toc228376100)

[S2.1 ESM-2 Embeddings 6](#_Toc228376101)

[S2.2 Knowledge-Based Feature Construction 6](#_Toc228376102)

[S2.3 Knowledge Feature Enhancement via Transformer 7](#_Toc228376103)

[S3. Model Architecture Details 8](#_Toc228376104)

[S3.1 Overall Architecture Overview 8](#_Toc228376105)

[S3.2 Input Projection and Dimension Alignment 9](#_Toc228376106)

[S3.3 CARE Module: Implementation and Configuration Details 10](#_Toc228376107)

[S3.4 ProBiMamba Module: Bidirectional Mamba-inspired Sequence Mixer for Peptides 12](#_Toc228376108)

[S3.5 Cross-Modal Fusion: Four-Level Integration Strategy 13](#_Toc228376109)

[S3.6 Classification Head and Output Layer 15](#_Toc228376110)

[S4. Evaluation and uncertainty quantification 16](#_Toc228376111)

[S4.1 Evaluation metrics for AMP discrimination 16](#_Toc228376112)

[S4.2 Evaluation and uncertainty quantification 17](#_Toc228376113)

[S5. Interpretability and motif analyses 18](#_Toc228376114)

[S5.1 UMAP visualization of peptide embedding space 18](#_Toc228376115)

[S5.2 K-mer enrichment analysis protocol 18](#_Toc228376116)

[S5.3 Rationale for motif-length selection and downstream comparative analyses 19](#_Toc228376117)

[S5.4 Virtual peptide library generation and novelty filtering 19](#_Toc228376118)

[**Supplementary Results** 21](#_Toc228376119)

[R1. Sequence characterization 21](#_Toc228376120)

[R1.1 Benchmark dataset characterization 21](#_Toc228376121)

[R1.2 Independent validation dataset characterization 22](#_Toc228376122)

[R2 Model Comparison and Evolution 23](#_Toc228376123)

[R2.1 Models Methodological Comparison and Evolution of AMP Prediction Models 23](#_Toc228376124)

[R2.2 Selectivity assessment via dual prediction 23](#_Toc228376125)

[R2.3 Embedding space visualization 24](#_Toc228376126)

[R3. Motif-level findings 24](#_Toc228376127)

[R3.1 K-mer length optimization 24](#_Toc228376128)

[R3.2 Antibacterial vs hemolytic overlap 25](#_Toc228376129)

[R3.3 Other functions enrichment maps 26](#_Toc228376130)

[R3.4 Motif logos & categories 27](#_Toc228376131)

[R3.5 Motif-guided candidate prioritization 28](#_Toc228376132)

[**Supplementary Tables** 30](#_Toc228376133)

[**Supplementary Figures** 69](#_Toc228376134)

[**References** 81](#_Toc228376135)

**Supplementary Methods**

### S1. Dataset construction and annotation

### S1.1 Integration of AMP databases

The comprehensive dataset construction involved systematic curation from nine specialized antimicrobial peptide (AMP) databases: dbAMP v3.0(1), DRAMP v4.0(2), CAMPR4(3), APD3(4), AntiFP(5), DBAASP v3.0(6), AMPdb(7), DCTPep(8) and DRAVP(9). These databases were selected based on their relevance, update frequency, and the availability of experimentally validated annotations. Together, they provide distinct and complementary coverage of AMP-related functional activities across 14 predefined categories, including antibacterial, anticancer, antiviral, antifungal, and hemolytic functions (Supplementary Table S1).

To unify functional annotations across these heterogeneous sources, we employed a two-stage labeling strategy. For sequences with explicit functional tags in the source databases, a binary label of “1” was directly assigned to the corresponding category. For entries lacking structured annotations but containing descriptive text (e.g., “hemolytic,” “cytotoxic to mammalian cells,” or “active against Gram-positive bacteria”), we applied manual curation and rule-based inference to map free-text evidence to one or more functional categories. This strategy allowed us to recover latent functional information, improve label coverage for underrepresented categories, and ensure semantically consistent annotation across multiple AMP-related functional categories. The overall dataset construction workflow is illustrated in Supplementary Figure S1A.

### S1.2 Data quality control and cleaning pipeline

To ensure dataset reliability and reduce noise arising from low-quality entries or annotation inconsistencies, we implemented a rigorous quality-control pipeline comprising four sequential steps. First, we restricted sequences to lengths between 5 and 200 amino acids, a range aligned with the typical characteristics of antimicrobial peptides(10, 11). Shorter sequences often lack sufficient structural or functional information for reliable prediction, whereas longer sequences are more likely to represent full-length proteins rather than peptides, thereby introducing potential confounding signals.

Second, we excluded sequences containing ambiguous or non-standard amino acid codes (B, J, O, U, X, and Z), as these residues either denote unresolved sequence information or rare modifications that are not consistently supported by standard protein language models and physicochemical encodings. Third, exact duplicates (100% identity) were removed through a hash-based deduplication process. When duplicate entries were identified, we preferentially retained sequences with more complete functional annotations, higher-confidence source records, and richer metadata.

Finally, we verified that all retained sequences consisted solely of the 20 standard amino acids. Sequences showing conflicting functional annotations across databases were manually reviewed, and only labels supported by consistent evidence were retained in the final binary annotation set. This multi-step pipeline yielded a high-quality cleaned sequence collection that served as the foundation for subsequent benchmark and independent validation dataset construction.

### S1.3 Benchmark dataset construction strategy

We refined the original iAMPCN dataset(12) to construct a comprehensive benchmark for systematic functional profiling across 14 AMP-related categories. In the original iAMPCN dataset, AMP sequences were organized into separate single-function groups, which provided incomplete cross-category annotations: each sequence was typically labeled for its reported activity, whereas its status in other functional categories often remained unresolved.

To improve annotation completeness, we aligned the iAMPCN AMP sequences with the multi-source functional annotation resource described above. Labels were standardized into binary annotations, where “1” denotes experimentally supported activity and “0” denotes negative annotation after harmonization. This integration substantially expanded cross-category annotation coverage at the sequence level and enabled more systematic category-specific functional prediction under a shared modeling framework.

As detailed in Supplementary Table S2, this strategy markedly increased positive sample proportions across multiple categories, particularly for relatively rare functions such as cytotoxic (from 0.76% to 13.76%), anti-HIV (from 2.98% to 7.56%), and antioxidant (from 0.22% to 1.90%). These improvements highlight the value of multi-source annotation integration for broadening functional coverage and partially alleviating the severe label imbalance present in the original dataset.

### S1.4 Redundancy Reduction Using CD-HIT

To reduce redundancy and improve the rigor of downstream evaluation, we applied CD-HIT for sequence clustering and redundancy control(13-15). For the benchmark dataset, we followed the established strategy used in AMPfun for endpoint-specific training sets(16). In contrast, the independent validation dataset was subjected to stricter redundancy control to enhance its independence from the benchmark data. First, CD-HIT was applied at a 100% identity threshold to remove any exact matches with benchmark sequences, thereby preventing direct information leakage. Second, an additional clustering step was performed within the validation set at a 40% identity threshold to further reduce internal redundancy and sequence homology. This conservative setting creates a more stringent evaluation scenario, better reflecting real-world AMP discovery conditions in which candidate peptides are often substantially different from previously characterized sequences(17, 18).

### S1.5 Negative Sample Selection Strategy

To construct a high-confidence negative set, we adopted a conservative multi-step strategy aimed at enriching for non-AMP sequences while minimizing contamination by unlabeled or cryptic AMPs. Negative samples were sourced exclusively from the SwissProt subset of UniProt(19-22), which provides manually curated protein records with relatively high annotation quality.

To reduce the likelihood of including sequences with potential antimicrobial or related bioactivities, we applied a keyword-based exclusion filter to UniProt annotations. Sequences were removed if their annotations contained terms directly associated with antimicrobial activity (e.g., “antimicrobial,” “antibacterial,” “antifungal,” “antiviral,” “antiparasitic,” and “bactericidal”), mechanism-related descriptors (e.g., “membrane disruption,” “pore-forming,” “lytic,” and “cytolytic”), functional roles (e.g., “host defense,” “innate immunity,” and “pathogen resistance”), structural motifs (e.g., “defensin,” “cathelicidin,” and “lipopolysaccharide-binding”), bioactivity indicators (e.g., “toxic,” “cytotoxic,” “hemolytic,” “anticancer,” and “antioxidant”), or broader categories such as “antibiotic” and “bioactive peptide.” This exclusion list was manually curated by reviewing annotations of known AMPs to capture commonly used descriptive patterns.

Negative samples were further subjected to the same basic quality-control and redundancy-control criteria as the positive set, including sequence length restriction, removal of ambiguous residues, and CD-HIT-based filtering. In addition, we excluded sequences with long homopolymeric runs (≥10 consecutive identical residues) or extreme compositional bias (any single amino acid accounting for >60% of the sequence), in order to avoid atypical sequence artifacts in the negative set.

This conservative strategy prioritized specificity over sensitivity in negative sample construction, thereby reducing the risk of introducing cryptic AMPs, including encrypted peptides that may be released by proteolytic cleavage(23).

### S2. Feature representation and preprocessing

MAPLE employs a dual-source feature representation strategy that combines contextual sequence embeddings from a pretrained protein language model with explicit knowledge-based physicochemical features. This section describes the construction and preprocessing of the two input streams used throughout the study.

### S2.1 ESM-2 Embeddings

Contextual residue-level embeddings were generated using the pretrained ESM-2 (Evolutionary Scale Modeling 2) protein language model(24). Trained on a large-scale protein sequence corpus, ESM-2 provides high-dimensional sequence representations that capture evolutionary and contextual constraints embedded in protein sequences.

Embeddings were extracted using the official ESM implementation. To efficiently accommodate variable sequence lengths, sequences were processed in dynamic batches with a maximum token limit of 2,048, including special tokens. Computations were performed on CUDA devices in half precision (bfloat16) to improve throughput while maintaining numerical stability.

For each peptide sequence, residue-level representations were obtained from the final encoder layer after removing the beginning-of-sequence (<BOS>) and end-of-sequence (<EOS>) tokens. For a sequence $S=(s_{1},s_{2},\ldots,s_{L})$of length $L$, tokenization yields $T=\langle BOS\rangle,t_{1},t_{2},\ldots,t_{L},\langle EOS\rangle$. A forward pass through ESM-2 produces hidden states $H=ESM\text{\textbackslash mbox}-2(T)$, and the residue-level embedding matrix is extracted as:

$$E_{\mathrm{ESM}}=H[1:L+1,:]$$

These embeddings provide a context-aware representation of each residue and serve as the evolutionary-information branch in the downstream dual-stream architecture.

### S2.2 Knowledge-Based Feature Construction

To complement the ESM-2 embeddings with explicit biochemical and biophysical priors, we constructed a 56-dimensional feature vector for each residue (Supplementary Table S3). These features cover amino acid identity, physicochemical properties, local sequence context, positional information, and global sequence descriptors.

Amino acid identity was encoded using one-hot vectors over the 20 canonical residues, yielding 20 dimensions. Because ambiguous residues had already been excluded during quality control, no additional encoding for non-standard amino acids was required.

Five residue-level physicochemical properties were included: hydrophobicity based on the Kyte-Doolittle scale(25), net charge, normalized molecular weight, α-helix propensity(26), and a binary aromaticity indicator. These descriptors capture properties relevant to membrane interaction and structural preference.

Local sequence context was represented using 15 sliding-window features computed over a 5-residue window centered at each position, with edge padding applied when necessary. These features included proportional compositions of eight residue groups (hydrophobic, charged, polar, aromatic, positive, negative, small, and large), together with derived quantities such as mean hydrophobicity, mean charge, local sequence complexity, and binary indicators for clustered positive charges, hydrophobic residues, amphipathic patterns, and aromatic residues. Such descriptors were intended to capture short-range sequence organization associated with AMP-related local motifs(27).

Positional information was encoded using six dimensions, including normalized residue position, binary indicators for N-terminal, central, and C-terminal regions, normalized distance to the nearest terminus, and a sinusoidal encoding of relative position. These features were included to model position-dependent functional preferences that may influence peptide activity(28).

Finally, ten global sequence descriptors were computed for each peptide and broadcast across all residue positions. These included normalized sequence length, net charge density, average hydrophobicity, estimated isoelectric point, normalized molecular weight, residue diversity, fractions of positive, hydrophobic, and aromatic residues, and a normalized Boman index(29). Broadcasting these sequence-level properties allows local residue representations to be interpreted in the context of the overall peptide physicochemical profile(17).

### S2.3 Knowledge Feature Enhancement via Transformer

Although the 56-dimensional raw knowledge features provide valuable domain priors, they are computed largely in a position-wise manner and therefore lack sequence-wide contextual integration. To address this limitation, we process these vectors through a dedicated four-layer Transformer encoder(30), which enriches them with both local and global sequence context before fusion with the ESM-2 branch.

Let $X_{\mathrm{know}}\in\mathbb{R}^{L\times56}$denote the residue-level knowledge feature matrix for a peptide of length $L$. The encoder first projects these handcrafted features into a higher-dimensional hidden space and adds sinusoidal positional encodings:

$$Z^{\left( 0 \right)}=X_{\mathrm{know}}W_{\mathrm{in}}+b_{\mathrm{in}}+PE$$

where $W_{\mathrm{in}}$and $b_{\mathrm{in}}$are learnable projection parameters and $PE$denotes positional encodings.

The projected features are then passed through $N=4$stacked Transformer blocks. For the $l$-th layer, the contextualized representation is updated as

$$\tilde{Z}^{\left( l \right)}=LN\text{ }\left( Z^{\left( l - 1 \right)} + \mathrm{MHSA}\left( Z^{\left( l - 1 \right)} \right) \right)$$

$$Z^{\left( l \right)}=LN\text{ }\left( \tilde{Z}^{\left( l \right)} + \mathrm{FFN}\left( \tilde{Z}^{\left( l \right)} \right) \right),l=1,\ldots,4$$

where $\mathrm{LN}$denotes layer normalization.

The multi-head self-attention module is defined as

$$MHSA(Q,K,V)=Concat(\mathrm{head}_{1},\ldots,\mathrm{head}_{h})W^{O}$$

with

$$\mathrm{head}_{i}=softmax\left( \frac{QW_{i}^{Q}(KW_{i}^{K})^{\top}}{\sqrt{d_{k}}} + M \right)VW_{i}^{V}$$

where $Q$, $K$, and $V$denote the query, key, and value matrices, $W_{i}^{Q}$, $W_{i}^{K}$, $W_{i}^{V}$, and $W^{O}$are learnable parameters, $d_{k}$is the key dimension, and $M$is a padding mask used to suppress attention to padded positions.

The position-wise feed-forward network is defined as

$$FFN(x)=GELU(xW_{1}+b_{1})W_{2}+b_{2},$$

where $W_{1}$, $W_{2}$, $b_{1}$, and $b_{2}$are learnable parameters.

After the final Transformer layer, the output is projected and normalized to yield the contextualized knowledge representation:

$$E_{\mathrm{know}}=LN\text{ }\left( Z^{\left( 4 \right)}W_{\mathrm{out}} + b_{\mathrm{out}} \right).$$

This Transformer-based enhancement enables the model to integrate handcrafted physicochemical signals across the full sequence and capture interactions among residue properties, local motifs, and positional biases before cross-modal fusion with the ESM-2 branch. The resulting contextualized knowledge features serve as the explicit-prior branch in MAPLE and support downstream AMP identification and category-specific functional prediction(31).

### S3. Model Architecture Details

### S3.1 Overall Architecture Overview

MAPLE is organized as a staged dual-stream architecture operating on two residue-level inputs: contextual ESM-2 embeddings(24) and Transformer-enhanced knowledge features(30). Let $E_{\mathrm{ESM}}\in\mathbb{R}^{L\times d}$and $E_{\mathrm{know}}\in\mathbb{R}^{L\times d}$denote the aligned representations from the two modalities after input projection, where $L$is the peptide length and $d$is the shared hidden dimension. These two inputs are processed in parallel throughout the backbone.

For each modality, the projected features are first passed through a local encoder (CARE) and then through a global encoder (ProBiMamba), yielding four intermediate representations: ESM-CARE, ESM-ProBiMamba, Knowledge-CARE, and Knowledge-ProBiMamba. In this way, MAPLE preserves modality-specific processing while explicitly separating short-range motif refinement from long-range contextual modeling.

Cross-modal interaction is introduced after local and global encoding. Specifically, MAPLE applies four cross-attention modules to model both same-level and cross-level interactions between the two modalities. The resulting fused branches are pooled independently to obtain multiple sequence-level summary vectors, which are then concatenated into a unified peptide representation.

The final concatenated representation is passed to an MLP-based classification head. Under the same architectural template, this head is instantiated for binary AMP identification and separately for each category-specific functional prediction task. Thus, the overall architecture is shared across tasks, whereas model training and output generation are performed independently for AMP identification and for each functional category. Long-range contextual modeling in the ProBiMamba branch is motivated by recent advances in selective state-space models and their efficient sequence modeling properties(32-34).

### S3.2 Input Projection and Dimension Alignment

Before entering the dual-stream backbone, the residue-level representations from the ESM and knowledge branches are projected into a shared base dimension to enable compatible downstream processing and cross-modal fusion. Let $E_{\mathrm{ESM}}\in\mathbb{R}^{L\times d_{\mathrm{esm}}}$and $E_{\mathrm{know}}\in\mathbb{R}^{L\times d_{\mathrm{know}}}$denote the input features from the two modalities, where $L$is the peptide length. Two modality-specific projection modules are used to map them into a common hidden space of dimension $d$:

$$\hat{E}_{\mathrm{ESM}}=Dropout\left( \mathrm{GELU}\left( \mathrm{LN}\left( E_{\mathrm{ESM}}W_{\mathrm{esm}} + b_{\mathrm{esm}} \right) \right) \right),$$

$$\hat{E}_{\mathrm{know}}=Dropout\left( \mathrm{GELU}\left( \mathrm{LN}\left( E_{\mathrm{know}}W_{\mathrm{know}} + b_{\mathrm{know}} \right) \right) \right),$$

where $W_{\mathrm{esm}}\in\mathbb{R}^{d_{\mathrm{esm}}\times d}$and $W_{\mathrm{know}}\in\mathbb{R}^{d_{\mathrm{know}}\times d}$are learnable projection matrices, $b_{\mathrm{esm}}$and $b_{\mathrm{know}}$are bias terms, and $\mathrm{LN}$denotes layer normalization. The Gaussian Error Linear Unit (GELU) activation was used as the nonlinearity because of its smooth response characteristics and favorable optimization behavior in deep networks(35). This modality-specific projection design serves two purposes. First, it aligns evolutionary and knowledge-based features into the same representational space, making subsequent local/global encoding and cross-modal interaction well-defined. Second, the combination of layer normalization, smooth activation, and light dropout helps stabilize optimization while preserving informative residue-level signals. Unless otherwise stated, the aligned representations $\hat{E}_{\mathrm{ESM}}$and $\hat{E}_{\mathrm{know}}$are used as the inputs to the downstream CARE and ProBiMamba modules.

### S3.3 CARE Module: Implementation and Configuration Details

The CARE module, inspired by SCConv(36), is designed to refine local sequence patterns while limiting excessive distortion of the original residue representations. Given an input feature tensor $X\in\mathbb{R}^{L\times d}$, CARE applies three sequential transformations—High-Fidelity Group Normalization (HF-GN), Conservative Motif Extractor (CME), and Adaptive Channel Processor (ACP)—followed by a final gated residual fusion. The overall transformation can be summarized as

$$X_{\mathrm{CARE}}=g\odot X_{\mathrm{ACP}}+(1-g)\odot X$$

where $X_{\mathrm{ACP}}$ denotes the output of the ACP stage, $g$is a learnable gate, and $\odot$ denotes element-wise multiplication. This design enables CARE to preserve informative pretrained signals while selectively enhancing motif-relevant local features.

**High-Fidelity Group Normalization**

HF-GN stabilizes training through a partial normalization strategy that retains a substantial portion of the original input while applying controlled normalization to the remainder. Building on standard Group Normalization(37):

$$GN(X)=\gamma\frac{X-\mu}{\sqrt{\sigma^{2}+\epsilon}}+\beta$$

where $X\in\mathbb{R}^{B\times L\times D}$denotes the input feature tensor; $\mu$ and $\sigma$ are computed across grouped channels; $\varepsilon$ is a small constant to ensure numerical stability; and $\gamma$ and $\beta$ are learnable affine transformation parameters. To reduce the risk that normalization disrupts biologically meaningful structure encoded in pretrained embeddings, HF-GN introduces a retention mechanism:

$$X_{HF-GN}=\alpha\cdot X+\left( 1-\alpha\right)\cdot g_{w}\cdot GN\left( X \right)$$

where $\alpha\in\left[ 0,1 \right]$ controls preservation of the original features and $g_{w}$ is a learnable gate. In this way, HF-GN stabilizes optimization while retaining a controlled amount of the original embedding information.

**Conservative Motif Extractor**

Building on the stabilized representation, CME captures short local sequence patterns that may correspond to functional motifs in AMPs by using parallel 1D convolutional branches with complementary receptive fields(38). The kernel sizes were chosen to reflect biologically relevant local patterns commonly observed in AMPs, such as short charged clusters and amphipathic helical segments(39, 40). Specifically, CME extracts multi-scale local features as:

$$F_{3}=GELU\left( {Conv1D}_{3}\left（ X \right） \right)$$

$$F_{5}=GELU\left( {Conv1D}_{5}\left（ X \right） \right)$$

$$F_{\mathrm{combined}}=Concat\left( F_{3}, F_{5} \right)$$

$$X_{\mathrm{enhanced}}=FFN\left( F_{\mathrm{combined}} \right)$$

An adaptive gate is then estimated from the normalized input:

$$A = \sigma\left( \mathrm{FFN}\left( X \right) \right)$$

and the motif-enhanced representation is fused conservatively with the original signal:

$$X_{\mathrm{CME}}=\alpha\cdot X+\left( 1-\alpha\right)\cdot\left( A\odot X_{\text{e}\text{nhanced }} \right)$$

where $\alpha\in\left[ 0,1 \right]$ controls retention of the original representation and $\odot$ denotes element-wise multiplication. This conservative fusion strategy is intended to enhance motif-sensitive features without discarding the broader sequence context, which is important because AMP-relevant motifs are typically expressed through localized residue combinations rather than isolated residues.

**Adaptive Channel Processor**

ACP performs global channel reweighting inspired by squeeze-and-excitation mechanisms(41), but tailored for protein sequence representations. By aggregating sequence-wide statistics and passing them through a compact bottleneck network, ACP learns to emphasize feature channels that are most informative for the entire peptide, such as those associated with charge or hydrophobicity. The channel-selection weight is computed as:

$$w_{c} = \sigma\left( \mathrm{Linear}\left( \mathrm{GlobalAvgPool}\left( X_{\mathrm{CME}} \right) \right) \right)$$

Two transformation branches with different compression characteristics are then applied:

$$X_{\text{low }}=\mathrm{Processor}_{\text{low }}\left( X_{\mathrm{CME}} \right), X_{\text{high }}=\mathrm{Processor}_{\text{high }}\left( X_{\mathrm{CME}} \right)$$

and combined adaptively as:

$$X_{\text{mixed }}=w_{c}\odot X_{\text{low }}+\left( 1-w_{c} \right)\odot X_{\text{high }}$$

The mixed features are then fused with the original motif-enhanced representation:

$$X_{\mathrm{ACP}}= FFN\left( \mathrm{Concat}\left( \left[ X_{\mathrm{CME}},X_{\mathrm{mixed}} \right] \right) \right)$$

This stage enables the model to adaptively emphasize coarse or fine channel transformations depending on peptide-specific representation patterns. Unless otherwise stated, the same CARE design is applied independently to both the ESM branch and the knowledge branch.

### S3.4 ProBiMamba Module: Bidirectional Mamba-inspired Sequence Mixer for Peptides

ProBiMamba is implemented as a lightweight bidirectional sequence encoder inspired by recent developments in selective state-space sequence modeling(32-34). In this study, it is employed as a compact peptide-oriented module with identical hyperparameters for both the ESM and knowledge branches. Gradient checkpointing is further enabled during training to reduce memory usage.

After input projection and internal positional augmentation, the sequence representation is processed by a bidirectional gated sequence-mixing unit. Given an input representation $X\in\mathbb{R}^{L\times d}$, a linear projection first splits the representation into a content stream and a gate stream:

$$[X_{\mathrm{proj}},\text{ }G]=Linear(X)$$

where both $X_{\mathrm{proj}}$and $G$have hidden dimension $d$.

The content stream is then processed in forward and reverse directions:

$$Y_{\to}=\mathrm{SSM}_{\to}(X_{\mathrm{proj}}),Y_{\leftarrow}=Flip\left( \mathrm{SSM}_{\leftarrow}(Flip(X_{\mathrm{proj}})) \right)$$

In the current implementation, each directional $\mathrm{SSM}$core is realized as a gated convolutional mixing operator:

$$SSM(X)=\omega X+(1-\omega)\left( \sigma(W_{g}X)\odot ReLU(BN(Conv1D(X))) \right)$$

where $\omega$is a learnable skip weight, $W_{g}$is a learnable gate projection, $Conv1D$denotes a 1D convolution with kernel size 3, and $\mathrm{BN}$denotes batch normalization. This formulation preserves a residual path while introducing direction-aware sequence mixing with low computational overhead.

The forward and backward outputs are concatenated and fused:

$$Y_{\mathrm{bi}}=ReLU\left( \mathrm{LN}\left( [Y_{\to};Y_{\leftarrow}]W_{f}+b_{f} \right) \right)$$

and the fused representation is modulated by the gate stream:

$$Y_{\mathrm{mamba}}=X+OutProj\left( Y_{\mathrm{bi}}\odot\sigma(G) \right)$$

This bidirectional gated design allows the module to capture N-to-C and C-to-N contextual dependencies while retaining sensitivity to position-specific sequence patterns.

Each BiMambaBlock adopts a two-branch formulation consisting of a bidirectional sequence-mixing path and a feed-forward path. For the $l$-th block, the two branch outputs are:

$$Y_{\mathrm{mamba}}^{\left( l \right)}=BiDirectionalMamba\left( \mathrm{LN}_{1}\left( X^{\left( l - 1 \right)} \right) \right)$$

$$Y_{\mathrm{ffn}}^{\left( l \right)}=FFN\left( \mathrm{LN}_{2}\left( X^{\left( l - 1 \right)} \right) \right)$$

Their relative contributions are controlled by learnable component weights normalized by softmax:

$$[\alpha_{1},\alpha_{2}]=softmax(\theta)$$

and the block output is defined as:

$$X^{\left( l \right)}=X^{\left( l - 1 \right)}+Dropout\left( \alpha_{1}Y_{\mathrm{mamba}}^{\left( l \right)} + \alpha_{2}Y_{\mathrm{ffn}}^{\left( l \right)} \right)$$

Given an input feature sequence $E$, the encoder first applies an input projection:

$$X^{\left( 0 \right)}=InputProj(E),$$

followed by two stacked BiMambaBlocks:

$$X^{\left( l \right)}=\mathrm{Block}_{l}(X^{\left( l - 1 \right)}),l=1,2$$

The final contextualized representation is obtained via layer normalization:

$$E_{\mathrm{ProBiMamba}}=LN(X^{\left( 2 \right)})$$

The outputs from the ESM-ProBiMamba and Knowledge-ProBiMamba branches are subsequently normalized and passed to the downstream cross-modal fusion modules.

### S3.5 Cross-Modal Fusion: Four-Level Integration Strategy

In this study, MAPLE integrates the ESM and knowledge branches through four independent cross-modal attention modules. Fusion is performed over four paths: same-level fusion between the two CARE outputs, same-level fusion between the two ProBiMamba outputs, and two cross-level fusion pathways linking the ESM local representation to the knowledge global representation and the ESM global representation to the knowledge local representation. This design preserves complementary information from local motif refinement and global contextual modeling in both modalities(42, 43).

Let

$$E_{C}\in\mathbb{R}^{L\times480},E_{P}\in\mathbb{R}^{L\times240},K_{C}\in\mathbb{R}^{L\times480},K_{P}\in\mathbb{R}^{L\times240}$$

denote the outputs of the ESM-CARE, ESM-ProBiMamba, Knowledge-CARE, and Knowledge-ProBiMamba branches, respectively. For a generic pair of inputs $x_{1}\in\mathbb{R}^{L\times d_{1}}$and $x_{2}\in\mathbb{R}^{L\times d_{2}}$, the cross-modal attention module first projects the two modalities into a shared hidden space $d_{h}=min(d_{1},d_{2})$:

$$Q_{1}=x_{1}W_{Q}^{\left( 1 \right)},K_{2}=x_{2}W_{K}^{\left( 2 \right)},V_{2}=x_{2}W_{V}^{\left( 2 \right)},$$

$$Q_{2}=x_{2}W_{Q}^{\left( 2 \right)},K_{1}=x_{1}W_{K}^{\left( 1 \right)},V_{1}=x_{1}W_{V}^{\left( 1 \right)}.$$

Bidirectional cross-attention is then computed as

$$A_{1\leftarrow2}=softmax\left( \frac{Q_{1}K_{2}^{\top}}{\sqrt{d_{h}}} \right),A_{2\leftarrow1}=softmax\left( \frac{Q_{2}K_{1}^{\top}}{\sqrt{d_{h}}} \right),$$

followed by modality-specific output projections:

$$O_{1}=A_{1\leftarrow2}V_{2}W_{O}^{\left( 1 \right)},O_{2}=A_{2\leftarrow1}V_{1}W_{O}^{\left( 2 \right)}.$$

The updated representations are obtained through residual fusion:

$$x_{1}^{'}=x_{1}+O_{1},x_{2}^{'}=x_{2}+O_{2}.$$

This bidirectional design enables each modality to incorporate complementary information from the other while preserving its own original representation. The attention computation follows the standard scaled dot-product formulation(30).

In this study, fusion is carried out in two stages. First, same-level fusion is applied to the local branches and the global branches:

$$(\tilde{E}_{C},\tilde{K}_{C})=\mathcal{F}_{\mathrm{care}}(E_{C},K_{C}),$$

$$(\tilde{E}_{P},\tilde{K}_{P})=\mathcal{F}_{\mathrm{prob}}(E_{P},K_{P}).$$

Second, cross-level fusion is performed to exchange information between local and global representations across modalities:

$$(\hat{E}_{C},\hat{K}_{P})=\mathcal{F}_{\times1}(\tilde{E}_{C},\tilde{K}_{P}),$$

$$(\hat{E}_{P},\hat{K}_{C})=\mathcal{F}_{\times2}(\tilde{E}_{P},\tilde{K}_{C}),$$

where $\mathcal{F}_{\mathrm{care}},\mathcal{F}_{\mathrm{prob}},\mathcal{F}_{\times1}$, and $\mathcal{F}_{\times2}$are four independently parameterized cross-modal attention modules.

At the sequence level, each fused branch is pooled independently by mean aggregation:

$$z_{E_{C}}=Mean(\hat{E}_{C}),z_{E_{P}}=Mean(\hat{E}_{P}),$$

$$z_{K_{C}}=Mean(\hat{K}_{C}),z_{K_{P}}=Mean(\hat{K}_{P}).$$

The final fused peptide representation is formed by concatenation:

$$z_{\mathrm{final}}=[\text{ }z_{E_{C}};z_{E_{P}};z_{K_{C}};z_{K_{P}}\text{ }].$$

In this study, the final fusion dimension is

$$480+240+480+240=1440,$$

and the concatenated representation is then passed to the downstream classification head after layer normalization.

### S3.6 Classification Head and Output Layer

In this study, the fused peptide representation obtained after four-path cross-modal integration is first normalized and regularized before classification. Let $z_{\mathrm{final}}\in\mathbb{R}^{1440}$denote the concatenated sequence-level feature vector. The model first applies layer normalization, followed by an additional dropout layer with probability $0.3$:

$$\tilde{z}=\mathrm{Dropout}_{0.3}(LN(z_{\mathrm{final}}))$$

The normalized feature vector is then passed to a task-specific multi-layer perceptron classifier. For each independently trained task, the classifier consists of two hidden fully connected layers with LeakyReLU activation and dropout, followed by a final linear output layer:

$$h_{1}=Dropout\text{ }\left( \mathrm{LeakyReLU}\left( W_{1}\tilde{z}+b_{1} \right) \right)$$

$$h_{2}=Dropout\text{ }\left( \mathrm{LeakyReLU}\left( W_{2}h_{1}+b_{2} \right) \right)$$

$$o=W_{3}h_{2}+b_{3}$$

In the present study, the hidden dimension of the classifier is set to 1024, and the dropout probability within the classifier is set to 0.8. The final output layer produces task-specific logits, where the head is instantiated separately for AMP identification and for each category-specific functional prediction task. Under this setting, each task produces a single logit.

To improve numerical stability, the output logits are constrained to a bounded range:

$$\hat{o}=clip(o,\text{ }-10,\text{ }10)$$

This bounded-output design helps avoid extreme logit values during optimization while preserving the relative ordering of predictions.

### S4. Evaluation and uncertainty quantification

MAPLE was evaluated in two task settings: binary AMP identification and category-specific functional prediction across 14 AMP-related activities. AMP identification was treated as a standard binary classification problem. Functional profiling was implemented as a set of 14 independently trained binary classifiers, one for each activity category, and overall functional performance was summarized across categories using macro-averaged metrics. All evaluations were conducted on the benchmark dataset and the independent validation dataset described in the main text.

### S4.1 Evaluation metrics for AMP discrimination

For binary AMP identification, model performance was assessed using Accuracy, Precision, Sensitivity (Recall), Specificity, F1 score, Matthews correlation coefficient (MCC), area under the receiver operating characteristic curve (AUROC), and area under the precision-recall curve (AUPRC). Let $TP$, $TN$, $FP$, and $FN$denote the numbers of true positives, true negatives, false positives, and false negatives, respectively. The threshold-dependent metrics were defined as

$$Accuracy=\frac{TP+TN}{TP+TN+FP+FN}$$

$$Precision=\frac{TP}{TP+FP}$$

$$Recall=\frac{TP}{TP+FN}$$

$$Specificity=\frac{TN}{TN+FP},$$

$$F1=\frac{2\cdot Precision\cdot Recall}{Precision+Recall}$$

$$MCC=\frac{TP\cdot TN-FP\cdot FN}{\sqrt{\left( TP+FP)(TP+FN)(TN+FP)(TN+FN \right)}}$$

Threshold-independent discrimination was assessed using AUROC and AUPRC. AUROC summarizes ranking performance across all possible decision thresholds, whereas AUPRC is particularly informative under class imbalance because it emphasizes retrieval quality for the positive class. During checkpoint monitoring, hard predictions for AMP identification were generated using the default threshold of 0.5.

### S4.2 Evaluation and uncertainty quantification

For functional prediction, each AMP-related activity category was evaluated as an independent binary classification task. Performance was first computed separately for each category and then summarized across the 14 classifiers using macro-averaged metrics:

$$Macro-Precision=\frac{1}{14}\sum_{t=1}^{14} \mathrm{Precision}_{t}$$

$$Macro-Sensitivity=\frac{1}{14}\sum_{t=1}^{14} \mathrm{Sensitivity}_{t}$$

$$Macro-F1=\frac{1}{14}\sum_{t=1}^{14} {F1}_{t}$$

$$Macro-AUROC=\frac{1}{14}\sum_{t=1}^{14} \mathrm{AUROC}_{t}$$

This macro-averaging strategy assigns equal weight to each activity category and therefore provides a balanced summary across common and rare functions. In addition to the aggregated results, per-category metrics were also examined to identify which activities were modeled more reliably and which remained more challenging.

To quantify uncertainty in all reported metrics, 95% confidence intervals were estimated using bootstrap resampling with 1,000 iterations. For each dataset and each metric, samples were resampled with replacement, the corresponding metric was recomputed on each bootstrap replicate, and the 2.5th and 97.5th percentiles of the empirical bootstrap distribution were taken as the lower and upper confidence bounds, respectively. For AMP identification, bootstrap resampling was applied directly to the binary test set. For functional prediction, bootstrap resampling was applied at the instance level, and both category-specific metrics and their macro-averaged summaries were recalculated for each replicate. This procedure was applied consistently to both the benchmark dataset and the independent validation dataset.

### S5. Interpretability and motif analyses

### S5.1 UMAP visualization of peptide embedding space

To qualitatively assess how AMPs and their functional subtypes are distributed in the learned representation space, we performed Uniform Manifold Approximation and Projection (UMAP) analysis on sequence-level embedding vectors(44-46). As a nonlinear dimensionality reduction technique, UMAP preserves local neighborhood structures in high-dimensional spaces and has been widely used for visualizing protein and peptide representations(47). Specifically, for each peptide sequence, we first generated a fixed-length sequence embedding by mean-pooling the residue-level model outputs. For the MAPLE model, we used the final fused sequence representation immediately before the classification head; for baseline models, we employed their respective sequence-level representations under the same pooling strategy. To mitigate interference caused by scale differences across models or datasets, all embeddings were L2-normalized. When the original embedding dimensionality was high, we further applied principal component analysis (PCA) for initial dimensionality reduction, which helped retain dominant information while reducing noise and improving computational efficiency for subsequent UMAP processing.

UMAP was performed using a standardized configuration, including cosine distance, spectral initialization, and a fixed random seed. The resulting two-dimensional coordinates were visualized under two label settings: AMP versus non-AMP status and functional AMP subtypes. For subtype visualization, peptides positive for a given functional category were highlighted against a low-opacity background of other peptides, reducing visual dominance by high-prevalence categories while preserving rare positive instances. When necessary, background samples were downsampled to improve readability. A consistent preprocessing and UMAP configuration was used within each comparative visualization to reduce artifacts caused by inconsistent parameter choices.

### S5.2 K-mer enrichment analysis protocol

To identify short sequence patterns associated with specific antimicrobial and toxicity-related functions, we performed systematic k-mer enrichment analysis in a one-versus-rest framework across all functional categories(48, 49). For each target function, peptides annotated as positive for that label were treated as the foreground set, while all remaining peptides in the benchmark dataset used for category-specific functional prediction served as the background set. This design enables quantification of sequence features that are selectively enriched for a given biological activity while controlling for global compositional biases in the antimicrobial peptide sequence space.

For each sequence, all overlapping k-mers of length $k$were extracted using a sliding window with step size 1. K-mer lengths from 3 to 7 were initially evaluated to compare motif resolutions. To ensure statistical robustness and reduce noise from extremely rare patterns, k-mers occurring fewer than five times in the combined foreground and background sets were excluded from downstream analysis. For each retained k-mer $k$and target category $t$, enrichment was evaluated using Fisher’s exact test(50), with motif occurrence counted at the sequence level so that each sequence contributed once according to whether it contained the motif. Consistent with the main Methods, enrichment magnitude was quantified as log₂ enrichment with a pseudocount of 1 to avoid undefined ratios when a motif was absent from either the foreground or background set. Multiple testing was controlled using the Benjamini–Hochberg procedure(51). Volcano plots were used to summarize motif-level enrichment, with the x-axis representing log₂ enrichment and the y-axis representing −log₁₀ of the FDR $P$ value. Motifs passing FDR correction were considered statistically significant, and enrichment magnitude was used for visualization and downstream prioritization where specified.

### S5.3 Rationale for motif-length selection and downstream comparative analyses

To select an appropriate motif length, we compared k-mer sizes from 3 to 7 based on the number of significantly enriched motifs, enrichment magnitude, sequence coverage, and the degree of cross-category motif overlap.

For antibacterial and hemolytic activities, overlaps between significantly enriched 7-mers were quantified to assess the extent of shared sequence patterns. For shared motifs, the association between antibacterial and hemolytic enrichment strengths was evaluated using Pearson and Spearman correlation coefficients. To further examine how hemolytic overlap varied along the antibacterial enrichment axis, antibacterial-enriched 7-mers were stratified by their log₂ enrichment values, and the proportion overlapping with hemolysis-enriched motifs was calculated within each stratum.

For each significantly enriched 7-mer, physicochemical properties, including net charge, mean hydrophobicity, amphipathicity, and predicted α-helical propensity, were computed based on residue-level scales. Motifs were grouped into antibacterial-selective, hemolysis-selective, and dual-activity categories according to their enrichment profiles, and group-level differences in physicochemical properties were assessed using the Kruskal–Wallis test where appropriate.

### S5.4 Virtual peptide library generation and novelty filtering

A virtual peptide library was constructed to support downstream proof-of-concept computational screening. Peptide lengths were uniformly sampled between 15 and 40 residues. Sequences were generated by random sampling under a biased amino-acid distribution informed by the motif-level enrichment analysis and physicochemical profiling, favoring moderate cationicity while maintaining controlled overall hydrophobicity. Specifically, lysine and arginine residues were modestly enriched, and hydrophobicity was constrained according to the Eisenberg scale(52).

To ensure novelty relative to existing data, sequences identical to any peptide in either the benchmark AMP dataset or the independent validation dataset were discarded. To further reduce near-duplicate candidates, an edit-distance-based filtering strategy was applied: sequences with a Levenshtein distance ≤ 2 to any known peptide in either dataset were excluded. For all retained sequences, net charge and mean hydrophobicity were computed using the same definitions as described above. The resulting virtual library was stored in tabular format with basic sequence descriptors and subsequently used for MAPLE-based inference and antibacterial–hemolysis prioritization.

**Supplementary Results**

### R1. Sequence characterization

### R1.1 Benchmark dataset characterization

To characterize the structural and functional properties of the benchmark dataset, we analyzed both the distribution of functional annotations and the underlying sequence features of antimicrobial and non-antimicrobial peptides (Supplementary Figs. S2 and S4).

Functional labels exhibited pronounced imbalance across categories (Supplementary Figure S2A). General antibacterial activity was the most prevalent annotation, whereas several clinically relevant functions, including anti-HIV, antioxidant, and antiparasitic activity, were comparatively rare(7, 52). Despite this imbalance, AMPs displayed extensive multifunctionality: 71.5% of AMPs were annotated with two or more functional labels, whereas only 28.5% carried a single annotation (Supplementary Figure S2C). The distribution of label counts per sequence further confirmed that multifunctional peptides constitute the dominant fraction of the dataset (Supplementary Figure S2B). Conditional co-occurrence analysis revealed strong dependencies among several efficacy-related categories, particularly between antibacterial, antibiofilm, and antigram-positive activities, as well as partial coupling between efficacy- and toxicity-related functions such as hemolytic and anti-mammalian cell activity (Supplementary Figure S2D). These patterns highlight the co-annotation structure of AMP functional space and motivate coordinated evaluation of antimicrobial efficacy- and host-toxicity-related endpoints.

Compositional analysis further revealed systematic biochemical differences between AMP and non-AMP sequences (Supplementary Figure S4B). AMPs were strongly enriched in lysine (K) and arginine (R), conferring high positive charge for electrostatic attraction to negatively charged microbial membranes(53-55), and showed elevated frequencies of leucine (L) and glycine (G), supporting membrane insertion and structural flexibility(55, 56). In contrast, non-AMPs contained slightly higher proportions of acidic residues such as aspartate (D) and glutamate (E), consistent with their distinct intracellular functional roles.

Stratification by functional category demonstrated that most AMP subclasses consist predominantly of short peptides; however, anticancer and antiviral peptides displayed broader length distributions, including substantially longer sequences (Supplementary Figure S4C)(52, 57). This pattern is consistent with the presence of precursor peptides requiring proteolytic processing or modular architectures combining targeting and effector domains. Moreover, amino-acid usage varied systematically across functional categories (Supplementary Figure S4D). Hemolytic, cytotoxic, and anti-mammalian peptides shared highly similar compositions, characterized by enrichment of lysine and leucine(54, 55), suggesting a common cationic amphipathic mechanism underlying membrane disruption in eukaryotic cells. In contrast, antioxidant and anti-HIV peptides exhibited distinct residue preferences, consistent with mechanisms involving specific molecular interactions with viral proteins or host receptors rather than nonspecific membrane permeabilization.

These analyses demonstrate that the benchmark dataset is characterized by severe label imbalance, pervasive co-annotation, and pronounced sequence-level heterogeneity across functional categories, motivating category-specific functional profiling and coordinated evaluation of antimicrobial efficacy- and host-toxicity-related endpoints.

### R1.2 Independent validation dataset characterization

To assess the structural validity and complementary role of the independent validation dataset, we analyzed its functional label distribution and sequence features and compared them with those of the benchmark dataset (Supplementary Figs. S3 and S5).

Similar to the benchmark dataset, functional annotations in the validation dataset exhibited pronounced imbalance, with general antibacterial activity being the most prevalent category and several clinically relevant functions, such as anti-HIV and antioxidant activity, remaining rare (Supplementary Figure S3A). In contrast to the benchmark dataset, the validation set contained a larger proportion of single-label peptides (79.9%), with only 20.1% of AMPs annotated with two or more functional labels (Supplementary Figure S3B–C), indicating a lower degree of multifunctionality. Nevertheless, conditional co-occurrence analysis revealed consistent dependency patterns among major efficacy-related functions and partial coupling between efficacy- and toxicity-related categories, including hemolytic and anti-mammalian cell activities (Supplementary Figure S3D), suggesting that the core functional relationships are preserved across datasets.

At the sequence level, AMPs in the validation set were slightly longer on average than those in the benchmark dataset (mean length 55.8 vs. 30.1 residues), yet remained markedly shorter than non-AMP sequences, retaining the compact structural characteristics typical of antimicrobial peptides (Supplementary Figure S5A)(52, 57). Amino-acid composition profiles were highly consistent between datasets (Supplementary Figure S5B), with AMPs enriched in cationic residues (lysine and arginine) and hydrophobic residues such as leucine(54-56), supporting conserved cationic amphipathic membrane-active properties. Minor differences in residue frequencies were observed across several functional categories (Supplementary Figure S5C–D), indicating modest distributional shifts that introduce additional sequence diversity without altering the fundamental biochemical signatures of AMP classes. These results indicate that the independent validation dataset is sequence-non-redundant with the benchmark dataset while retaining broadly comparable biochemical and functional characteristics, providing a non-overlapping validation setting for assessing model generalization under modest dataset shift.

### R2 Model Comparison and Evolution

### R2.1 Models Methodological Comparison and Evolution of AMP Prediction Models

Recent advances in computational peptide design have prompted a methodological shift in AMP prediction—from handcrafted feature engineering toward deep, pretraining-based representations. To contextualize the positioning of our proposed MAPLE model within this evolving landscape, we conducted a structured comparison of 11 representative AMP prediction models spanning three methodological generations. Supplementary Table S4 summarizes key distinctions in algorithmic foundations, feature representation paradigms, prediction scopes, and model interpretability.

Early-generation approaches, such as AI4AMP(58), were built upon manually curated physicochemical descriptors (e.g., PC6 features), which—despite their domain relevance—suffered from limited expressiveness and constrained generalizability. In the intermediate generation, models such as iAMPCN(12) and AMPpred-MFA(59) introduced deep learning modules (e.g., CNNs, BiLSTMs) to learn local and sequential patterns directly from raw amino acid sequences. While these models represented a step forward in automated feature learning, they typically required large labeled datasets and lacked architectural flexibility for broader functional endpoint coverage.

Recently, models such as UniDL4BioPep(31), PLAPD(60), and our proposed MAPLE have adopted PLMs like ESM-2(24) as foundational encoders. Trained on billions of unlabeled protein sequences, PLMs capture deep evolutionary, structural, and functional patterns inherent in the protein “language”(24). This pretraining–fine-tuning paradigm enables effective transfer of general protein knowledge to downstream AMP prediction tasks(40), significantly enhancing model performance and robustness. For instance, PLAPD combines ESM-2 embeddings with a residual Transformer architecture, highlighting the power of PLMs in feature representation.

### R2.2 Selectivity assessment via dual prediction

To support selectivity-oriented decision making in AMP development, we performed a dual-prediction analysis that jointly considers antibacterial efficacy and hemolytic liability. For each peptide in the independent validation dataset, MAPLE outputs a predicted antibacterial probability $P_{antibacterial}$ and a predicted hemolysis probability $P_{hemolytic}$. We visualized peptides in this two-dimensional probability space and applied an antibacterial decision threshold to separate peptides predicted as antibacterial from those predicted as non-antibacterial (Supplementary Figure S7A), enabling direct inspection of candidate regions that combine high predicted antibacterial activity with low predicted hemolysis risk. To facilitate interpretation and downstream triage, we further stratified peptides into three hemolysis risk tiers (low, moderate, high) using predefined $P_{hemolytic}$ boundaries (horizontal reference lines in Supplementary Figure S7A), and summarized the tier composition among predicted antibacterial candidates (Supplementary Figure S7B), with 95% confidence intervals estimated by bootstrap resampling (Supplementary Figure S7C). Beyond discrete tiers, we compared the full distribution of P(hemolytic) between all peptides and the predicted antibacterial subset using cumulative distribution functions (Supplementary Figure S7D), providing a threshold-free view of how hemolysis propensity shifts when conditioning on predicted antibacterial activity. Finally, we directly contrasted hemolysis probability distributions below versus above the antibacterial threshold using violin/box summaries (Supplementary Figure S7E), offering an intuitive diagnostic of efficacy–toxicity coupling: a separable distribution supports the presence of sequence-space regions where antibacterial activity can be achieved with reduced hemolytic risk(61, 62).

### R2.3 Embedding space visualization

To examine whether antibacterial activity and hemolytic liability occupy separable regions in MAPLE’s learned representation, we visualized peptides in the fused embedding space using UMAP(46). Antibacterial and hemolytic peptides were projected into two dimensions and displayed as blue and red points, respectively, with kernel-density contours summarizing their spatial distributions (Supplementary Figure S8). The two distributions show substantial overlap within the dominant manifold, consistent with shared membrane-interaction determinants, yet exhibit systematic shifts in their density peaks, indicating that MAPLE’s fused representation captures non-identical sequence features underlying efficacy and toxicity. To quantify this shift, we computed the centroid of each class in the 2D UMAP plane and measured the Euclidean inter-centroid distance (dashed line), yielding a distance of 0.4936 with a 95% confidence interval of 0.3797–0.6470 estimated by bootstrap resampling. Together, these results provide a geometry-based, model-internal view supporting the selectivity analyses: antibacterial and hemolytic peptides are not fully separable in sequence space, but their representation-level distributions are measurably offset(63), enabling downstream stratification of candidates by predicted efficacy–toxicity profiles.

### R3. Motif-level findings

### R3.1 K-mer length optimization

To select an appropriate motif granularity for enrichment analysis, we systematically compared k-mer lengths from 3 to 7 across all 14 functional categories (Supplementary Figure S9), balancing motif discoverability, functional specificity, overlap structure, and sequence coverage. First, the number of significantly enriched motifs varies with k (Supplementary Figure S9A). While 5-mers produced the highest average number of significant k-mers (2932 ± 2793), they also showed substantial inter-category variability (CV = 0.92), indicating that discovery counts were less stable across functions of different breadth. In contrast, 7-mers yielded a comparable but more consistent enrichment yield (2454 ± 2113, CV = 0.86), supporting more uniform motif discovery across labels. Second, enrichment strength increased monotonically with k (Supplementary Figure S9B): mean log₂ fold-change rose from 1.65 ± 0.99 (3-mer) to 7.95 ± 3.07 (7-mer). Notably, the gain largely plateaued between 6-mer (7.63 ± 3.18) and 7-mer, suggesting that extending motifs beyond 6 residues provides limited additional effect-size increase, but still stabilizes the signal (slightly lower variance at 7-mer). Third, cross-category overlap analysis (Supplementary Figure S9C) indicates that short motifs tend to produce clustered high-overlap blocks, consistent with non-specific sharing driven by broad compositional signatures. Although the overall mean overlap remained similar between 3-mer (31.7%) and 7-mer (30.5%), the overlap pattern at 7-mer became more dispersed, reflecting greater functional independence. Importantly, strong overlaps persisted primarily between mechanistically related functions, such as antibacterial–antibiofilm (>70%) and cytotoxic–hemolytic (>60%), supporting biological rather than artifactual sharing. Fourth, an illustrative lineage (Supplementary Figure S9D) demonstrates progressive specialization as motifs are extended: the 3-mer “IAG” was broadly enriched across six categories with modest effect sizes (log₂ FC ~1.2), whereas extension to “VIAGY” restricted enrichment to three categories with markedly increased magnitude (~8.0), and the 7-mer “TVIAGYN” retained this specificity while reaching maximal enrichment (log₂ FC = 10.00). Finally, the coverage–specificity trade-off (Supplementary Figure S9E) shows that total unique k-mers peaked at 4-mer (~26,500) but with the lowest enrichment rate (~10%), whereas 7-mer maintained sufficient coverage (~10,000 k-mers) with a substantially higher enrichment rate (~28%).

Collectively, the stable discovery profile (CV 0.86), near-maximal enrichment magnitude (7.95 ± 3.07), reduced non-specific overlap structure, and favorable coverage–specificity operating point motivated our choice of 7-mers for all downstream motif analyses(16, 64).

### R3.2 Antibacterial vs hemolytic overlap

To quantify sequence determinants shared between antibacterial efficacy and hemolytic liability, we compared significantly enriched 7-mer motifs(65) identified for the antibacterial and hemolytic labels using the same one-versus-rest enrichment framework and multiple-testing control described in Supplementary Methods. Enriched motifs were visualized as paired volcano plots, where the x-axis denotes enrichment effect size (log_2_ enrichment) and the y-axis denotes statistical significance (−log_10_ P) (Supplementary Figure S10).

In the antibacterial volcano plot Supplementary Figure S10A), antibacterial-specific motifs are highlighted in blue, whereas motifs that are also significantly enriched for hemolysis are marked as shared (purple). The hemolytic volcano plot (Supplementary Figure S10B) uses the analogous scheme, with hemolysis-specific motifs in orange and shared motifs again in purple. Across both panels, a prominent fraction of statistically significant motifs fall into the shared set, indicating that many sequence patterns contributing to predicted antibacterial activity are also associated with hemolytic propensity. This overlap is most evident among highly enriched motifs, where shared k-mers occupy the right-hand region of the volcano plots (large positive log₂ enrichment with strong significance), consistent with the idea that strongly membrane-active motifs can drive both bacterial killing and host-cell lysis.

At the same time, both panels retain clear activity-specific motifs (blue-only or orange-only), demonstrating that antibacterial efficacy and hemolysis are not governed by an identical motif repertoire. The coexistence of shared and activity-specific patterns supports a nuanced selectivity landscape: there is meaningful mechanistic coupling between efficacy and toxicity at the motif level, yet distinct motifs remain that may be leveraged to prioritize antibacterial candidates with reduced hemolytic risk(61, 62).

### R3.3 Other functions enrichment maps

To extend motif-level interpretation beyond antibacterial and hemolytic activities, we performed the same one-versus-rest 7-mer enrichment analysis for eight additional functional categories and summarized their enrichment landscapes using volcano plots (Supplementary Figure S11). For each function, the x-axis represents enrichment effect size (log_2_ enrichment) and the y-axis represents statistical significance (−log_10_P), with significantly enriched motifs highlighted in red after multiple-testing correction (FDR < 0.05). This visualization provides a compact, function-specific “motif signature map” that captures both the strength and confidence of enriched sequence patterns.

Across most functions, enriched motifs predominantly appear on the positive-enrichment side, indicating selective accumulation of motifs in peptides annotated with the target activity relative to the remaining AMP background. Notably, related antibacterial subtypes—including antigram-negative, antigram-positive, and anti-MRSA—exhibit broadly similar volcano-plot geometries, with dense clusters of significant motifs spanning moderate-to-strong enrichment and a subset of highly significant outliers (Supplementary Figure S11A–C). This consistency supports the presence of shared antibacterial sequence determinants across bacterial targets(27), while still permitting subtype-specific motifs that may reflect differences in outer-membrane permeability barriers (Gram-negative) or cell-wall interactions (Gram-positive/MRSA).

In contrast, functions reflecting distinct biological mechanisms show enrichment landscapes with different shapes and sparsity. Antibiofilm activity displays a pronounced set of strongly enriched motifs (Supplementary Figure S11D), consistent with specialized sequence patterns associated with surface interaction and biofilm disruption. Anti-HIV motifs also show a broad distribution of significant enrichments (Supplementary Figure S11E), suggesting multiple motif families may contribute to antiviral mechanisms. For antiparasitic and antioxidant activities, enriched motifs are fewer and more concentrated toward higher effect sizes (Supplementary Figure S11F–G), consistent with narrower sequence determinants and/or greater annotation scarcity. Finally, cytotoxic activity presents a prominent enrichment pattern (Supplementary Figure S11H) that mirrors the general membrane-active motif logic observed in hemolysis-related analyses, supporting mechanistic proximity between mammalian cytotoxicity and membrane disruption.

Collectively, these enrichment maps demonstrate that MAPLE’s motif analysis pipeline captures both shared motif repertoires among related functions and distinct enrichment signatures for mechanistically different activities, providing a scalable, interpretable view of function-selective sequence patterns across the multi-label AMP landscape.

### R3.4 Motif logos & categories

To provide an intuitive, residue-level view of the sequence patterns captured by the 7-mer enrichment analysis, we summarized representative motifs using sequence logos stratified by activity category: antibacterial-selective, hemolytic-selective, and dual-activity (shared) motifs (Supplementary Figure S12). Motifs were assigned to each category based on their enrichment significance profiles: antibacterial-selective motifs were significantly enriched only in the antibacterial foreground set, hemolytic-selective motifs only in the hemolytic foreground set, and dual-activity motifs were significantly enriched in both analyses.

Across all three categories, the logos reveal a pronounced enrichment of canonical AMP physicochemical signatures, including cationic residues (K/R) and hydrophobic residues (L/I/V/A), consistent with membrane-active mechanisms(66, 67). Antibacterial-selective motifs (Supplementary Figure S12A–C) exhibit strong cationic dominance, often with dense K/R at multiple positions, supporting bacterial membrane targeting and electrostatic attraction to anionic bacterial surfaces. Hemolytic-selective motifs (Supplementary Figure S12D–F) show a comparatively stronger hydrophobic component and more continuous hydrophobic stretches (e.g., L-rich patterns), consistent with enhanced insertion into zwitterionic mammalian membranes and increased lytic potential. Dual-activity motifs (Supplementary Figure S12G–I) combine both features—high cationic charge with substantial hydrophobic content—providing a motif-level explanation for the mechanistic coupling between antibacterial efficacy and hemolytic liability observed in the overlap analyses.

To avoid relying on a single visualization statistic, we report each motif class using three complementary logo encodings: frequency-based logos (left column) summarize positional residue usage among motif instances; information-content logos (middle column) emphasize conserved positions; and enrichment-weighted logos (right column) weight residues by enrichment strength, highlighting positions most associated with the target activity. Together, these motif logos translate the enrichment maps into interpretable residue-level rules and clarify how antibacterial activity, hemolysis, and their overlap emerge from partially shared—but non-identical—sequence patterns.

### R3.5 Motif-guided candidate prioritization

To illustrate downstream candidate prioritization, we implemented a proof-of-concept motif-guided generation-and-screening workflow to produce a compact panel of computationally prioritized peptide candidates. Specifically, explicit sequence design rules were distilled from the 7-mer enrichment analysis to favor antibacterial-associated motifs while avoiding patterns more strongly linked to hemolysis. Guided by these motif-derived constraints, candidate sequences were generated under built-in novelty constraints with respect to the benchmark and independent validation datasets, excluding exact duplicates as well as near-duplicate sequences with Levenshtein distance ≤ 2. The resulting virtual peptides were first screened by the MAPLE AMP-identification module and then prioritized using category-specific antibacterial and hemolysis predictions, yielding a final shortlist of fifteen peptides (Supplementary Table S11).

The selected candidates span 18–33 residues and exhibit canonical physicochemical signatures of membrane-active antimicrobial peptides, including net positive charge (3–10) and predominantly negative mean hydrophobicity, consistent with hydrophilic to mildly amphipathic profiles (Supplementary Table S12). For practical experimental planning, each sequence is reported with predicted antibacterial and hemolytic probabilities, together with a simple selectivity ratio summarizing predicted efficacy relative to predicted hemolysis.

Importantly, subsequent analyses were performed for independent characterization and external tool-based cross-evaluation without influencing the original MAPLE-based prioritization. First, we quantified conformational and membrane-interaction properties using the DBAASP property calculator, including hydrophobic moment, amphiphilicity, aggregation propensity, and secondary-structure tendencies (Supplementary Table S12). Second, developability- and safety-related attributes—including intrinsic solubility (CamSol)(68), aggregation propensity (AGGRESCAN)(69), hemolytic potential (HemoPI-2)(62), and general toxicity (ToxinPred)(70)—were evaluated to assess translational feasibility (Supplementary Table S13).

Finally, the functional plausibility of the prioritized candidates was further assessed using strain-specific activity predictors implemented in DBAASP (Supplementary Table S14) and consensus AMP classifiers provided by CAMPR4 under both natural- and synthetic-peptide training regimes (Supplementary Table S15)(3, 6, 71). Together, these external tool-based analyses provide orthogonal computational support for the predicted antibacterial potential, safety-related profile, and developability of the prioritized candidates, without influencing the original selection process.

**Supplementary Tables**

**Supplementary Table S1. Overview of AMP databases and functional activity coverage across 14 categories.**

|  |  | APD3 | dbAMP3 | CAMP R4 | DRAMP | AMPDB | DBAASP | DCTPep | DRAVP | AntiFP |
| --- | --- | --- | --- | --- | --- | --- | --- | --- | --- | --- |
| Stage-1 | AMP | 6,419 | 21,920 | 24,243 | 31,137 | 59,122 | 784 | 5,368 | 3,191 | 5,775 |
| Stage-2 | Anti-mammalian cells | 0 | 7,264 | 1,360 | 2,493 | 0 | 156 | 246 | 427 | 0 |
|  | Antibacterial | 4,729 | 7,625 | 22,328 | 30,757 | 49,399 | 442 | 1,854 | 5 | 0 |
|  | Antibiofilm | 174 | 40 | 76 | 18 | 51 | 5 | 0 | 0 | 0 |
|  | Anticancer | 325 | 68 | 1,385 | 4,670 | 51 | 39 | 5,368 | 370 | 0 |
|  | Antifungal | 1,623 | 5,708 | 10,572 | 3,507 | 4,391 | 76 | 721 | 3 | 5,775 |
|  | Antigram-negative | 5,541 | 12,191 | 11,908 | 2,942 | 5,800 | 277 | 0 | 0 | 0 |
|  | Antigram-positive | 5,607 | 11,779 | 10,954 | 3,120 | 2,238 | 383 | 0 | 0 | 0 |
|  | Anti-HIV | 145 | 1,024 | 489 | 756 | 46 | 23 | 0 | 1,852 | 0 |
|  | Anti-MRSA | 630 | 114 | 1,087 | 299 | 80 | 0 | 0 | 0 | 0 |
|  | Antioxidant | 34 | 30 | 98 | 348 | 356 | 82 | 3 | 0 | 0 |
|  | Antiparasitic | 141 | 417 | 228 | 258 | 100 | 1 | 33 | 1 | 0 |
|  | Antiviral | 349 | 2,018 | 1,890 | 3,462 | 33 | 189 | 103 | 3,191 | 0 |
|  | Cytotoxic | 0 | 3,349 | 707 | 1,508 | 2,727 | 152 | 125 | 385 | 0 |
|  | Hemolytic | 351 | 21 | 2,913 | 370 | 867 | 7 | 66 | 56 | 0 |

This table summarizes the number of antimicrobial peptide (AMP) sequences collected from nine curated databases. Stage-1 indicates the number of sequences identified as AMPs in each database. Stage-2 reports the number of AMPs further annotated with experimentally validated activity for each of the 14 functional categories. Each column represents a specific database. Zero values indicate no available annotations for the corresponding category in that database.

**Supplementary Table S2. Number of positive and negative samples for each functional label in the original iAMPCN dataset and benchmark dataset.**

|  |  | **Original iAMPCN dataset** | | | **Benchmark dataset** | | |
| --- | --- | --- | --- | --- | --- | --- | --- |
| Category |  | Positive samples | Negative samples | Positive % | Positive samples | Negative samples | Positive % |
| Stage-1 | AMP | 25,420 | 72,756 | 25.89% | 25,507 | 72,606 | 26.00% |
| Stage-2 | Anti-mammalian cells | 3,681 | 19,108 | 16.15% | 6,733 | 16,427 | 29.07% |
|  | Antibacterial | 12,046 | 10,569 | 53.27% | 18,970 | 5,425 | 77.76% |
|  | Antibiofilm | 316 | 22,059 | 1.41% | 387 | 22,002 | 1.73% |
|  | Anticancer | 4,201 | 18,605 | 18.42% | 5,657 | 17,317 | 24.62% |
|  | Antifungal | 5,117 | 17,717 | 22.41% | 10,379 | 13,085 | 44.23% |
|  | Antigram-negative | 7,040 | 16,023 | 30.53% | 10,870 | 12,884 | 45.76% |
|  | Antigram-positive | 6,679 | 16,414 | 28.92% | 10,475 | 13,281 | 44.09% |
|  | Anti-HIV | 671 | 21,850 | 2.98% | 1,718 | 20,997 | 7.56% |
|  | Anti-MRSA | 255 | 22,221 | 1.13% | 897 | 21,663 | 3.98% |
|  | Antioxidant | 50 | 22,388 | 0.22% | 428 | 22,057 | 1.90% |
|  | Antiparasitic | 404 | 22,111 | 1.79% | 660 | 21,891 | 2.93% |
|  | Antiviral | 4,811 | 18,013 | 21.08% | 5,481 | 17,508 | 23.84% |
|  | Cytotoxic | 170 | 22,234 | 0.76% | 3,126 | 19,590 | 13.76% |
|  | Hemolytic | 2,119 | 20,527 | 9.36% | 3,787 | 19,009 | 16.61% |

Number and proportion of positive and negative samples for each functional category in both the original iAMPCN dataset and the refined benchmark dataset. The benchmark dataset was constructed by supplementing the iAMPCN dataset with functional annotations integrated from nine curated AMP databases (see Supplementary Table S1). Stage-1 refers to AMP versus non-AMP classification. Stage-2 refers to the classification of AMP sequences across 14 functional categories. For each category, AMP sequences annotated with the corresponding activity were treated as positive samples, while the remaining AMP sequences were treated as negative. “Positive %” indicates the proportion of positive samples among all AMP sequences for each function.

**Supplementary Table S3. Complete breakdown of knowledge-based feature dimensions**

| **Feature Category** | **Dimensions** | **Description** |
| --- | --- | --- |
| Amino acid identity (one-hot) | 20 | Standard one-hot encoding of the 20 canonical amino acids |
| Physicochemical properties | 5 | Hydrophobicity (Kyte-Doolittle), charge, normalized molecular weight, α-helix propensity, and binary aromaticity |
| Sliding window features | 15 | Proportions of 8 functional residue groups within a 5-residue window plus 7 derived structural indicators (mean hydrophobicity, mean charge, complexity, charge/hydrophobic/amphipathic/aromatic clusters) |
| Positional encoding | 6 | Normalized position, N-/C-terminal and central region indicators, distance to nearest terminus, and sinusoidal encoding |
| Global sequence properties | 10 | Normalized length, net charge density, average hydrophobicity, estimated isoelectric point, normalized molecular weight, residue diversity, ratios of positive/hydrophobic/aromatic residues, and normalized Boman index (broadcast to all positions) |
| Subtotal | 56 | Complete per-residue knowledge-based feature vector |

**Supplementary Table S4. Comparative Summary of AMP Prediction Models.**

| **Method Name** | **Core Algorithm** | **Key Features** | **Application Scope** | **Interpretability** |
| --- | --- | --- | --- | --- |
| **MAPLE (Ours)** | PLM + Multi-Scale Fusion | 1. ESM-2 Protein Language Embeddings  2. Knowledge-Enhanced Feature Integration | AMP binary classification, Function Prediction | High, with interpretable attention and residue-level visualization |
| **AMP identification models** | | | | |
| **PLAPD** | PLM | 1. Pretrained ESM2 embeddings (1280-d)  2. Local-global fusion: CNN for local patterns, residual Transformer for global context  3. High-throughput binary AMP prediction with top precision | AMP binary classification | Low to Moderate: includes t-SNE feature projection, but lacks interpretability analysis or attention visualization |
| **AMPpred-MFA** | BiLSTM + CNN + Multihead Attention (stacked) | 1. Dual-branch feature extraction (fragment-level + dipeptide-level)  2. Multihead attention for subspace learning and motif highlighting | AMP binary classification | High: provides residue-level interpretability through multi-head attention visualization and motif discovery; includes multi-view explanation with attention heatmaps and t-SNE projection |
| **PepNet** | ProtT5-XL-U50 + Residual Dilated CNN + Residual Transformer | 1. Combines local dilated CNN and global residual Transformer  2. Uses 1024-d ProtT5 embeddings  3. Supports dual-task AMP/AIP prediction | AMP and AIP binary classification | Moderate: supports interpretation via t-SNE projection, physicochemical property profiling, and ablation, but lacks residue-level attribution or attention visualization. |
| **iAMP-Attenpred** | BERT + CNN + BiLSTM + Attention | 1. First AMP predictor combining BERT with CNN-BiLSTM-Attention  2. BERT encodes amino acids as contextualized tokens | AMP binary classification | Moderate to High: includes residue-level attention heatmaps from the CNN–BiLSTM–Attention architecture; interpretation remains model-internal and lacks biological alignment or post hoc attribution techniques |
| **ProteinBERT** | BERT-based Transformer | 1. Joint pretraining on sequence reconstruction and GO annotation prediction  2. Dual-path architecture with local (conv) + global (transformer) features  3. Scalable to variable-length sequences, trained on 106M UniRef90 proteins | General protein sequence/function prediction (used for AMP detection in downstream tasks) | Low to Moderate: attention-based architecture, but no interpretability analysis or attribution visualization provided, especially in AMP-specific tasks |
| **AI4AMP** | PC6 physicochemical encoding + CNN + LSTM | 1. PC6 encoding captures 6 optimal physicochemical properties  2. One-hot-like 200×6 matrix per sequence  3. CNN-LSTM classifier without pooling to preserve residue-level information | AMP binary classification | Low to Moderate: interpretable via manually selected input features; neural layers are black-box and lack attribution mechanisms |
| **AMP function prediction models** | | | | |
| **BioPeptide-pred** | Word Embedding + CNN + BiGRU | 1. Natural language-inspired embedding of amino acid sequences  2. CNN + BiGRU ensemble to capture local/global dependencies | Prediction of diverse bioactive peptides | High: Supports motif-level interpretation via multi-tier UMAP embedding projection and KpLogo-based residue analysis. |
| **UniDL4BioPep** | PLM + CNN | 1. Uses pretrained ESM-2 embeddings  2. Unified CNN-based predictor | Multi-bioactivity peptide prediction | Moderate: Provides global embedding visualization (UMAP), but lacks residue-level or mechanistic attribution. |
| **TransImbAMP** | TAPE Transformer + Asymmetric Loss | 1. Fine-tuned transformer pretrained on Pfam (TAPE)  2. Supports both AMP identification and multi-label function prediction  3. Adopts ASL to alleviate severe class imbalance | AMP binary classification and 7-function multi-label prediction | Low to Moderate: offers global UMAP visualization but lacks residue-level attribution or biological interpretation. |
| **iAMPCN** | CNN-based dual-stage framework | 1. Four parallel encodings (One-hot, BLOSUM62, AAIndex, PAAC)  2. Multi-branch CNNs with varying filter sizes (2–6)  3. Two-stage design: AMP identification + 22-label functional classification  4. Transfer learning and focal loss for imbalance handling | AMP binary classification and 22-function multi-label prediction | High: interpretable via residue-level SHAP analysis across six AMP functions, supported by mechanistic insights into amino acid contributions. |
| **iAMP-CA2L** | CA image encoding + CNN + BiLSTM + SVM | 1. Transforms sequences into 2D binary images via cellular automata  2. Extracts spatial–temporal features with CNN-BiLSTM  3. Stage-2 SVM classifier for multi-label prediction | Multi-label AMP functional classification | Moderate: partially interpretable via CA-based image feature maps and functional motif visualization, but lacks residue-level attribution or mechanistic explanation. |
| **deep-AMPpred** | ESM-2 + CNN + BiLSTM + CBAM | 1. Uses ESM-2 embeddings to encode contextual information  2. CNN for local pattern extraction; BiLSTM for sequence modeling  3. CBAM module enhances spatial-channel feature selection | Multi-label AMP functional classification | Low: no interpretability analysis was conducted despite using attention modules (CBAM); no visualization or attribution of model decisions provided. |
| **Hemolytic function prediction models** | | | | |
| **HemoFuse** | Word Embedding + Handcrafted Features + Bi-GRU + CNN + Multi-head Cross-Attention | 1. Fuses semantic (Transformer-based embedding) and statistical (BLOSUM62, DDE, DPC, CKSAAP) features  2. Bi-GRU aligns handcrafted features to embedding dimension  3. Multi-head cross-attention enables deep interaction between modalities  4. CNN + MLP for classification | Hemolytic peptide identification (binary classification) | Moderate: t-SNE visualization shows feature-level separation, but no residue-level attribution or mechanistic interpretability provided. |
| **HemoPI2** | Hybrid model (Random Forest + MERCI motif mining + PLMs) | 1. Classification + regression for hemolytic activity  2. Uses handcrafted features (AAC, DPC, PCP) and PLM embeddings (e.g., ESM2, ProtBERT)  3. Motif-aware hybrid architecture with MERCI integration  4. Supports regression on HC50 (hemolytic concentration) | Binary classification (hemolytic vs non-hemolytic) and regression (HC50 prediction) | Moderate to High: motif discovery (via MERCI), residue-level composition, positional and physicochemical pattern analysis; lacks token-level visualization for PLM-based models. |

**Note:** This table summarizes representative models for antimicrobial peptide (AMP) and functional peptide prediction in terms of their core algorithm, key technical features, application scope, and interpretability. Abbreviations used in the table are defined below. Interpretability levels are qualitatively assessed based on whether models provide residue-level attribution, global feature visualization, or no explanation of prediction mechanisms. **Abbreviations:** PLM: Pretrained Language Model; CNN: Convolutional Neural Network; BiLSTM: Bidirectional Long Short-Term Memory; BiGRU: Bidirectional Gated Recurrent Unit; MLP: Multi-Layer Perceptron; SVM: Support Vector Machine; CBAM: Convolutional Block Attention Module; CA: Cellular Automata; BERT: Bidirectional Encoder Representations from Transformers; ESM-2: Evolutionary Scale Modeling v2; ProtT5: Protein T5 Transformer; TAPE: Tasks Assessing Protein Embeddings; PC6: Physicochemical Encoding (6 properties); AAC: Amino Acid Composition; DPC: Dipeptide Composition; DDE: Dipeptide Deviation from Expected Mean; AAIndex: Amino Acid Index; CKSAAP: Composition of k-Spaced Amino Acid Pairs; PAAC: Pseudo Amino Acid Composition; BLOSUM62: Block Substitution Matrix 62; t-SNE: t-distributed Stochastic Neighbor Embedding; UMAP: Uniform Manifold Approximation and Projection; SHAP: SHapley Additive exPlanations; MERCI: Motif-EmeRging and with Classes-Identification; AMP: Antimicrobial Peptide; AIP: Anti-inflammatory Peptide; GO: Gene Ontology; HC50: 50% Hemolytic Concentration; ASL: Asymmetric Loss. **Interpretability levels:** High indicates that the model offers residue-level or motif-based attribution and provides biological insights into prediction mechanisms. Moderate refers to models that support global visualization techniques such as UMAP or t-SNE, but lack fine-grained biological explanations. Low indicates that no interpretability or attribution analysis is available.

**Supplementary Table S5. Performance comparison of different models for AMP discrimination on Benchmark dataset.**

| **Model** | **Accuracy** | **Precision** | **Sensitivity** | **Specificity** | **F1 Score** | **MCC** |
| --- | --- | --- | --- | --- | --- | --- |
| **AI4AMP** | 0.5987 (0.5960–0.6017) | 0.3475 (0.3424–0.3521) | 0.6194 (0.6135–0.6262) | 0.5914 (0.5875–0.5950) | 0.4452 (0.4400–0.4498) | 0.1854 (0.1798–0.1914) |
| **AMPpred_MFA** | 0.9404 (0.9391–0.9421) | 0.9035 (0.9004–0.9067) | 0.8627 (0.8589–0.8666) | 0.9676 (0.9664–0.9687) | 0.8826 (0.8799–0.8852) | 0.8431 (0.8397–0.8468) |
| **BioPeptide-pred** | 0.5774 (0.5741–0.5800) | 0.3350 (0.3301–0.3379) | 0.7085 (0.7014–0.7147) | 0.5339 (0.5310–0.5367) | 0.4549 (0.4492–0.4585) | 0.2099 (0.2029–0.2146) |
| **PLAPD** | 0.9618 (0.9604–0.9631) | 0.9751 (0.9733–0.9770) | 0.8755 (0.8704–0.8795) | 0.9921 (0.9916–0.9928) | 0.9226 (0.9197–0.9247) | 0.8997 (0.8961–0.9025) |
| **Pepnet** | 0.8968 (0.8947–0.8985) | 0.9235 (0.9203–0.9265) | 0.6574 (0.6516–0.6622) | 0.9809 (0.9800–0.9816) | 0.7680 (0.7637–0.7720) | 0.7209 (0.7163–0.7249) |
| **ProteinBERT** | 0.7416 (0.7383–0.7452) | 0.5259 (0.5079–0.5413) | 0.0616 (0.0586–0.0640) | 0.9805 (0.9798–0.9815) | 0.1103 (0.1051–0.1142) | 0.1075 (0.0992–0.1135) |
| **TransImbAMP** | 0.9446 (0.9435–0.9460) | 0.9715 (0.9698–0.9735) | 0.8008 (0.7959–0.8065) | 0.9922 (0.9918–0.9927) | 0.8779 (0.8749–0.8812) | 0.8491 (0.8459–0.8528) |
| **UniDL4BioPep** | 0.9704 (0.9693–0.9713) | 0.9680 (0.9664–0.9701) | 0.9112 (0.9071–0.9142) | 0.9900 (0.9896–0.9907) | 0.9387 (0.9362–0.9405) | 0.9199 (0.9170–0.9223) |
| **iAMP-Attenpred** | 0.5395 (0.5362–0.5429) | 0.2991 (0.2949–0.3032) | 0.5745 (0.5692–0.5786) | 0.5272 (0.5241–0.5312) | 0.3934 (0.3889–0.3985) | 0.0892 (0.0831–0.0953) |
| **iAMP-CA2L** | 0.7820 (0.7801–0.7848) | 0.7881 (0.7799–0.8006) | 0.1701 (0.1654–0.1752) | 0.9848 (0.9839–0.9859) | 0.2798 (0.2734–0.2867) | 0.2972 (0.2905–0.3027) |
| **iAMPCN** | 0.9766 (0.9759–0.9778) | **0.9880 (0.9868–0.9891)** | 0.9174 (0.9150–0.9210) | **0.9963 (0.9959–0.9966)** | 0.9514 (0.9500–0.9537) | 0.9371 (0.9354–0.9401) |
| **MAPLE** | **0.9851 (0.9842–0.9860)** | 0.9845 (0.9832–0.9862) | **0.9577 (0.9550–0.9604)** | 0.9947 (0.9942–0.9952) | **0.9709 (0.9691–0.9725)** | **0.9610 (0.9587–0.9632)** |

**Note:** F1: F1 Score, the harmonic mean of Precision and Sensitivity; MCC: Matthews Correlation Coefficient.

**Supplementary Table S6. Performance comparison of different models for AMP discrimination on independent validation dataset.**

| **Model** | **Accuracy** | **Precision** | **Sensitivity** | **Specificity** | **F1 Score** | **MCC** |
| --- | --- | --- | --- | --- | --- | --- |
| **AI4AMP** | 0.5259 (0.5207–0.5297) | 0.5363 (0.5296–0.5421) | 0.4226 (0.4170–0.4280) | 0.6303 (0.6233–0.6364) | 0.4727 (0.4665–0.4777) | 0.0541 (0.0444–0.0616) |
| **AMPpred_MFA** | 0.8125 (0.8094–0.8152) | 0.9190 (0.9150–0.9229) | 0.6877 (0.6829–0.6917) | 0.9387 (0.9356–0.9419) | 0.7867 (0.7833–0.7901) | 0.6465 (0.6405–0.6521) |
| **BioPeptide-pred** | 0.5666 (0.5630–0.5706) | 0.5701 (0.5661–0.5755) | 0.5614 (0.5562–0.5682) | 0.5718 (0.5667–0.5773) | 0.5657 (0.5620–0.5708) | 0.1332 (0.1262–0.1412) |
| **PLAPD** | 0.8130 (0.8091–0.8171) | **0.9567 (0.9528–0.9594)** | 0.6580 (0.6519–0.6654) | 0.9698 (0.9674–0.9717) | 0.7797 (0.7747–0.7850) | 0.6600 (0.6529–0.6664) |
| **Pepnet** | 0.6676 (0.6642–0.6717) | 0.9447 (0.9406–0.9493) | 0.3600 (0.3552–0.3661) | **0.9787 (0.9772–0.9806)** | 0.5214 (0.5158–0.5283) | 0.4303 (0.4243–0.4365) |
| **ProteinBERT** | 0.5019 (0.4983–0.5068) | 0.5024 (0.4988–0.5074) | 0.9851 (0.9836–0.9861) | 0.0131 (0.0119–0.0146) | 0.6654 (0.6623–0.6699) | −0.0077 (−0.0169–−0.0014) |
| **TransImbAMP** | 0.7785 (0.7753–0.7820) | 0.9102 (0.9062–0.9155) | 0.6207 (0.6161–0.6253) | 0.9380 (0.9354–0.9414) | 0.7381 (0.7341–0.7422) | 0.5885 (0.5826–0.5951) |
| **UniDL4BioPep** | 0.7886 (0.7848–0.7918) | 0.8941 (0.8911–0.8974) | 0.6575 (0.6505–0.6611) | 0.9212 (0.9185–0.9237) | 0.7577 (0.7526–0.7614) | 0.5994 (0.5925–0.6046) |
| **iAMP-Attenpred** | 0.7001 (0.6969–0.7038) | 0.7568 (0.7509–0.7633) | 0.5946 (0.5889–0.5994) | 0.8068 (0.8027–0.8106) | 0.6660 (0.6617–0.6709) | 0.4105 (0.4038–0.4172) |
| **iAMP-CA2L** | 0.4935 (0.4902–0.4972) | 0.4980 (0.4936–0.5023) | **0.8793 (0.8758–0.8830)** | 0.1033 (0.0999–0.1073) | 0.6358 (0.6318–0.6393) | −0.0276 (−0.0340–−0.0192) |
| **iAMPCN** | 0.8344 (0.8311–0.8374) | 0.9427 (0.9401–0.9459) | 0.7140 (0.7087–0.7202) | 0.9561 (0.9537–0.9585) | 0.8126 (0.8089–0.8166) | 0.6900 (0.6844–0.6950) |
| **MAPLE** | **0.8650 (0.8621–0.8676)** | 0.8600 (0.8555–0.8632) | 0.8717 (0.8682–0.8752) | 0.8583 (0.8542–0.8616) | **0.8658 (0.8624–0.8681)** | **0.7301 (0.7244–0.7352)** |

**Note:** F1: F1 Score, the harmonic mean of Precision and Sensitivity; MCC: Matthews Correlation Coefficient.

**Supplementary Table S7. Comparison of macro-averaged performance metrics for different AMP function prediction models on the benchmark dataset**.

| **Model** | **Macro-F1** | **Macro-Precision** | **Macro-Sensitivity** | **Macro-AUROC** |
| --- | --- | --- | --- | --- |
| **iAMPCN** | 0.5111 (0.5058, 0.5170) | 0.9241 (0.9144, 0.9324) | 0.4018 (0.3974, 0.4067) | 0.8544 (0.8520, 0.8574) |
| **TransImbAMP** | 0.3800 (0.3731, 0.3863) | 0.5107 (0.4989, 0.5242) | 0.3526 (0.3468, 0.3576) | 0.6660 (0.6615, 0.6700) |
| **deep_AMPpred** | 0.6653 (0.6574, 0.6711) | **0.9399 (0.9353, 0.9440)** | 0.5543 (0.5462, 0.5597) | 0.8803 (0.8775, 0.8827) |
| **iAMP-CA2L** | 0.1091 (0.1069, 0.1111) | 0.6462 (0.6063, 0.6965) | 0.0728 (0.0715, 0.0739) | 0.6810 (0.6757, 0.6862) |
| **BioPeptide-pred** | 0.3202 (0.3173, 0.3230) | 0.2883 (0.2855, 0.2910) | 0.4773 (0.4648, 0.4964) | 0.5451 (0.5369, 0.5594) |
| **UniDL4BioPep** | 0.2974 (0.2947, 0.3004) | 0.2282 (0.2262, 0.2306) | 0.7018 (0.6919, 0.7090) | 0.6000 (0.5916, 0.6070) |
| **MAPLE** | **0.7644 (0.7580, 0.7701)** | 0.6552 (0.6468, 0.6622) | **0.9365 (0.9338, 0.9390)** | **0.9721 (0.9714, 0.9729)** |

Note: F1: F1 Score, AUROC: Area Under the Receiver Operating Characteristic Curve.

**Supplementary Table S8. Performance comparison of different models across 14 functional peptide activity prediction tasks.**

A, anti-mammalian cells; B, antibacterial; C, antibiofilm; D, anticancer; E, antifungal; F, antigram-negative; G, antigram-positive; H, anti-HIV; I, anti-MRSA; J, antioxidant; K, antiparasitic; L, antiviral; M, cytotoxic; N, hemolytic. Values are reported for accuracy, sensitivity, specificity, precision, F1 score, Matthews correlation coefficient (MCC), and area under the precision-recall curve (AUPRC). The highest value in each metric is shown in bold.

A. anti-mammalian cells

| **Model** | **AUROC** | **AUPRC** | **Accuracy** | **Precision** | **Sensitivity** | **Specificity** | **F1** | **MCC** |
| --- | --- | --- | --- | --- | --- | --- | --- | --- |
| TransImbAMP | 0.6088 (0.6007-0.6175) | 0.4213 (0.4097-0.4332) | 0.6543 (0.6483-0.6604) | 0.4159 (0.4049-0.4266) | 0.4674 (0.4564-0.4798) | 0.7310 (0.7241-0.7373) | 0.4402 (0.4303-0.4506) | 0.1921 (0.1793-0.2054) |
| deep_AMPpred | 0.7237 (0.7174-0.7303) | 0.4670 (0.4558-0.4795) | 0.6375 (0.6314-0.6439) | 0.4305 (0.4224-0.4402) | 0.7649 (0.7549-0.7747) | 0.5853 (0.5774-0.5931) | 0.5509 (0.5431-0.5602) | 0.3182 (0.3070-0.3301) |
| iAMPCN | 0.8955 (0.8908-0.9005) | 0.8015 (0.7944-0.8089) | 0.8301 (0.8253-0.8355) | 0.6679 (0.6588-0.6782) | 0.8267 (0.8180-0.8357) | 0.8315 (0.8260-0.8375) | 0.7388 (0.7314-0.7470) | 0.6227 (0.6122-0.6341) |
| MAPLE | **0.9431 (0.9399-0.9459)** | **0.8745 (0.8675-0.8818)** | **0.8563 (0.8520-0.8606)** | **0.6919 (0.6830-0.7015)** | **0.9110 (0.9042-0.9178)** | **0.8337 (0.8280-0.8395)** | **0.7865 (0.7793-0.7938)** | **0.6958 (0.6864-0.7055)** |

B. antibacterial

| **Model** | **AUROC** | **AUPRC** | **Accuracy** | **Precision** | **Sensitivity** | **Specificity** | **F1** | **MCC** |
| --- | --- | --- | --- | --- | --- | --- | --- | --- |
| iAMP-CA2L | 0.5523 (0.5444-0.5610) | 0.8194 (0.8136-0.8257) | 0.4470 (0.4410-0.4531) | 0.8168 (0.8087-0.8252) | 0.3724 (0.3661-0.3789) | 0.7078 (0.6957-0.7201) | 0.5116 (0.5050-0.5187) | 0.0697 (0.0576-0.0819) |
| deep_AMPpred | 0.6341 (0.6266-0.6421) | 0.8571 (0.8515-0.8627) | 0.5561 (0.5501-0.5624) | 0.8582 (0.8522-0.8645) | 0.5141 (0.5076-0.5217) | 0.7030 (0.6912-0.7147) | 0.6430 (0.6372-0.6496) | 0.1810 (0.1697-0.1925) |
| BioPeptide-pred | 0.8008 (0.7946-0.8070) | 0.9227 (0.9185-0.9272) | 0.7723 (0.7674-0.7773) | 0.9071 (0.9027-0.9114) | 0.7879 (0.7824-0.7938) | 0.7179 (0.7062-0.7295) | 0.8433 (0.8395-0.8472) | 0.4492 (0.4375-0.4607) |
| UniDL4BioPep | 0.7988 (0.7924-0.8050) | 0.9186 (0.9146-0.9228) | 0.7816 (0.7765-0.7863) | 0.9100 (0.9058-0.9144) | 0.7981 (0.7925-0.8036) | 0.7238 (0.7126-0.7354) | 0.8504 (0.8466-0.8542) | 0.4660 (0.4552-0.4780) |
| iAMPCN | 0.8656 (0.8611-0.8702) | 0.9628 (0.9609-0.9647) | 0.7909 (0.7857-0.7961) | **0.9748 (0.9721-0.9773)** | 0.7505 (0.7444-0.7568) | **0.9322 (0.9252-0.9388)** | 0.8481 (0.8439-0.8524) | 0.5791 (0.5700-0.5885) |
| MAPLE | **0.9568 (0.9542-0.9596)** | **0.9864 (0.9853-0.9876)** | **0.8797 (0.8758-0.8838)** | 0.9720 (0.9696-0.9745) | **0.8704 (0.8653-0.8753)** | 0.9122 (0.9047-0.9195) | **0.9184 (0.9154-0.9214)** | **0.7078 (0.6990-0.7170)** |

C. antibiofilm

| **Model** | **AUROC** | **AUPRC** | **Accuracy** | **Precision** | **Sensitivity** | **Specificity** | **F1** | **MCC** |
| --- | --- | --- | --- | --- | --- | --- | --- | --- |
| deep_AMPpred | 0.7958 (0.7788-0.8133) | 0.0478 (0.0424-0.0539) | 0.7608 (0.7553-0.7662) | 0.0519 (0.0462-0.0579) | 0.7442 (0.7036-0.7864) | 0.7611 (0.7552-0.7665) | 0.0971 (0.0870-0.1078) | 0.1526 (0.1388-0.1677) |
| iAMP-CA2L | 0.8408 (0.8156-0.8644) | 0.3811 (0.3298-0.4350) | 0.8317 (0.8270-0.8368) | 0.0724 (0.0641-0.0807) | 0.7390 (0.6960-0.7800) | 0.8333 (0.8286-0.8385) | 0.1318 (0.1176-0.1457) | 0.1956 (0.1775-0.2129) |
| iAMPCN | 0.9307 (0.9145-0.9475) | 0.7130 (0.6682-0.7580) | 0.9644 (0.9620-0.9669) | 0.2965 (0.2689-0.3255) | 0.7700 (0.7287-0.8137) | 0.9679 (0.9655-0.9703) | 0.4282 (0.3959-0.4607) | 0.4645 (0.4339-0.4940) |
| MAPLE | **0.9986 (0.9981-0.9990)** | **0.9254 (0.9034-0.9449)** | **0.9841 (0.9825-0.9857)** | **0.5210 (0.4873-0.5571)** | **0.9922 (0.9832-1.0000)** | **0.9840 (0.9823-0.9856)** | **0.6833 (0.6529-0.7136)** | **0.7131 (0.6889-0.7386)** |

D. anticancer

| **Model** | **AUROC** | **AUPRC** | **Accuracy** | **Precision** | **Sensitivity** | **Specificity** | **F1** | **MCC** |
| --- | --- | --- | --- | --- | --- | --- | --- | --- |
| deep_AMPpred | 0.6259 (0.6177-0.6339) | 0.3466 (0.3348-0.3585) | 0.4319 (0.4250-0.4382) | 0.2857 (0.2786-0.2924) | 0.8711 (0.8622-0.8790) | 0.2883 (0.2814-0.2952) | 0.4302 (0.4219-0.4381) | 0.1589 (0.1476-0.1690) |
| TransImbAMP | 0.5873 (0.5792-0.5956) | 0.3239 (0.3124-0.3359) | 0.4739 (0.4674-0.4805) | 0.2821 (0.2743-0.2893) | 0.7354 (0.7243-0.7470) | 0.3885 (0.3806-0.3957) | 0.4077 (0.3985-0.4159) | 0.1114 (0.0989-0.1237) |
| UniDL4BioPep | 0.4988 (0.4898-0.5073) | 0.2632 (0.2549-0.2710) | 0.6907 (0.6844-0.6968) | 0.3034 (0.2883-0.3187) | 0.1977 (0.1871-0.2080) | 0.8517 (0.8463-0.8571) | 0.2394 (0.2273-0.2503) | 0.0580 (0.0447-0.0716) |
| BioPeptide-pred | 0.5575 (0.5486-0.5672) | 0.3249 (0.3132-0.3372) | 0.6854 (0.6794-0.6915) | 0.3562 (0.3429-0.3692) | 0.3439 (0.3316-0.3567) | 0.7970 (0.7908-0.8030) | 0.3499 (0.3384-0.3617) | 0.1426 (0.1290-0.1566) |
| iAMP-CA2L | 0.7193 (0.7115-0.7271) | 0.5327 (0.5195-0.5464) | 0.7038 (0.6979-0.7098) | 0.4242 (0.4131-0.4356) | 0.5676 (0.5546-0.5798) | 0.7483 (0.7415-0.7549) | 0.4856 (0.4753-0.4956) | 0.2896 (0.2765-0.3029) |
| iAMPCN | 0.8296 (0.8226-0.8366) | 0.7357 (0.7252-0.7458) | **0.8276 (0.8226-0.8323)** | **0.6393 (0.6272-0.6510)** | 0.6884 (0.6772-0.6997) | **0.8731 (0.8684-0.8779)** | 0.6629 (0.6530-0.6725) | 0.5480 (0.5357-0.5597) |
| MAPLE | **0.9279 (0.9244-0.9310)** | **0.8320 (0.8243-0.8398)** | 0.7811 (0.7759-0.7867) | 0.5315 (0.5223-0.5411) | **0.9367 (0.9302-0.9430)** | 0.7302 (0.7235-0.7373) | **0.6782 (0.6705-0.6868)** | **0.5798 (0.5707-0.5893)** |

E. antifungal

| **Model** | **AUROC** | **AUPRC** | **Accuracy** | **Precision** | **Sensitivity** | **Specificity** | **F1** | **MCC** |
| --- | --- | --- | --- | --- | --- | --- | --- | --- |
| BioPeptide-pred | 0.6677 (0.6606-0.6745) | 0.5958 (0.5859-0.6058) | 0.6187 (0.6122-0.6249) | 0.5565 (0.5474-0.5651) | 0.6796 (0.6700-0.6886) | 0.5703 (0.5615-0.5791) | 0.6120 (0.6043-0.6196) | 0.2491 (0.2363-0.2618) |
| deep_AMPpred | 0.6569 (0.6501-0.6640) | 0.6364 (0.6278-0.6456) | 0.6246 (0.6182-0.6304) | 0.5917 (0.5816-0.6022) | 0.4879 (0.4784-0.4972) | 0.7330 (0.7254-0.7401) | 0.5348 (0.5268-0.5436) | 0.2279 (0.2155-0.2401) |
| iAMP-CA2L | 0.6475 (0.6404-0.6545) | 0.6168 (0.6066-0.6266) | 0.6356 (0.6297-0.6419) | 0.6089 (0.5984-0.6192) | 0.4927 (0.4831-0.5022) | 0.7489 (0.7414-0.7561) | 0.5447 (0.5355-0.5529) | 0.2504 (0.2379-0.2629) |
| TransImbAMP | 0.6606 (0.6530-0.6683) | 0.6311 (0.6207-0.6419) | 0.6467 (0.6400-0.6527) | 0.6236 (0.6132-0.6338) | 0.5079 (0.4984-0.5181) | 0.7568 (0.7494-0.7641) | 0.5598 (0.5510-0.5690) | 0.2738 (0.2603-0.2865) |
| UniDL4BioPep | 0.7027 (0.6965-0.7093) | 0.5974 (0.5883-0.6072) | 0.6550 (0.6490-0.6612) | 0.5805 (0.5723-0.5886) | 0.7944 (0.7868-0.8025) | 0.5445 (0.5358-0.5534) | 0.6708 (0.6638-0.6773) | 0.3444 (0.3328-0.3565) |
| iAMPCN | 0.8237 (0.8180-0.8291) | 0.8259 (0.8200-0.8326) | 0.7646 (0.7592-0.7699) | 0.7986 (0.7900-0.8077) | 0.6257 (0.6169-0.6354) | **0.8748 (0.8689-0.8807)** | 0.7016 (0.6943-0.7097) | 0.5224 (0.5111-0.5332) |
| MAPLE | **0.9492 (0.9464-0.9518)** | **0.9359 (0.9320-0.9398)** | **0.8760 (0.8715-0.8801)** | **0.8399 (0.8326-0.8467)** | **0.8891 (0.8834-0.8948)** | 0.8656 (0.8596-0.8713) | **0.8638 (0.8588-0.8688)** | **0.7511 (0.7424-0.7596)** |

F. antigram-negative

| **Model** | **AUROC** | **AUPRC** | **Accuracy** | **Precision** | **Sensitivity** | **Specificity** | **F1** | **MCC** |
| --- | --- | --- | --- | --- | --- | --- | --- | --- |
| TransImbAMP | 0.7103 (0.7037-0.7170) | 0.6910 (0.6816-0.7003) | 0.6792 (0.6735-0.6851) | 0.6816 (0.6719-0.6904) | 0.5612 (0.5513-0.5705) | 0.7788 (0.7714-0.7858) | 0.6155 (0.6073-0.6229) | 0.3495 (0.3380-0.3608) |
| deep_AMPpred | 0.7329 (0.7269-0.7391) | 0.6559 (0.6467-0.6648) | 0.6668 (0.6610-0.6725) | 0.6086 (0.6008-0.6165) | 0.7615 (0.7539-0.7690) | 0.5869 (0.5791-0.5940) | 0.6765 (0.6701-0.6827) | 0.3508 (0.3394-0.3621) |
| iAMPCN | 0.9339 (0.9310-0.9371) | 0.9192 (0.9144-0.9237) | 0.8684 (0.8641-0.8727) | 0.8431 (0.8365-0.8495) | 0.8756 (0.8699-0.8810) | **0.8625 (0.8568-0.8681)** | 0.8590 (0.8541-0.8637) | 0.7363 (0.7279-0.7444) |
| MAPLE | **0.9550 (0.9524-0.9573)** | **0.9430 (0.9391-0.9466)** | **0.8851 (0.8811-0.8891)** | **0.8477 (0.8413-0.8539)** | **0.9133 (0.9085-0.9181)** | 0.8615 (0.8559-0.8670) | **0.8793 (0.8752-0.8832)** | **0.7721 (0.7644-0.7796)** |

G. antigram-positive

| **Model** | **AUROC** | **AUPRC** | **Accuracy** | **Precision** | **Sensitivity** | **Specificity** | **F1** | **MCC** |
| --- | --- | --- | --- | --- | --- | --- | --- | --- |
| TransImbAMP | 0.6842 (0.6773-0.6909) | 0.5926 (0.5835-0.6018) | 0.6561 (0.6503-0.6619) | 0.6113 (0.6020-0.6206) | 0.6048 (0.5953-0.6145) | 0.6967 (0.6892-0.7041) | 0.6080 (0.6005-0.6157) | 0.3017 (0.2904-0.3130) |
| deep_AMPpred | 0.7121 (0.7054-0.7179) | 0.6249 (0.6161-0.6336) | 0.6430 (0.6371-0.6488) | 0.5703 (0.5626-0.5778) | 0.7722 (0.7647-0.7797) | 0.5411 (0.5334-0.5486) | 0.6561 (0.6499-0.6624) | 0.3172 (0.3058-0.3284) |
| iAMPCN | 0.9319 (0.9287-0.9352) | 0.9135 (0.9082-0.9184) | **0.8655 (0.8611-0.8701)** | **0.8340 (0.8269-0.8407)** | 0.8656 (0.8597-0.8714) | **0.8641 (0.8581-0.8699)** | 0.8494 (0.8440-0.8544) | 0.7271 (0.7179-0.7357) |
| MAPLE | **0.9424 (0.9396-0.9450)** | **0.9234 (0.9185-0.9276)** | 0.8647 (0.8602-0.8689) | 0.8154 (0.8078-0.8214) | **0.8960 (0.8905-0.9017)** | 0.8400 (0.8334-0.8454) | **0.8538 (0.8486-0.8584)** | **0.7312 (0.7226-0.7396)** |

H. anti-HIV

| **Model** | **AUROC** | **AUPRC** | **Accuracy** | **Precision** | **Sensitivity** | **Specificity** | **F1** | **MCC** |
| --- | --- | --- | --- | --- | --- | --- | --- | --- |
| iAMP-CA2L | 0.7086 (0.6952-0.7228) | 0.3584 (0.3351-0.3816) | 0.8458 (0.8409-0.8501) | 0.2260 (0.2115-0.2398) | 0.4284 (0.4056-0.4493) | 0.8799 (0.8754-0.8841) | 0.2959 (0.2789-0.3112) | 0.2326 (0.2150-0.2496) |
| iAMPCN | 0.8902 (0.8807-0.8999) | 0.6748 (0.6517-0.6989) | 0.8858 (0.8815-0.8898) | 0.3737 (0.3559-0.3884) | 0.7538 (0.7342-0.7751) | 0.8966 (0.8923-0.9006) | 0.4997 (0.4817-0.5162) | 0.4783 (0.4609-0.4948) |
| MAPLE | **0.9592 (0.9538-0.9645)** | **0.8240 (0.8046-0.8426)** | **0.8979 (0.8938-0.9019)** | **0.4188 (0.4014-0.4368)** | **0.9021 (0.8843-0.9187)** | **0.8976 (0.8934-0.9017)** | **0.5721 (0.5547-0.5892)** | **0.5726 (0.5560-0.5892)** |

I. anti-MRSA

| **Model** | **AUROC** | **AUPRC** | **Accuracy** | **Precision** | **Sensitivity** | **Specificity** | **F1** | **MCC** |
| --- | --- | --- | --- | --- | --- | --- | --- | --- |
| UniDL4BioPep | 0.7174 (0.7021-0.7335) | 0.0801 (0.0712-0.0897) | 0.6487 (0.6424-0.6550) | 0.0742 (0.0676-0.0811) | 0.6823 (0.6510-0.7133) | 0.6473 (0.6410-0.6536) | 0.1338 (0.1213-0.1468) | 0.1337 (0.1172-0.1502) |
| iAMP-CA2L | 0.7060 (0.6854-0.7253) | 0.2705 (0.2396-0.3017) | 0.8000 (0.7954-0.8046) | 0.1003 (0.0916-0.1090) | 0.5072 (0.4736-0.5382) | 0.8116 (0.8059-0.8167) | 0.1675 (0.1542-0.1809) | 0.1554 (0.1381-0.1713) |
| iAMPCN | 0.7552 (0.7359-0.7732) | 0.3362 (0.3113-0.3607) | 0.7523 (0.7470-0.7575) | 0.0969 (0.0894-0.1048) | 0.6288 (0.5968-0.6596) | 0.7575 (0.7525-0.7624) | 0.1680 (0.1551-0.1815) | 0.1725 (0.1567-0.1883) |
| MAPLE | **0.9471 (0.9402-0.9536)** | **0.5511 (0.5208-0.5839)** | **0.8899 (0.8858-0.8940)** | **0.2508 (0.2363-0.2664)** | **0.8896 (0.8699-0.9085)** | **0.8899 (0.8858-0.8941)** | **0.3913 (0.3728-0.4109)** | **0.4376 (0.4207-0.4546)** |

J. antioxidant

| **Model** | **AUROC** | **AUPRC** | **Accuracy** | **Precision** | **Sensitivity** | **Specificity** | **F1** | **MCC** |
| --- | --- | --- | --- | --- | --- | --- | --- | --- |
| BioPeptide-pred | 0.5459 (0.5162-0.5726) | 0.0226 (0.0198-0.0263) | 0.5655 (0.5585-0.5719) | 0.0227 (0.0198-0.0257) | 0.5187 (0.4708-0.5662) | 0.5664 (0.5593-0.5728) | 0.0435 (0.0379-0.0491) | 0.0235 (0.0100-0.0366) |
| UniDL4BioPep | 0.5169 (0.4873-0.5486) | 0.0236 (0.0204-0.0275) | 0.8390 (0.8343-0.8438) | 0.0339 (0.0284-0.0395) | 0.2710 (0.2289-0.3114) | 0.8500 (0.8451-0.8547) | 0.0602 (0.0505-0.0700) | 0.0461 (0.0301-0.0613) |
| MAPLE | **0.9716 (0.9604-0.9810)** | **0.7314 (0.6913-0.7703)** | **0.9392 (0.9360-0.9423)** | **0.2292 (0.2094-0.2479)** | **0.9276 (0.9000-0.9524)** | **0.9395 (0.9363-0.9426)** | **0.3676 (0.3412-0.3917)** | **0.4443 (0.4218-0.4655)** |

K. antiparasitic

| **Model** | **AUROC** | **AUPRC** | **Accuracy** | **Precision** | **Sensitivity** | **Specificity** | **F1** | **MCC** |
| --- | --- | --- | --- | --- | --- | --- | --- | --- |
| UniDL4BioPep | 0.5570 (0.5377-0.5756) | 0.0325 (0.0295-0.0365) | 0.4512 (0.4448-0.4579) | 0.0360 (0.0328-0.0393) | 0.6894 (0.6549-0.7221) | 0.4440 (0.4373-0.4507) | 0.0685 (0.0623-0.0745) | 0.0453 (0.0332-0.0571) |
| TransImbAMP | 0.6927 (0.6710-0.7161) | 0.1044 (0.0871-0.1283) | 0.7075 (0.7012-0.7133) | 0.0579 (0.0524-0.0638) | 0.5894 (0.5501-0.6298) | 0.7110 (0.7048-0.7169) | 0.1055 (0.0960-0.1154) | 0.1107 (0.0965-0.1261) |
| iAMP-CA2L | 0.7621 (0.7400-0.7833) | 0.3275 (0.2909-0.3651) | 0.8528 (0.8482-0.8574) | 0.1086 (0.0983-0.1194) | 0.5591 (0.5216-0.5982) | 0.8616 (0.8572-0.8663) | 0.1819 (0.1661-0.1986) | 0.1982 (0.1795-0.2176) |
| iAMPCN | 0.8474 (0.8299-0.8650) | **0.4978 (0.4582-0.5358)** | 0.9178 (0.9140-0.9213) | 0.2053 (0.1873-0.2219) | 0.6303 (0.5947-0.6662) | 0.9265 (0.9229-0.9297) | 0.3098 (0.2867-0.3318) | 0.3282 (0.3038-0.3512) |
| MAPLE | **0.9386 (0.9314-0.9456)** | 0.3532 (0.3233-0.3887) | **0.9388 (0.9358-0.9418)** | **0.3065 (0.2899-0.3297)** | **0.8636 (0.8389-0.8881)** | **0.9411 (0.9381-0.9440)** | **0.4524 (0.4329-0.4784)** | **0.4931 (0.4748-0.5159)** |

L. antiviral

| **Model** | **AUROC** | **AUPRC** | **Accuracy** | **Precision** | **Sensitivity** | **Specificity** | **F1** | **MCC** |
| --- | --- | --- | --- | --- | --- | --- | --- | --- |
| UniDL4BioPep | 0.5075 (0.4990-0.5161) | 0.2389 (0.2318-0.2463) | 0.3450 (0.3389-0.3512) | 0.2464 (0.2399-0.2525) | 0.8489 (0.8392-0.8576) | 0.1872 (0.1815-0.1928) | 0.3820 (0.3739-0.3895) | 0.0402 (0.0279-0.0530) |
| BioPeptide-pred | 0.5846 (0.5762-0.5935) | 0.3080 (0.2975-0.3194) | 0.5947 (0.5887-0.6007) | 0.2931 (0.2845-0.3029) | 0.4959 (0.4816-0.5086) | 0.6256 (0.6189-0.6329) | 0.3685 (0.3587-0.3785) | 0.1055 (0.0929-0.1186) |
| TransImbAMP | 0.7191 (0.7107-0.7268) | 0.5134 (0.4998-0.5270) | 0.7118 (0.7062-0.7178) | 0.4225 (0.4111-0.4343) | 0.5698 (0.5561-0.5831) | 0.7562 (0.7501-0.7625) | 0.4852 (0.4743-0.4960) | 0.2974 (0.2841-0.3110) |
| iAMP-CA2L | 0.7260 (0.7177-0.7341) | 0.5378 (0.5248-0.5507) | 0.7300 (0.7245-0.7353) | 0.4451 (0.4339-0.4568) | 0.5373 (0.5244-0.5505) | 0.7903 (0.7845-0.7963) | 0.4869 (0.4763-0.4976) | 0.3084 (0.2953-0.3217) |
| deep_AMPpred | 0.7577 (0.7504-0.7647) | 0.5366 (0.5215-0.5502) | 0.6994 (0.6931-0.7048) | 0.4184 (0.4072-0.4286) | 0.6685 (0.6553-0.6804) | 0.7090 (0.7024-0.7155) | 0.5146 (0.5036-0.5239) | 0.3313 (0.3170-0.3432) |
| iAMPCN | 0.9236 (0.9188-0.9286) | **0.8860 (0.8795-0.8927)** | **0.9106 (0.9070-0.9144)** | **0.8091 (0.7988-0.8193)** | 0.8183 (0.8085-0.8276) | **0.9396 (0.9363-0.9431)** | **0.8137 (0.8061-0.8208)** | **0.7549 (0.7448-0.7642)** |
| MAPLE | **0.9326 (0.9288-0.9360)** | 0.8488 (0.8403-0.8565) | 0.8548 (0.8502-0.8592) | 0.6479 (0.6361-0.6589) | **0.8559 (0.8469-0.8647)** | 0.8544 (0.8494-0.8594) | 0.7375 (0.7284-0.7462) | 0.6516 (0.6410-0.6619) |

M. cytotoxic

| **Model** | **AUROC** | **AUPRC** | **Accuracy** | **Precision** | **Sensitivity** | **Specificity** | **F1** | **MCC** |
| --- | --- | --- | --- | --- | --- | --- | --- | --- |
| iAMPCN | 0.5907 (0.5792-0.6024) | 0.2413 (0.2279-0.2556) | 0.6200 (0.6136-0.6262) | 0.1829 (0.1751-0.1911) | 0.5080 (0.4907-0.5255) | 0.6379 (0.6311-0.6444) | 0.2690 (0.2585-0.2796) | 0.1034 (0.0896-0.1173) |
| MAPLE | **0.9201 (0.9152-0.9250)** | **0.7196 (0.7048-0.7348)** | **0.8171 (0.8118-0.8221)** | **0.4206 (0.4091-0.4330)** | **0.8724 (0.8607-0.8837)** | **0.8083 (0.8027-0.8133)** | **0.5676 (0.5564-0.5793)** | **0.5192 (0.5079-0.5310)** |

N. hemolytic

| **Model** | **AUROC** | **AUPRC** | **Accuracy** | **Precision** | **Sensitivity** | **Specificity** | **F1** | **MCC** |
| --- | --- | --- | --- | --- | --- | --- | --- | --- |
| HEMOPI2 | 0.7035 (0.6956-0.7112) | 0.2605 (0.2511-0.2709) | 0.5816 (0.5751-0.5879) | 0.2526 (0.2450-0.2609) | 0.7750 (0.7610-0.7876) | 0.5431 (0.5360-0.5500) | 0.3810 (0.3716-0.3914) | 0.2369 (0.2253-0.2491) |
| HemoFuse | 0.6064 (0.5976-0.6155) | 0.1912 (0.1850-0.1983) | 0.5188 (0.5117-0.5256) | 0.2198 (0.2131-0.2272) | 0.7439 (0.7306-0.7574) | 0.4740 (0.4667-0.4813) | 0.3394 (0.3305-0.3490) | 0.1634 (0.1514-0.1752) |
| iAMPCN | 0.8841 (0.8776-0.8903) | 0.7124 (0.6978-0.7252) | 0.8036 (0.7986-0.8084) | 0.4486 (0.4371-0.4605) | 0.7969 (0.7843-0.8090) | 0.8049 (0.7997-0.8103) | 0.5741 (0.5632-0.5851) | 0.4911 (0.4791-0.5030) |
| MAPLE | **0.9303 (0.9266-0.9342)** | **0.7163 (0.7020-0.7309)** | **0.8283 (0.8237-0.8337)** | **0.4910 (0.4796-0.5026)** | **0.9147 (0.9061-0.9233)** | **0.8111 (0.8058-0.8169)** | **0.6390 (0.6286-0.6498)** | **0.5844 (0.5737-0.5953)** |

**Supplementary Table S9. Performance comparison of different loss functions for the AMP Discrimination.**

| **Model** | **AUROC** | **Accuracy** | **Precision** | **Sensitivity** | **Specificity** | **F1 Score** | **MCC** |
| --- | --- | --- | --- | --- | --- | --- | --- |
| **BCE** | 0.9978 (0.9975–0.9981) | 0.9847 (0.9839–0.9855) | **0.9904 (0.9891–0.9916)** | 0.9476 (0.9447–0.9502) | **0.9969 (0.9965–0.9973)** | 0.9685 (0.9668–0.9701) | 0.9588 (0.9567–0.9607) |
| **Weighted BCE** | 0.9980 (0.9978–0.9983) | 0.9837 (0.9828–0.9844) | 0.9786 (0.9767–0.9803) | 0.9553 (0.9528–0.9578) | 0.9931 (0.9925–0.9937) | 0.9668 (0.9652–0.9685) | 0.9561 (0.9539–0.9581) |
| **Asymmetric loss** | 0.9967 (0.9965–0.9970) | 0.9782 (0.9773–0.9791) | 0.9614 (0.9588–0.9639) | 0.9506 (0.9478–0.9534) | 0.9873 (0.9866–0.9881) | 0.9559 (0.9541–0.9579) | 0.9415 (0.9391–0.9438) |
| **Poly loss** | 0.9960 (0.9958–0.9963) | 0.9771 (0.9762–0.9781) | 0.9673 (0.9650–0.9695) | 0.9398 (0.9366–0.9428) | 0.9895 (0.9888–0.9902) | 0.9534 (0.9516–0.9554) | 0.9384 (0.9359–0.9407) |
| **Focal loss** | **0.9989 (0.9988–0.9990)** | **0.9851 (0.9843–0.9858)** | 0.9845 (0.9830–0.9860) | **0.9577 (0.9552–0.9602)** | 0.9947 (0.9942–0.9952) | **0.9709 (0.9694–0.9724)** | **0.9611 (0.9590–0.9629)** |

**Note:** BCE, Binary Cross-Entropy Loss; AUROC: Area Under the Receiver Operating Characteristic Curve; F1: F1 Score, the harmonic mean of Precision and Sensitivity; MCC: Matthews Correlation Coefficient.

**Supplementary Table S10. Performance comparison of different loss functions for the AMP multi-label functional prediction.**

| **Model** | **Macro-Precision** | **Macro-Sensitivity** | **Macro-F1** | **Macro-AUROC** |
| --- | --- | --- | --- | --- |
| **BCE** | 0.6899 (0.6842–0.6956) | 0.7566 (0.7513–0.7619) | 0.7218 (0.7162–0.7274) | 0.9315 (0.9284–0.9346) |
| **Weighted BCE** | 0.6918 (0.6861–0.6975) | 0.9082 (0.9046–0.9118) | 0.7853 (0.7802–0.7904) | 0.9690 (0.9669–0.9711) |
| **Asymmetric loss** | 0.5932 (0.5871–0.5993) | 0.7964 (0.7914–0.8014) | 0.6799 (0.6741–0.6857) | 0.9075 (0.9039–0.9111) |
| **Poly loss** | **0.7935 (0.7885–0.7985)** | 0.8565 (0.8522–0.8608) | 0.8238 (0.8191–0.8285) | 0.9728 (0.9711–0.9751) |
| **Focal loss** | 0.7846 (0.7807–0.7885) | **0.9365 (0.9338–0.9390)** | **0.8538 (0.8494–0.8582)** | **0.9731 (0.9724–0.9739)** |

Note: BCE, Binary Cross-Entropy Loss; F1: F1 Score, the harmonic mean of Precision and Sensitivity; AUROC: Area Under the Receiver Operating Characteristic Curve.

**Supplementary Table S11.** Designed peptide candidates for experimental validation.

| **ID** | **Sequence** | Length | Net charge | Mean hydrophobicity | antibacterial | hemolytic | Selectivity ratio |
| --- | --- | --- | --- | --- | --- | --- | --- |
| P01 | PTGKIGIDKATQKATKFIPQWIRGDQR | 27 | 4 | -0.1896 | 0.7744 | 0.1384 | 5.597 |
| P02 | TTKAFAQRVEGAAIKTKKRLNHKSR | 25 | 7.5 | -0.4208 | 0.7644 | 0.0128 | 59.7247 |
| P03 | HNGRYAGRQFAVYHNRYR | 18 | 5 | -0.4489 | 0.7606 | 0.0992 | 7.6702 |
| P04 | LVTKTAGRKIEKYLGRRGQRAQGRQANRHKQN | 32 | 9.5 | -0.5862 | 0.7412 | 0.0956 | 7.754 |
| P05 | RSRSNSSGSTGAAPNHKQGNRPWNNRPGGGSQ | 32 | 5.5 | -0.4206 | 0.7394 | 0.0162 | 45.6211 |
| P06 | HKHYKKQGSRSRRARQAIKEEVRQALRYK | 29 | 10 | -0.741 | 0.7214 | 0.0286 | 25.1896 |
| P07 | GRKAFGVAKKHHSNQSKSGGHHHK | 24 | 8.5 | -0.3654 | 0.7197 | 0.0878 | 8.1981 |
| P08 | TIGGAATKATEFIKKPSDRR | 20 | 3 | -0.232 | 0.7192 | 0.1254 | 5.736 |
| P09 | RHYGQQQAPTRYKRDQPPDGRNNQRGNHRYKVY | 33 | 7 | -0.6933 | 0.7135 | 0.0174 | 41.0734 |
| P10 | NRSKGAAIGRGLDKARSAIRKRTSTKQKH | 29 | 9.5 | -0.551 | 0.7096 | 0.2763 | 2.5682 |
| P11 | SEYKTDKKQEANRGRGRFRGNNVGTHQVS | 29 | 4.5 | -0.5431 | 0.709 | 0.0107 | 66.5556 |
| P12 | KKRLAVKGADRIGKGIARAGKVKNGHR | 27 | 9.5 | -0.383 | 0.709 | 0.0415 | 17.0648 |
| P13 | GHGQGGTGQGAKYGGDRKPRNRG | 23 | 4.5 | -0.3957 | 0.7068 | 0.0095 | 74.0649 |
| P14 | GNTVQGVQKPGRMHKVQGRCQNRQGQNMRKRGQ | 33 | 8.5 | -0.5479 | 0.7061 | 0.046 | 15.3462 |
| P15 | KVAHSNNKTHVKEAHQKARRQKEKFGAQR | 29 | 8.5 | -0.5966 | 0.7053 | 0.0814 | 8.6638 |

**Note:** Fifteen computationally designed antimicrobial peptide candidates were generated using explicit design rules derived from k-mer enrichment analysis and prioritized using MAPLE’s multifunctional prediction profile. **Selectivity ratio** was defined as $\frac{P_{antibacterial}}{P_{hemolytic}}$. **Net charge** was computed as +1+1+1 for K/R, +0.5+0.5+0.5 for H, −1-1−1 for D/E, and 0 for other residues (sequence-summed). **Mean hydrophobicity** was calculated as the per-residue average using the **Eisenberg** hydrophobicity scale. Candidate sequences were filtered to exclude any exact matches to sequences in the benchmark or independent datasets, and additionally removed if the Levenshtein distance to any known sequence was ≤2.

**Supplementary Table S12.** Physicochemical and conformational properties of designed peptide candidates predicted by the DBAASP property calculator.

| **Parameter** | **P01** | **P02** | **P03** | **P04** | **P05** | **P06** | **P07** | **P08** | **P09** | **P10** | **P11** | **P12** | **P13** | **P14** | **P15** |
| --- | --- | --- | --- | --- | --- | --- | --- | --- | --- | --- | --- | --- | --- | --- | --- |
| Normalized Hydrophobic Moment | 0.69 | 0.73 | 0.72 | 0.39 | 0.16 | 0.72 | 0.58 | 0.96 | 0.29 | 0.42 | 0.39 | 0.48 | 0.08 | 0.53 | 0.34 |
| Normalized Hydrophobicity | 0.01 | 0.57 | 0.18 | 0.70 | 0.51 | 0.85 | 1.36 | 0.03 | 0.59 | 0.81 | 0.77 | 0.67 | 0.77 | 0.80 | 1.14 |
| Isoelectric Point | 11.14 | 11.95 | 11.04 | 12.14 | 12.58 | 11.64 | 11.81 | 10.90 | 10.88 | 12.31 | 10.91 | 12.18 | 11.32 | 12.30 | 11.56 |
| Tilt Angle | 116 | 34 | 95 | 36 | 81 | 136 | 41 | 17 | 125 | 73 | 120 | 96 | 82 | 88 | 102 |
| Disordered Conformation Propensity | -0.19 | -0.38 | -0.48 | -0.55 | -0.49 | -0.68 | -0.53 | -0.15 | -0.71 | -0.50 | -0.47 | -0.38 | -0.52 | -0.55 | -0.58 |
| Propensity to in vitro Aggregation | 0.005 | 0.003 | 3.288 | 0.001 | 0.002 | 0.003 | 0.002 | 0.008 | 0.002 | 0.003 | 0.002 | 0.003 | 0.002 | 0.002 | 0.004 |
| Amphiphilicity Index | 1.12 | 1.19 | 1.62 | 1.32 | 0.76 | 1.81 | 1.22 | 0.86 | 1.56 | 1.15 | 1.12 | 1.23 | 1.03 | 1.01 | 1.38 |
| Propensity to PPII coil | 1.01 | 0.95 | 0.97 | 0.90 | 1.06 | 0.97 | 0.93 | 0.99 | 1.08 | 0.95 | 0.93 | 0.86 | 0.82 | 0.94 | 0.96 |

Note: Properties were calculated using the DBAASP Property Calculation tool. Penetration Depth in Å; Tilt Angle in °. Values are as output by the tool (default or KD scale for hydrophobic-related parameters).

**Supplementary Table S13.** Developability, safety, and external platform profiling of designed peptide candidates.

| ID | CamSol (intrinsic solubility) | AGGRESCAN score | HemoPI2 (hemolysis) | ToxinPred (toxicity) |
| --- | --- | --- | --- | --- |
| P01 | 1.872 | -25.1 | Non-hemolytic | Non-toxin |
| P02 | 2.366 | -35.6 | Non-hemolytic | Non-toxin |
| P03 | 1.731 | -32.4 | Non-hemolytic | Non-toxin |
| P04 | 2.619 | -56.1 | Non-hemolytic | Non-toxin |
| P05 | 2.549 | -66.3 | Non-hemolytic | Non-toxin |
| P06 | 2.653 | -50.5 | Non-hemolytic | Non-toxin |
| P07 | 2.101 | -56.6 | Non-hemolytic | Toxin |
| P08 | 2.398 | -31.1 | Non-hemolytic | Non-toxin |
| P09 | 2.61 | -68.8 | Non-hemolytic | Non-toxin |
| P10 | 2.707 | -49.2 | Non-hemolytic | Non-toxin |
| P11 | 2.611 | -62.3 | Non-hemolytic | Non-toxin |
| P12 | 2.384 | -33.8 | Non-hemolytic | Non-toxin |
| P13 | 2.759 | -74.3 | Non-hemolytic | Non-toxin |
| P14 | 2.423 | -57.3 | Non-hemolytic | Non-toxin |
| P15 | 2.497 | -63.2 | Non-hemolytic | Non-toxin |

Note: CamSol intrinsic solubility scores was computed using the CamSol method; higher scores indicate higher predicted intrinsic solubility. Aggregation propensity was assessed using AGGRESCAN; the reported value corresponds to the normalized a4v sequence sum for 100 residues (Na4vSS), where more negative values indicate lower predicted aggregation propensity and fewer aggregation “hot spots” under the default threshold. Hemolytic risk was evaluated with HemoPI2 and reported as the model’s class output (Non-hemolytic/hemolytic) under the default settings. General peptide toxicity was predicted using ToxinPred and reported as the categorical output (Non-toxin/Toxin). All values are reported exactly as output by the respective tools (default parameters), and these in silico estimates should be interpreted as supportive developability and safety indicators rather than experimental measurements.

**Supplementary Table S14.** Strain-specific antibacterial, antiviral and hemolytic activity predictions of designed peptides using the DBAASP platform.

| **ID** | **DBAASP general AMP** | **Hemolysis prediction** | **Antibacterial activity** | **Antifungal activity** | **Antiviral activity** | **Active bacterial strains** | **Active fungal species** | **Active viruses** |
| --- | --- | --- | --- | --- | --- | --- | --- | --- |
| P01 | AMP | Non-hemolytic | Non-active | Non-active | Active | – | – | HIV-1; SARS-CoV-2; ZIKV |
| P02 | AMP | Non-hemolytic | Active | Non-active | Active | *A. baumannii* | *B. subtilis* | HIV-1; SARS-CoV-2; ZIKV |
| P03 | Non-AMP | Non-hemolytic | Active | Non-active | Active | *A. baumannii* | *B. subtilis* | SARS-CoV-2 |
| P04 | AMP | Non-hemolytic | Active | Non-active | Active | *E. coli*; *P. aeruginosa*; *K. pneumoniae*; *S. typhimurium*; *A. baumannii* | *B. subtilis* | HIV-1; SARS-CoV-2; ZIKV |
| P05 | Non-AMP | Non-hemolytic | Non-active | Non-active | Active | – | – | HIV-1; SARS-CoV-2; ZIKV |
| P06 | AMP | Non-hemolytic | Active | Active | Active | *E. coli*; *A. baumannii* | *C. albicans*; *S. cerevisiae* | HIV-1; SARS-CoV-2; ZIKV |
| P07 | Non-AMP | Non-hemolytic | Non-active | Non-active | Active | – | – | HIV-1; SARS-CoV-2; ZIKV |
| P08 | AMP | Non-hemolytic | Non-active | Non-active | Active | – | – | HIV-1; SARS-CoV-2; ZIKV |
| P09 | Non-AMP | Non-hemolytic | Active | Active | Active | *S. typhimurium*; *A. baumannii* | *C. albicans*; *S. cerevisiae* | HIV-1; SARS-CoV-2; ZIKV |
| P10 | AMP | Non-hemolytic | Active | Active | Active | *P. aeruginosa*; *A. baumannii* | *C. albicans*; *S. cerevisiae* | HIV-1; SARS-CoV-2; ZIKV |
| P11 | Non-AMP | Non-hemolytic | Active | Active | Active | *E. coli*; *P. aeruginosa*; *K. pneumoniae*; *S. typhimurium*; *A. baumannii* | *C. albicans*; *S. cerevisiae* | HIV-1; SARS-CoV-2; ZIKV |
| P12 | AMP | Non-hemolytic | Active | Non-active | Active | *P. aeruginosa*; *S. typhimurium*; *A. baumannii* | *B. subtilis* | HIV-1; SARS-CoV-2; ZIKV |
| P13 | Non-AMP | Non-hemolytic | Non-active | Non-active | Active | – | – | HIV-1; SARS-CoV-2; ZIKV |
| P14 | Non-AMP | Non-hemolytic | Active | Non-active | Active | *E. coli*; *P. aeruginosa*; *K. pneumoniae*; *S. typhimurium*; *A. baumannii* | *B. subtilis* | HIV-1; SARS-CoV-2; ZIKV |
| P15 | Non-AMP | Non-hemolytic | Active | Active | Active | *E. coli*; *A. baumannii* | *C. albicans*; *S. cerevisiae* | HIV-1; SARS-CoV-2; ZIKV |

Note: Activity predictions were obtained using the DBAASP platform. “Active” indicates predicted MIC < 25 μg/ml for bacterial strains, antifungal activity as defined by the tool, or IC50/EC50 < 50 μg/ml for viral entry inhibition. Hemolysis prediction was based on the strain-specific hemolytic activity model trained on DBAASP annotations.

**Supplementary Table S15.** External antimicrobial peptide prediction of designed candidates using the CAMPR4 platform.

| ID | Nat RF | Nat SVM | Nat ANN | Syn RF | Syn SVM | Syn ANN | Positive models | CAMPR4 consensus |
| --- | --- | --- | --- | --- | --- | --- | --- | --- |
| P01 | AMP | AMP | AMP | AMP | Non-AMP | Non-AMP | 4/6 | AMP |
| P02 | AMP | AMP | AMP | AMP | AMP | AMP | 6/6 | AMP |
| P03 | AMP | AMP | AMP | AMP | AMP | AMP | 6/6 | AMP |
| P04 | AMP | Non-AMP | AMP | AMP | AMP | AMP | 5/6 | AMP |
| P05 | AMP | AMP | AMP | AMP | Non-AMP | Non-AMP | 4/6 | AMP |
| P06 | AMP | Non-AMP | Non-AMP | AMP | AMP | Non-AMP | 3/6 | Non-AMP |
| P07 | AMP | AMP | AMP | AMP | AMP | AMP | 6/6 | AMP |
| P08 | AMP | Non-AMP | AMP | Non-AMP | AMP | AMP | 4/6 | AMP |
| P09 | Non-AMP | Non-AMP | Non-AMP | Non-AMP | Non-AMP | Non-AMP | 0/6 | Non-AMP |
| P10 | AMP | AMP | AMP | AMP | AMP | AMP | 6/6 | AMP |
| P11 | Non-AMP | Non-AMP | Non-AMP | Non-AMP | Non-AMP | Non-AMP | 0/6 | Non-AMP |
| P12 | AMP | AMP | AMP | AMP | AMP | AMP | 6/6 | AMP |
| P13 | AMP | AMP | AMP | AMP | Non-AMP | AMP | 5/6 | AMP |
| P14 | AMP | AMP | AMP | AMP | AMP | Non-AMP | 5/6 | AMP |
| P15 | AMP | Non-AMP | AMP | AMP | AMP | Non-AMP | 4/6 | AMP |

Note: CAMPR4 consensus was defined as AMP when ≥4 of 6 classifiers predicted antimicrobial activity. Classifiers include RF, SVM and ANN models trained on natural AMP datasets and synthetic AMP datasets, respectively.

**Supplementary Figures**

**Supplementary Figure S1. Overview of dataset construction.** Dataset construction workflow. Antimicrobial peptide (AMP) sequences were collected from multiple public repositories and UniProt and subsequently integrated into the iAMPCN database for unified curation and functional annotation. During integration, inconsistent labels across different sources were reconciled by prioritizing experimentally supported positive annotations to correct original negative entries. After quality filtering and redundancy elimination, a benchmark dataset was constructed for AMP versus non-AMP discrimination, and a strictly independent validation dataset was generated to assess generalization performance. AMP sequences were further annotated with multiple efficacy- and toxicity-related functional categories for multifunctional prediction.

**
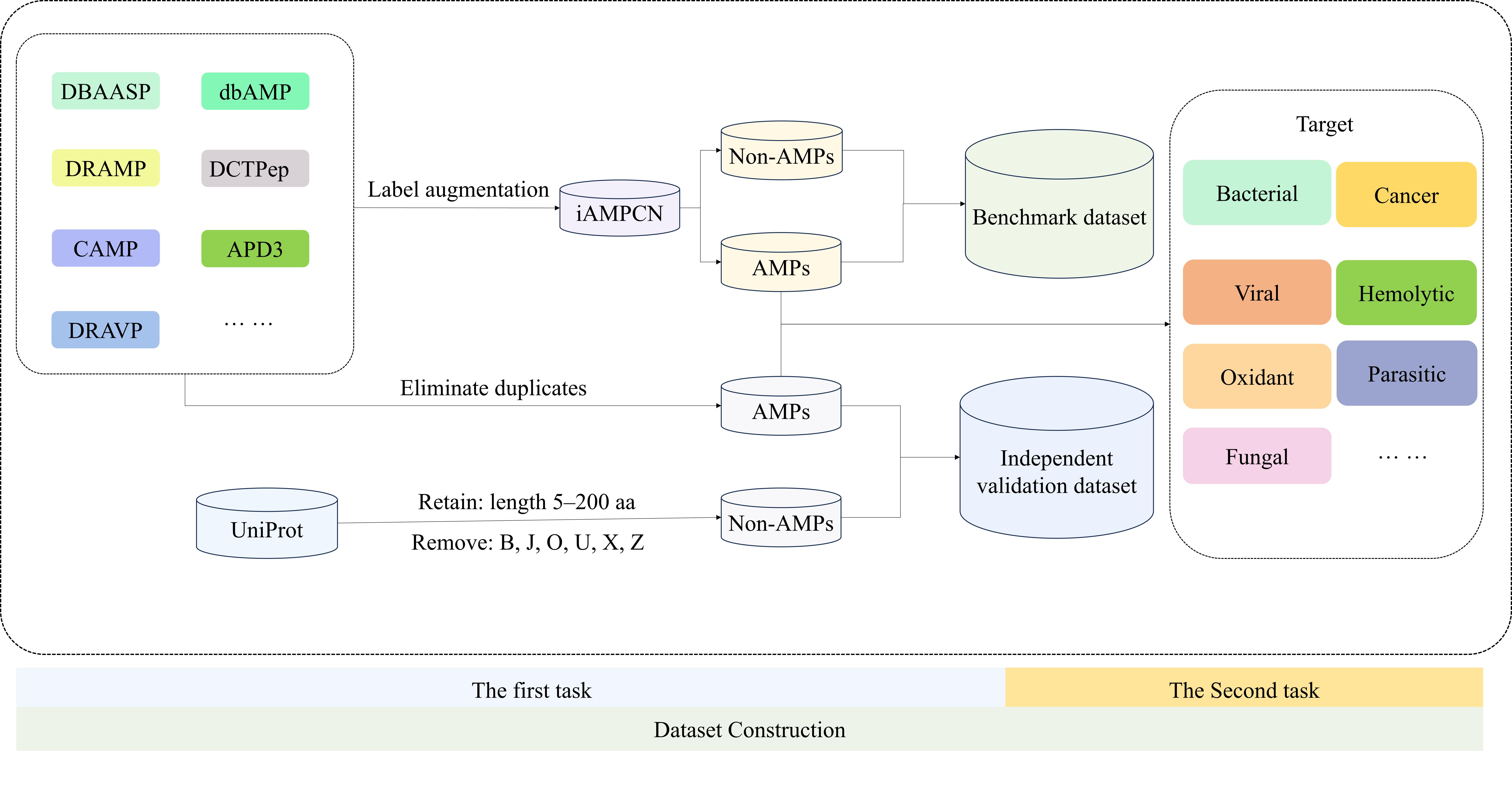
**

**Supplementary Figure S2. Label prevalence, multi-label co-annotation, and functional co-occurrence structure in the benchmark dataset.** (A) Prevalence of each functional label among AMPs, reported as the percentage of AMPs positive for each label. Labels are denoted by letters A–N: (A) anti-mammalian cells, (B) antibacterial, (C) antibiofilm, (D) anticancer, (E) antifungal, (F) antigram-negative, (G) antigram-positive, (H) anti-HIV, (I) anti-MRSA, (J) antioxidant, (K) antiparasitic, (L) antiviral, (M) cytotoxic, and (N) hemolytic. (B) Distribution of the number of positive functional labels per AMP, illustrating the extent of multifunctional annotation. (C) Proportion of AMPs annotated with exactly one label versus two or more labels. (D) Pairwise functional co-occurrence quantified as conditional probability $P(j | i)$, where each entry indicates the probability that an AMP annotated with label i also carries label j (rows: conditioning label i; columns: co-occurring label j).


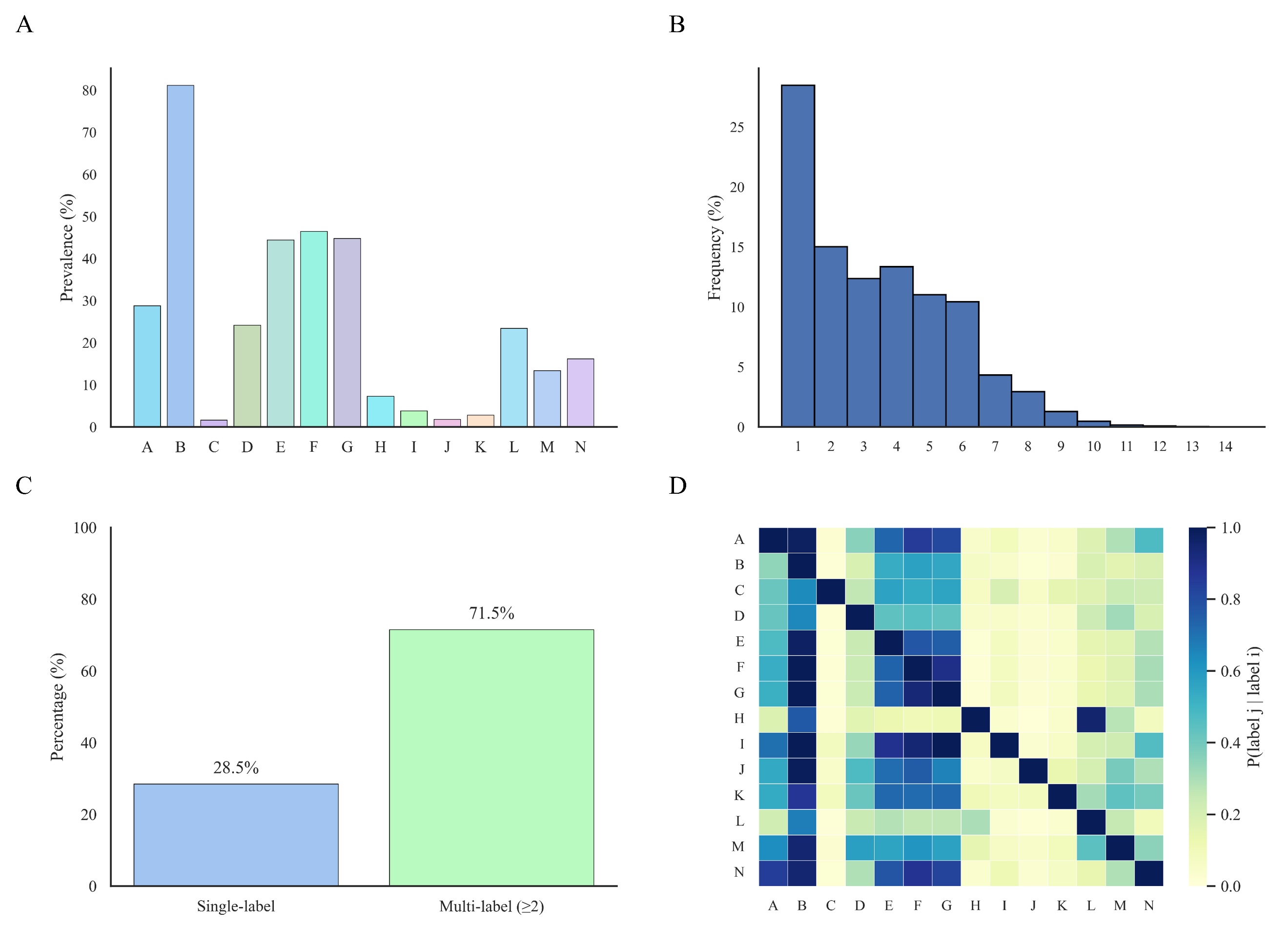


**Supplementary Figure S3. Label prevalence, multi-label co-annotation, and functional co-occurrence structure in the independent validation dataset.** (A) Prevalence of each functional label among AMPs, reported as the percentage of AMPs positive for each label. Labels are denoted by letters A–N: (A) anti-mammalian cells, (B) antibacterial, (C) antibiofilm, (D) anticancer, (E) antifungal, (F) antigram-negative, (G) antigram-positive, (H) anti-HIV, (I) anti-MRSA, (J) antioxidant, (K) antiparasitic, (L) antiviral, (M) cytotoxic, and (N) hemolytic. (B) Distribution of the number of positive functional labels per AMP, illustrating the extent of multifunctional annotation. (C) Proportion of AMPs annotated with exactly one label versus two or more labels. (D) Pairwise functional co-occurrence quantified as conditional probability $P(j | i)$, where each entry indicates the probability that an AMP annotated with label i also carries label j (rows: conditioning label i; columns: co-occurring label j).

**
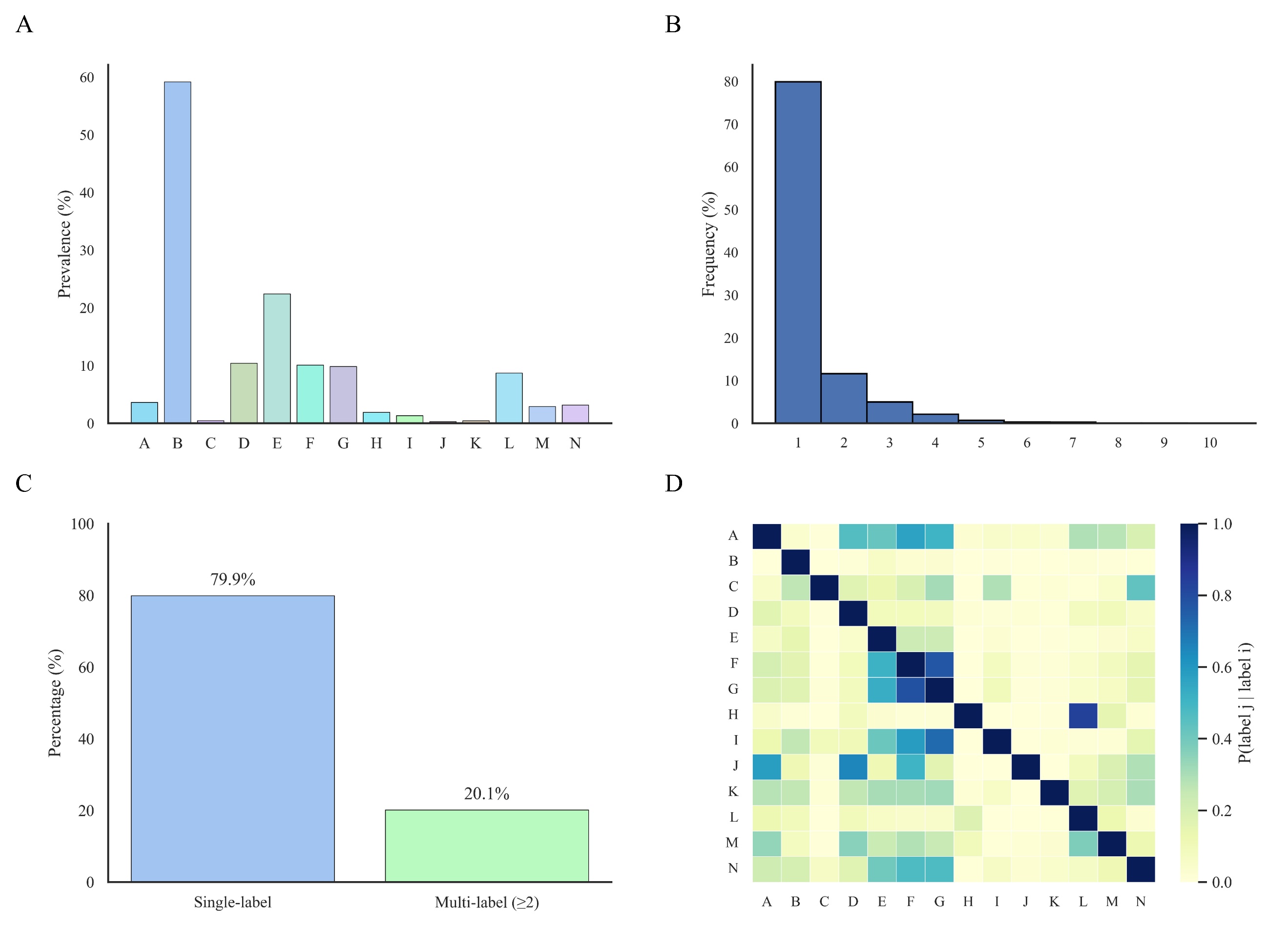
**

**Supplementary Figure S4. Sequence length and compositional characteristics of antimicrobial and non-antimicrobial peptides in the benchmark dataset.** (A) Sequence length distribution of AMP and non-AMP sequences. (B) Amino acid composition comparison between AMP and non-AMP sequences. (C) Violin plots of sequence length across 14 functional categories. (D) Heatmap of normalized amino acid frequencies across functional classes. AMP, Antimicrobial peptides.

**

**

**Supplementary Figure S5. Sequence analysis of** **the independent validation dataset.** (A) Sequence length distribution of AMP and non-AMP sequences. (B) Amino acid composition comparison between AMP and non-AMP sequences. (C) Violin plots of sequence length across 14 functional categories. (D) Heatmap of normalized amino acid frequencies across functional classes. AMP, Antimicrobial peptides.

**

**

**Supplementary Figure S6.** **Comprehensive evaluation of AMP identification models on the benchmark dataset.** Performance comparisons between MAPLE and state-of-the-art predictors are illustrated through (A) ROC curves and (B) PR curves, alongside a quantitative evaluation based on (C) Accuracy, (D) Sensitivity, (E) Specificity, (F) Precision, (G) F1 score, and (H) Matthews correlation coefficient (MCC).

**

**

**Supplementary Figure S7. Dual prediction of antibacterial activity and hemolytic activity enables therapeutic selectivity assessment.** (A) Predicted antibacterial versus hemolytic probability space with risk tiers and decision thresholds. (B) Distribution of hemolytic risk tiers among predicted antibacterial peptides. (C) Proportions with 95% confidence intervals. (D) Cumulative distributions of hemolytic probability. (E) Comparison of hemolytic probability below and above the antibacterial threshold.

**
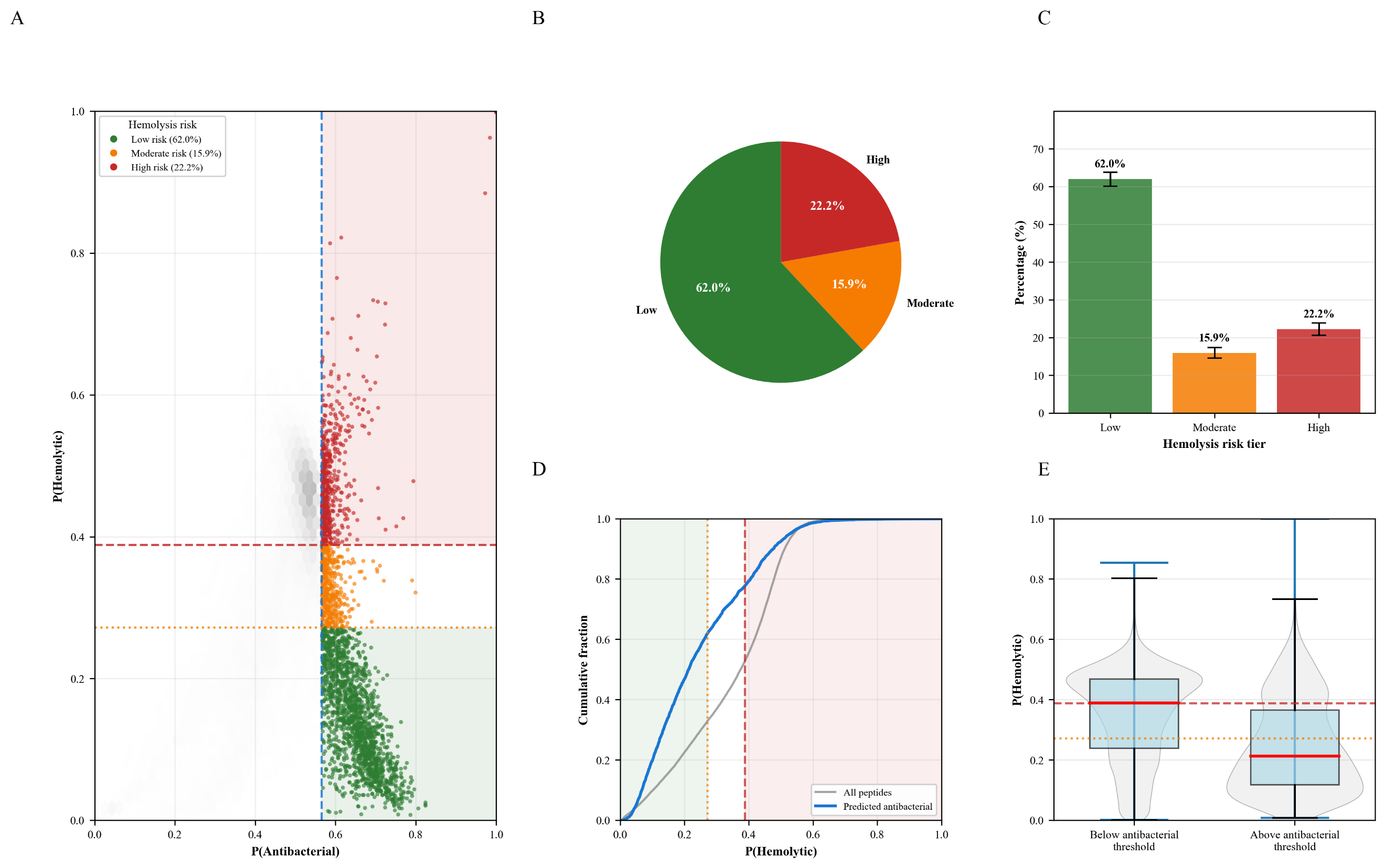
**

**Supplementary Figure S8. UMAP visualization of antibacterial and hemolytic peptides in the fused embedding space.** Blue and red points denote antibacterial and hemolytic peptides, respectively, with kernel density contours indicating their spatial distributions. Cluster centroids are marked, and the dashed line denotes the inter-centroid distance.

**
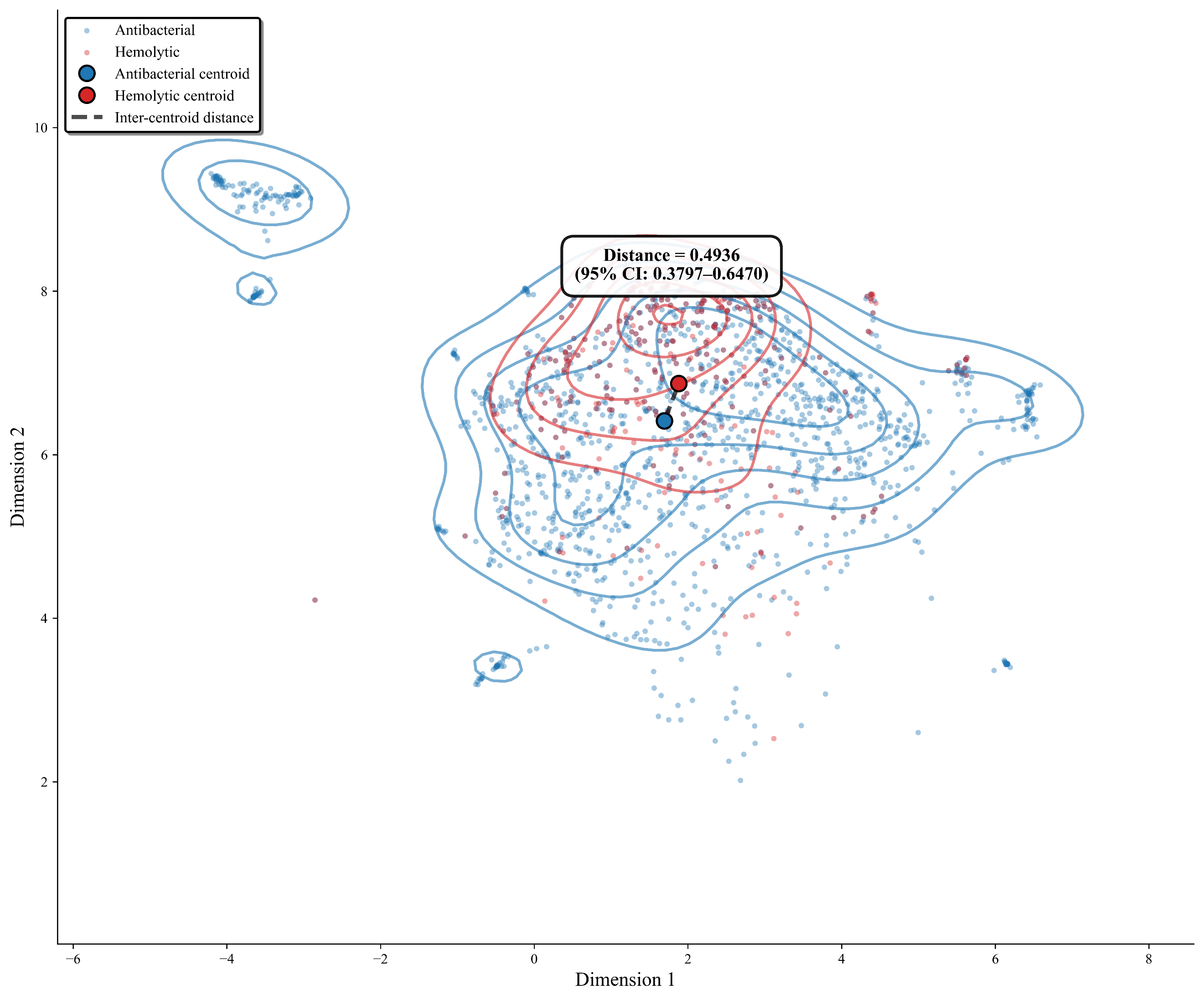
**

**Supplementary Figure S9. K-mer length optimization for functional specificity.** Comparative analysis of k-mer lengths (3-7) across 14 functional categories. (A) Significant k-mer counts across k-mer lengths. (B) Enrichment magnitude increases with k-mer length. (C) Cross-category overlap matrices. (D) Representative motif-extension lineage showing progressive functional specialization from 3-mer to 7-mer motifs. (E) Trade-off between sequence coverage and functional specificity.


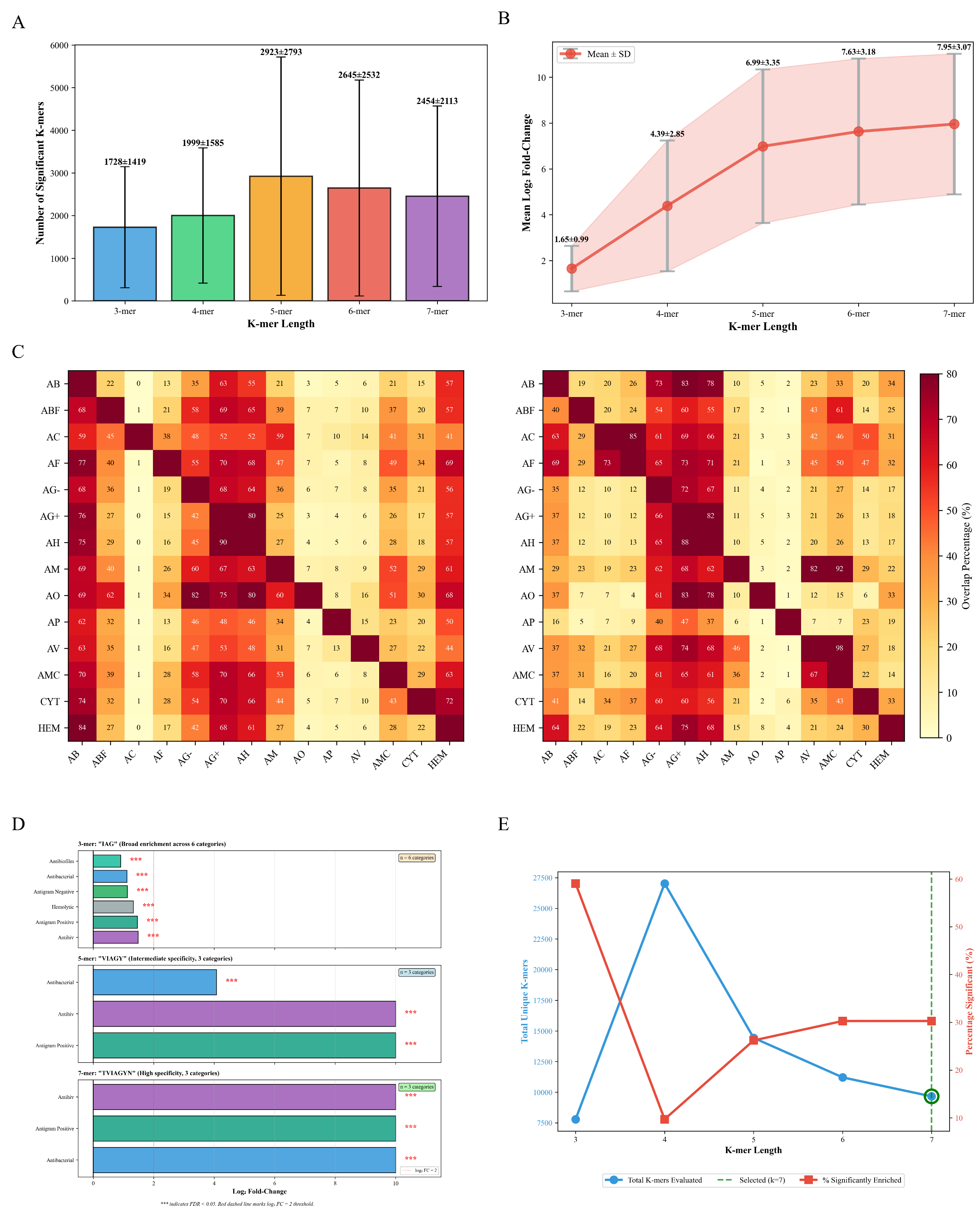


**Supplementary Figure S10. Global enrichment landscapes for antibacterial and hemolytic 7-mers. Side-by-side volcano plots for antibacterial (A) and hemolytic (B) enrichment results.** Selectively enriched 7-mers are highlighted in blue (antibacterial) or orange (hemolytic), whereas 7-mers significantly enriched in both categories are shown in purple. Vertical dashed lines indicate the fold-change threshold and the horizontal dashed line indicates the adjusted significance cutoff.


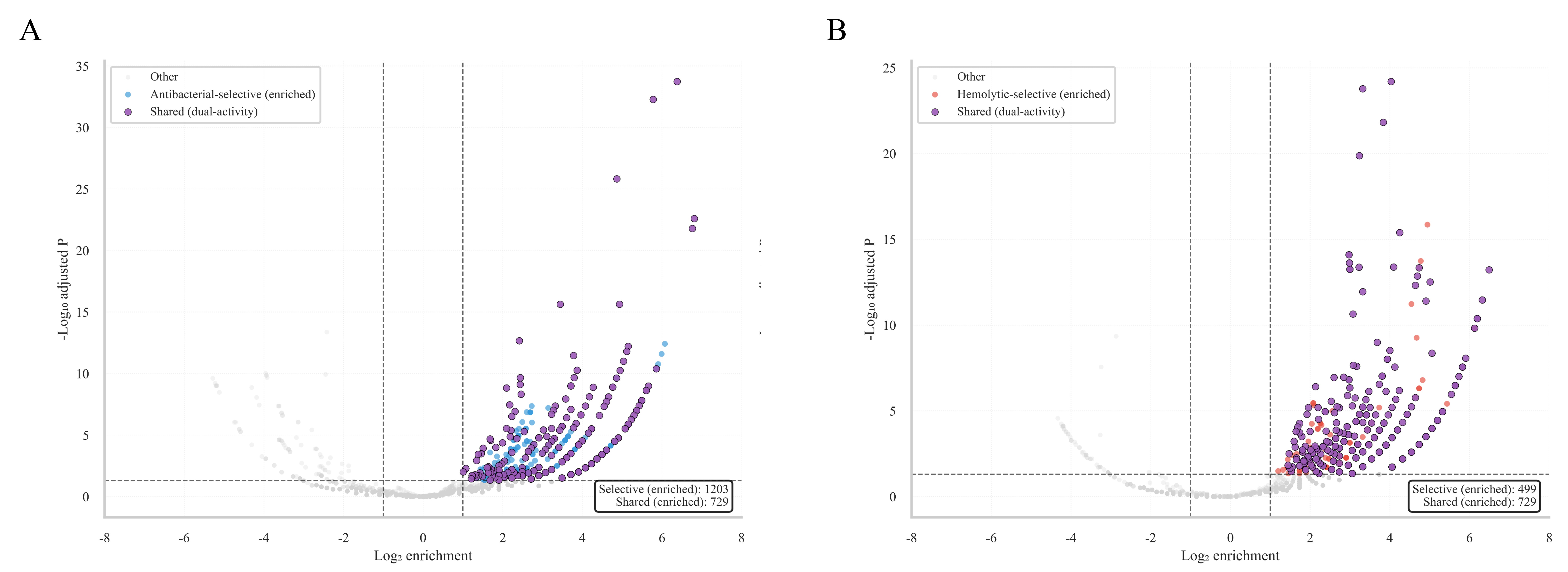


**Supplementary Figure S11. K-mer enrichment patterns across additional functional categories.** Volcano plots show 7-mer enrichment for eight functions: (A) antigram-negative, (B) antigram-positive, (C) anti-MRSA, (D) antibiofilm, (E) anti-HIV, (F) antiparasitic, (G) antioxidant, and (H) cytotoxic. Red points indicate significantly enriched motifs (FDR < 0.05).


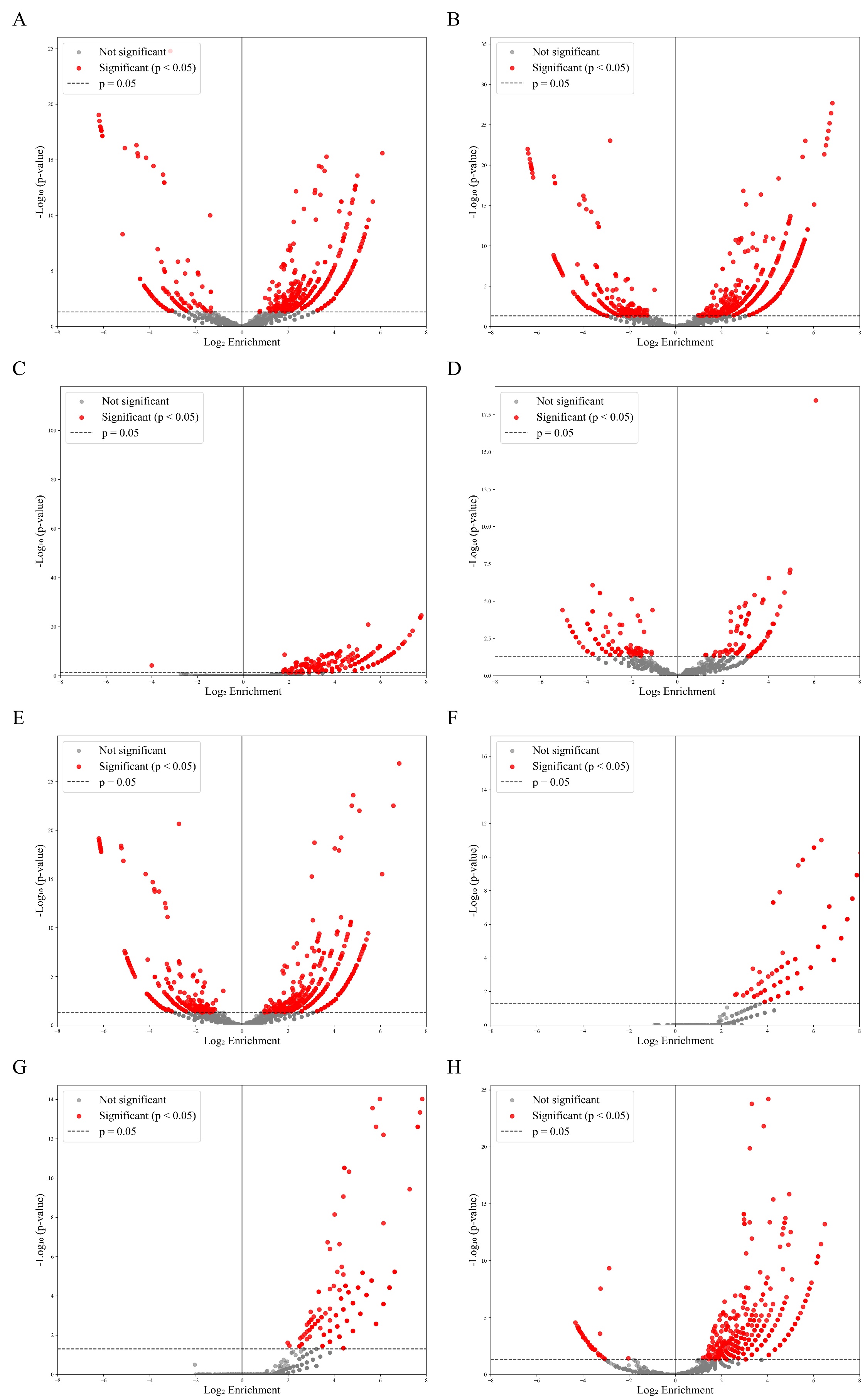


**Supplementary Figure S12. Sequence logos of antibacterial-selective, hemolytic-selective, and dual-activity motifs.** Sequence logos for 7-mer motifs showing antibacterial selectivity (A–C), hemolytic selectivity (D–F), and dual activity (G–I). Logos are shown as frequency-based, information-content, and enrichment-weighted representations.


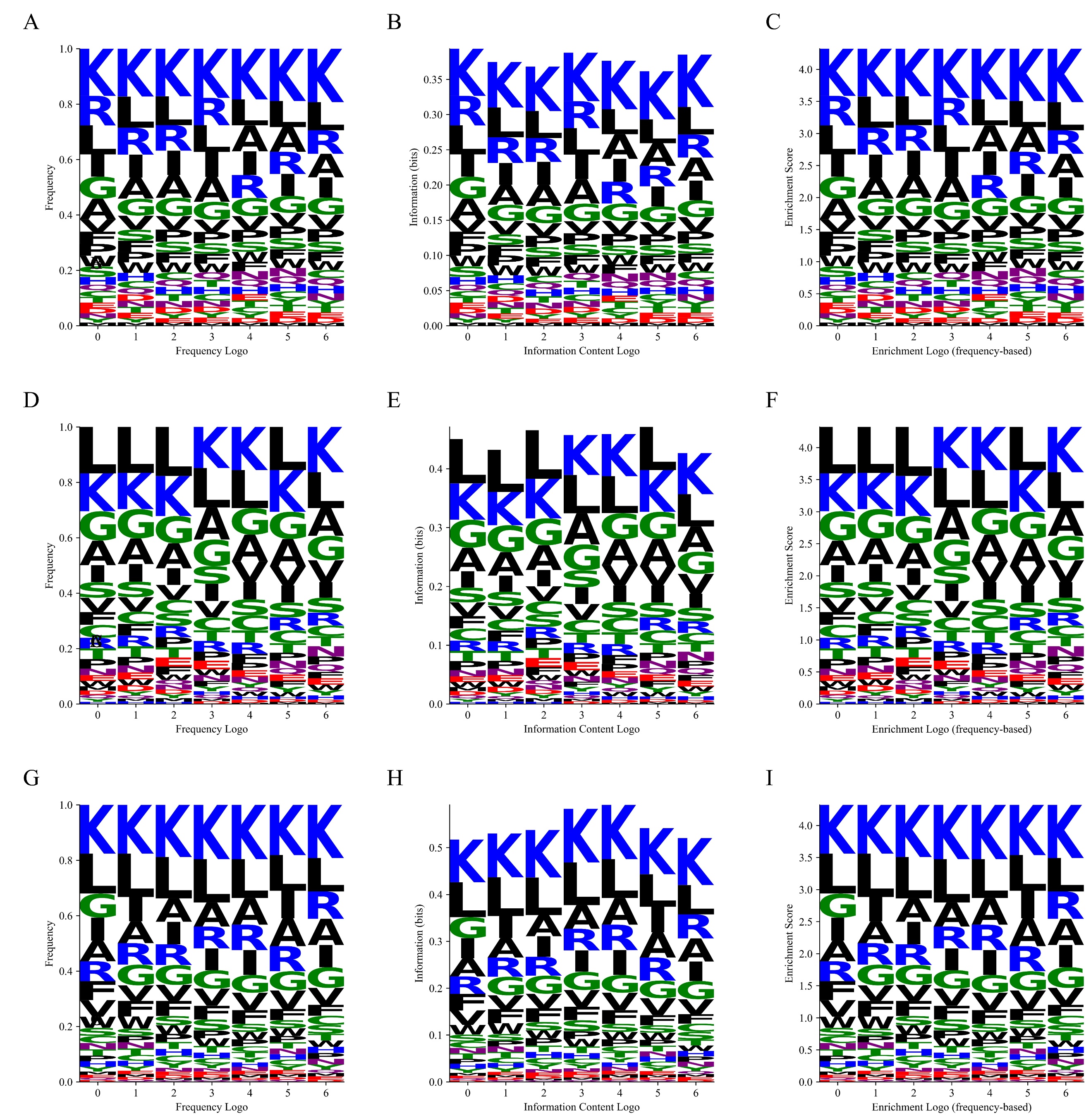


**References**

1. Yao L, Guan J, Xie P, Chung CR, Zhao Z, Dong D, et al. dbAMP 3.0: updated resource of antimicrobial activity and structural annotation of peptides in the post-pandemic era. Nucleic Acids Res. 2025;53(D1):D364-D376.

2. Ma T, Liu Y, Yu B, Sun X, Yao H, Hao C, et al. DRAMP 4.0: an open-access data repository dedicated to the clinical translation of antimicrobial peptides. Nucleic Acids Res. 2025;53(D1):D403-D410.

3. Gawde U, Chakraborty S, Waghu FH, Barai RS, Khanderkar A, Indraguru R, et al. CAMPR4: a database of natural and synthetic antimicrobial peptides. Nucleic Acids Res. 2023;51(D1):D377-D383.

4. Wang G, Li X, Wang Z. APD3: the antimicrobial peptide database as a tool for research and education. Nucleic Acids Res. 2016;44(D1):D1087-1093.

5. Zhang J, Sun X, Zhao H, Zhou X, Zhang Y, Xie F, et al. In Silico Design and Synthesis of Antifungal Peptides Guided by Quantitative Antifungal Activity. J Chem Inf Model. 2024;64(10):4277-4285.

6. Pirtskhalava M, Amstrong AA, Grigolava M, Chubinidze M, Alimbarashvili E, Vishnepolsky B, et al. DBAASP v3: database of antimicrobial/cytotoxic activity and structure of peptides as a resource for development of new therapeutics. Nucleic Acids Res. 2021;49(D1):D288-D297.

7. Mondal RK, Sen D, Arya A, Samanta SK. Developing anti-microbial peptide database version 1 to provide comprehensive and exhaustive resource of manually curated AMPs. Scientific reports. 2023;13(1):17843.

8. Sun X, Liu Y, Ma T, Zhu N, Lao X, Zheng H. DCTPep, the data of cancer therapy peptides. Sci Data. 2024;11(1):541.

9. Liu Y, Zhu Y, Sun X, Ma T, Lao X, Zheng H. DRAVP: A Comprehensive Database of Antiviral Peptides and Proteins. Viruses. 2023;15(4).

10. Lazzaro BP, Zasloff M, Rolff J. Antimicrobial peptides: Application informed by evolution. Science (New York, NY). 2020;368(6490).

11. Dini I, De Biasi MG, Mancusi A. An Overview of the Potentialities of Antimicrobial Peptides Derived from Natural Sources. Antibiotics (Basel, Switzerland). 2022;11(11).

12. Xu J, Li F, Li C, Guo X, Landersdorfer C, Shen HH, et al. iAMPCN: a deep-learning approach for identifying antimicrobial peptides and their functional activities. Briefings in bioinformatics. 2023;24(4).

13. Li W, Godzik A. Cd-hit: a fast program for clustering and comparing large sets of protein or nucleotide sequences. Bioinformatics (Oxford, England). 2006;22(13):1658-1659.

14. Huang Y, Niu B, Gao Y, Fu L, Li W. CD-HIT Suite: a web server for clustering and comparing biological sequences. Bioinformatics (Oxford, England). 2010;26(5):680-682.

15. Fu L, Niu B, Zhu Z, Wu S, Li W. CD-HIT: accelerated for clustering the next-generation sequencing data. Bioinformatics (Oxford, England). 2012;28(23):3150-3152.

16. Chung CR, Kuo TR, Wu LC, Lee TY, Horng JT. Characterization and identification of antimicrobial peptides with different functional activities. Briefings in bioinformatics. 2019;21(3):1098-1114.

17. Wang G. Bioinformatic Analysis of 1000 Amphibian Antimicrobial Peptides Uncovers Multiple Length-Dependent Correlations for Peptide Design and Prediction. Antibiotics (Basel, Switzerland). 2020;9(8).

18. Torres MT, de la Fuente-Nunez C. Toward computer-made artificial antibiotics. Current opinion in microbiology. 2019;51:30-38.

19. UniProt C. UniProt: a hub for protein information. Nucleic Acids Res. 2015;43(Database issue):D204-212.

20. UniProt C. UniProt: a worldwide hub of protein knowledge. Nucleic Acids Res. 2019;47(D1):D506-D515.

21. UniProt C. The universal protein resource (UniProt). Nucleic Acids Res. 2008;36(Database issue):D190-195.

22. UniProt C. UniProt: the Universal Protein Knowledgebase in 2025. Nucleic Acids Res. 2025;53(D1):D609-D617.

23. Torres MDT, Melo MCR, Flowers L, Crescenzi O, Notomista E, de la Fuente-Nunez C. Mining for encrypted peptide antibiotics in the human proteome. Nature biomedical engineering. 2022;6(1):67-75.

24. Lin Z, Akin H, Rao R, Hie B, Zhu Z, Lu W, et al. Evolutionary-scale prediction of atomic-level protein structure with a language model. Science. 2023;379(6637):1123-1130.

25. Kyte J, Doolittle RF. A simple method for displaying the hydropathic character of a protein. Journal of molecular biology. 1982;157(1):105-132.

26. Chou PY, Fasman GD. Prediction of protein conformation. Biochemistry. 1974;13(2):222-245.

27. Fjell CD, Hiss JA, Hancock RE, Schneider G. Designing antimicrobial peptides: form follows function. Nature reviews Drug discovery. 2011;11(1):37-51.

28. Dennison SR, Whittaker M, Harris F, Phoenix DA. Anticancer alpha-helical peptides and structure/function relationships underpinning their interactions with tumour cell membranes. Current protein & peptide science. 2006;7(6):487-499.

29. Boman HG. Antibacterial peptides: basic facts and emerging concepts. Journal of internal medicine. 2003;254(3):197-215.

30. Vaswani A, Shazeer N, Parmar N, Uszkoreit J, Jones L, Gomez AN, et al., editors. Attention Is All You Need. Advances in Neural Information Processing Systems 30 (NeurIPS 2017); 2017 December 4-9, 2017; Long Beach, CA, USA. Long Beach, CA, USA: Neural Information Processing Systems Foundation.

31. Du Z, Ding X, Xu Y, Li Y. UniDL4BioPep: a universal deep learning architecture for binary classification in peptide bioactivity. Briefings in bioinformatics. 2023;24(3).

32. Gu A, Dao T, editors. Mamba: Linear-Time Sequence Modeling with Selective State Spaces. First Conference on Language Modeling; 2024.

33. Gu A, Goel K, Ré C. Efficiently modeling long sequences with structured state spaces. International Conference on Learning Representations (ICLR)2022.

34. Dao T, Gu A. Transformers are SSMs: Generalized models and efficient algorithms through structured state space duality. Proceedings of the 41st International Conference on Machine Learning (ICML): PMLR; 2024. p. 9859-9923.

35. Hendrycks D, Gimpel K. Gaussian error linear units (GELUs). arXiv preprint arXiv:160608415. 2016.

36. Li J, Wen Y, He L. SCConv: Spatial and Channel Reconstruction Convolution for Feature Redundancy. Proceedings of the IEEE/CVF Conference on Computer Vision and Pattern Recognition (CVPR): IEEE; 2023. p. 6153-6162.

37. Wu Y, He K. Group Normalization. Computer Vision – ECCV 2018: 15th European Conference, Munich, Germany, September 8-14, 2018, Proceedings, Part XIII; Munich, Germany: Springer-Verlag; 2018. p. 3–19.

38. Lecun Y, Bottou L, Bengio Y, Haffner P. Gradient-based learning applied to document recognition. Proceedings of the IEEE. 1998;86(11):2278-2324.

39. Wan F, Torres MDT, Peng J, de la Fuente-Nunez C. Deep-learning-enabled antibiotic discovery through molecular de-extinction. Nature biomedical engineering. 2024;8(7):854-871.

40. Wan F, Wong F, Collins JJ, de la Fuente-Nunez C. Machine learning for antimicrobial peptide identification and design. Nature reviews bioengineering. 2024;2(5):392-407.

41. Hu J, Shen L, Sun G. Squeeze-and-excitation networks. Proceedings of the IEEE Conference on Computer Vision and Pattern Recognition (CVPR): IEEE; 2018. p. 7132-7141.

42. Tsai Y-HH, Bai S, Liang PP, Kolter JZ, Morency L-P, Salakhutdinov R, editors. Multimodal Transformer for Unaligned Multimodal Language Sequences2019 July; Florence, Italy: Association for Computational Linguistics.

43. Lu J, Batra D, Parikh D, Lee S. ViLBERT: pretraining task-agnostic visiolinguistic representations for vision-and-language tasks. Proceedings of the 33rd International Conference on Neural Information Processing Systems: Curran Associates Inc.; 2019. p. Article 2.

44. Pang Y, Yao L, Xu J, Wang Z, Lee TY. Integrating transformer and imbalanced multi-label learning to identify antimicrobial peptides and their functional activities. Bioinformatics (Oxford, England). 2022;38(24):5368-5374.

45. Zhenghui L, Wenxing H, Yan W, Jihong Z, Xiaojun X, Lixin G, et al. Ensemble learning based on bi-directional gated recurrent unit and convolutional neural network with word embedding module for bioactive peptide prediction. Food Chemistry. 2025;468:142464.

46. McInnes L, Healy J, Saul N, Großberger L. UMAP: Uniform Manifold Approximation and Projection. Journal of Open Source Software. 2018;3(29):861.

47. Torres MDT, Brooks EF, Cesaro A, Sberro H, Gill MO, Nicolaou C, et al. Mining human microbiomes reveals an untapped source of peptide antibiotics. Cell. 2024;187(19):5453-5467 e5415.

48. Chang CH, Nelson WC, Jerger A, Wright AT, Egbert RG, McDermott JE. Snekmer: a scalable pipeline for protein sequence fingerprinting based on amino acid recoding. Bioinform Adv. 2023;3(1):vbad005.

49. Moeckel C, Mareboina M, Konnaris MA, Chan CSY, Mouratidis I, Montgomery A, et al. A survey of k-mer methods and applications in bioinformatics. Comput Struct Biotechnol J. 2024;23:2289-2303.

50. McLeay RC, Bailey TL. Motif Enrichment Analysis: a unified framework and an evaluation on ChIP data. BMC Bioinformatics. 2010;11(1):165.

51. Benjamini Y, Hochberg Y. Controlling the False Discovery Rate: A Practical and Powerful Approach to Multiple Testing. Journal of the Royal Statistical Society Series B: Statistical Methodology. 1995;57(1):289-300.

52. Wang G. The antimicrobial peptide database is 20 years old: Recent developments and future directions. Protein Sci. 2023;32(10):e4778.

53. Zhang L, Bao M, Liu B, Zhao H, Zhang Y, Ji X. The antimicrobial peptides and their potential clinical applications. American Journal of Translational Research. 2019;11(7):3919-3931.

54. Gagat P, Ostrowka M, Duda-Madej A, Mackiewicz P. Enhancing Antimicrobial Peptide Activity through Modifications of Charge, Hydrophobicity, and Structure. Int J Mol Sci. 2024;25(19).

55. Dilawari R, Chaubey GK, Priyadarshi N, Barik M, Parmar N. Antimicrobial peptides: structure, function, mechanism of action and therapeutic applications in human diseases. Exploration of Drug Science. 2025;3:1008110.

56. Ebbensgaard A, Mordhorst H, Aarestrup FM, Hansen EB. The Role of Outer Membrane Proteins and Lipopolysaccharides for the Sensitivity of Escherichia coli to Antimicrobial Peptides. Front Microbiol. 2018;9:2153.

57. Gallardo-Becerra L, Cervantes-Echeverria M, Cornejo-Granados F, Vazquez-Morado LE, Ochoa-Leyva A. Perspectives in Searching Antimicrobial Peptides (AMPs) Produced by the Microbiota. Microb Ecol. 2023;87(1):8.

58. Lin TT, Yang LY, Lu IH, Cheng WC, Hsu ZR, Chen SH, et al. AI4AMP: an Antimicrobial Peptide Predictor Using Physicochemical Property-Based Encoding Method and Deep Learning. mSystems. 2021;6(6):e0029921.

59. Li C, Zou Q, Jia C, Zheng J. AMPpred-MFA: An Interpretable Antimicrobial Peptide Predictor with a Stacking Architecture, Multiple Features, and Multihead Attention. J Chem Inf Model. 2024;64(7):2393-2404.

60. Zhang L, Xiong S, Xu L, Liang J, Zhao X, Zhang H, et al. Leveraging protein language models for robust antimicrobial peptide detection. Methods (San Diego, Calif). 2025;238:19-26.

61. Randall JR, Vieira LC, Wilke CO, Davies BW. Deep mutational scanning and machine learning for the analysis of antimicrobial-peptide features driving membrane selectivity. Nat Biomed Eng. 2024;8(7):842-853.

62. Rathore AS, Kumar N, Choudhury S, Mehta NK, Raghava GPS. Prediction of hemolytic peptides and their hemolytic concentration. Communications biology. 2025;8(1):176.

63. Li T, Ren X, Luo X, Wang Z, Li Z, Luo X, et al. A Foundation Model Identifies Broad-Spectrum Antimicrobial Peptides against Drug-Resistant Bacterial Infection. Nature communications. 2024;15(1):7538.

64. Torres MDT, Sothiselvam S, Lu TK, de la Fuente-Nunez C. Peptide Design Principles for Antimicrobial Applications. J Mol Biol. 2019;431(18):3547-3567.

65. Gabere MN, Noble WS. Empirical comparison of web-based antimicrobial peptide prediction tools. Bioinformatics (Oxford, England). 2017;33(13):1921-1929.

66. Bui Thi Phuong H, Doan Ngan H, Le Huy B, Vu Dinh H, Luong Xuan H. The amphipathic design in helical antimicrobial peptides. ChemMedChem. 2024;19(7):e202300480.

67. Dathe M, Wieprecht T. Structural features of helical antimicrobial peptides: their potential to modulate activity on model membranes and biological cells. Biochim Biophys Acta. 1999;1462(1-2):71-87.

68. Sormanni P, Aprile FA, Vendruscolo M. The CamSol method of rational design of protein mutants with enhanced solubility. Journal of molecular biology. 2015;427(2):478-490.

69. Conchillo-Sole O, de Groot NS, Aviles FX, Vendrell J, Daura X, Ventura S. AGGRESCAN: a server for the prediction and evaluation of "hot spots" of aggregation in polypeptides. BMC Bioinformatics. 2007;8:65.

70. Rathore AS, Choudhury S, Arora A, Tijare P, Raghava GPS. ToxinPred 3.0: An improved method for predicting the toxicity of peptides. Comput Biol Med. 2024;179:108926.

71. Vishnepolsky B, Grigolava M, Managadze G, Gabrielian A, Rosenthal A, Hurt DE, et al. Comparative analysis of machine learning algorithms on the microbial strain-specific AMP prediction. Briefings in bioinformatics. 2022;23(4).
